# Structural Conservation Despite Huge Sequence Diversity Allows EPCR Binding by the PfEMP1 Family Implicated in Severe Childhood Malaria

**DOI:** 10.1016/j.chom.2014.11.007

**Published:** 2015-01-14

**Authors:** Clinton K.Y. Lau, Louise Turner, Jakob S. Jespersen, Edward D. Lowe, Bent Petersen, Christian W. Wang, Jens E.V. Petersen, John Lusingu, Thor G. Theander, Thomas Lavstsen, Matthew K. Higgins

**Affiliations:** 1 Department of Biochemistry, University of Oxford, South Parks Road, OX1 3QU Oxford, UK; 2 Centre for Medical Parasitology, Department of International Health, Immunology & Microbiology, University of Copenhagen and Department of Infectious Diseases, Rigshospitalet, 1017 Copenhagen, Denmark; 3 Center for Biological Sequence Analysis, Technical University of Denmark, 2800 Kgs. Lyngby, Denmark; 4 National Institute for Medical Research, Tanga, 2448 Ocean Road, P.O. Box 9653, Dar es Salaan, Tanzania

## Abstract

The PfEMP1 family of surface proteins is central for *Plasmodium falciparum* virulence and must retain the ability to bind to host receptors while also diversifying to aid immune evasion. The interaction between CIDRα1 domains of PfEMP1 and endothelial protein C receptor (EPCR) is associated with severe childhood malaria. We combine crystal structures of CIDRα1:EPCR complexes with analysis of 885 CIDRα1 sequences, showing that the EPCR-binding surfaces of CIDRα1 domains are conserved in shape and bonding potential, despite dramatic sequence diversity. Additionally, these domains mimic features of the natural EPCR ligand and can block this ligand interaction. Using peptides corresponding to the EPCR-binding region, antibodies can be purified from individuals in malaria-endemic regions that block EPCR binding of diverse CIDRα1 variants. This highlights the extent to which such a surface protein family can diversify while maintaining ligand-binding capacity and identifies features that should be mimicked in immunogens to prevent EPCR binding.

## Highlights

• EPCR binding is retained by PfEMP1 CIDRα1 domains despite huge sequence variation
• Diverse CIDRα1 domains retain structural and chemical features to bind to EPCR
• CIDRα1 domains mimic features of a natural ligand of EPCR and block its binding
• Patient sera contain neutralizing antibodies that prevent parasite binding to EPCR

## Graphical Abstract

**Figure:**
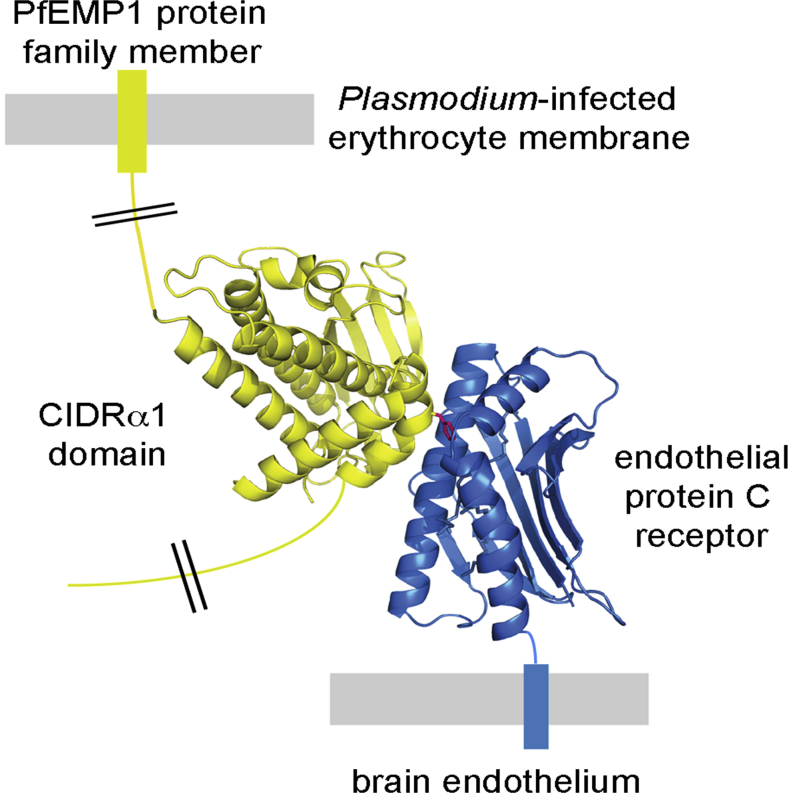

## Teaser

PfEMP1 proteins of *Plasmodium* must retain binding to host receptor EPCR while diversifying for immune evasion. Using structural studies, Lau et al. show that EPCR-binding surfaces of PfEMP1 are conserved in shape and bonding potential, despite sequence diversity, and identify features that should be mimicked in immunogens preventing EPCR binding.

## Introduction

Parasites, such as the *Plasmodium* species that cause malaria, have developed strategies to aid survival in a mammalian host and to multiply in the nutrient-rich blood. They must make specific interactions with host molecules, enabling them to invade cells, acquire nutrients, and populate protected environments. At the same time, they must avoid detection by components of the innate and acquired immune systems. A common evolutionary strategy, employed by many unicellular eukaryotic parasites, is expansive development of a family of surface proteins, which lie at the interface between host and parasite. Examples include PfEMP1 (Leech et al., 1984), RIFIN (Kyes et al., 1999), and STEVOR (Cheng et al., 1998) of *Plasmodium falciparum*, VIR of *P. vivax* (del Portillo et al., 2001), variant surface glycoproteins (VSGs) of *Trypanosoma brucei* (Schwede and Carrington, 2010), MASP (El-Sayed et al., 2005) and SAP (Carmo et al., 2001) of *Trypanosoma cruzi*, and SAGs of *Toxoplasma gondii* (Kasper et al., 1983). Expression switching between family members allows parasites to display a series of antigenically distinct surfaces, posing challenges for the immune system and for rational development of vaccines.

The PfEMP1 protein family of *Plasmodium falciparum* is one of the most closely studied surface protein families, with about 60 members encoded in each genome (Smith et al., 2013; Gardner et al., 2002). They are expressed on the surfaces of infected erythrocytes where they interact with various human endothelial receptors, tethering these erythrocytes to blood vessel or tissue surfaces. This prevents spleen-mediated clearance of the parasite and allows the infection to build. It also leads to the most severe symptoms of the disease, resulting in inflammation of the brain and the placenta during cerebral or pregnancy-associated malaria (Miller et al., 2002). PfEMP1 are therefore under dual selection pressure to retain the ability to bind to the vasculature while diversifying into a family of antigenically distinct proteins.

The extracellular ectodomains of the PfEMP1 proteins contain 2–10 copies of two *Plasmodium*-specific domain types, the Duffy-binding-like (DBL) and cysteine-rich interdomain region (CIDR) domains (Baruch et al., 1995; Smith et al., 1995; Su et al., 1995; Gardner et al., 2002). Individual domains frequently act as discrete ligand-binding modules, with a diverse set of host endothelial surface proteins and carbohydrates identified as partners for different domains (Smith et al., 2013). DBL and CIDR domains have been divided into specific classes based on sequence similarity and the presence of constituent homology blocks (Smith et al., 2000; Rask et al., 2010). Specific domain subclasses interact with specific endothelial receptors (Smith et al., 2000). However, even within a domain subclass, sequence diversity is high, making it challenging to identify conserved functional regions required to mediate binding to a particular receptor based on sequence analysis (Robinson et al., 2003; Howell et al., 2008; Higgins and Carrington, 2014).

Despite significant PfEMP1 sequence diversity, natural immunity to severe malaria is acquired after only one or two severe infections, and immunoglobulin G (IgG) that binds PfEMP1 and prevents adhesion plays a significant role (Bull et al., 1998; Salanti et al., 2004; Lusingu et al., 2006; Cham et al., 2009; Gupta et al., 1999; Nielsen et al., 2002; Gonçalves et al., 2014). This raises hope that it will be possible to develop a vaccine to mimic this natural immunity and to prevent severe disease. However, such a vaccine must raise antibodies that recognize a diverse set of PfEMP1 proteins, and rational design of constituent immunogens requires an understanding of this diversity and detailed knowledge of the structures of conserved features that should be targeted by inhibitory antibodies. The lack of a structure of a PfEMP1 protein domain in complex with a protein ligand has made such an analysis impossible.

In this study, we have combined sequence analysis with structural and biochemical studies to determine the extent to which PfEMP1 domains that interact with a particular receptor can diversify and to identify features that remain conserved. We have focused on the interaction between CIDRα1 domains and endothelial protein C receptor (EPCR), as the expression of CIDRα1-containing PfEMP1s and the EPCR-binding phenotype are both associated with severe childhood malaria (Lavstsen et al., 2012; Turner et al., 2013). Indeed, the key role of this interaction in malaria pathogenesis is substantiated by the discovery of altered brain endothelial EPCR expression in cerebral malaria patients (Moxon et al., 2013). Additionally, a polymorphism in the transmembrane domain of EPCR that leads to increased plasma levels of soluble receptor also associates with protection from severe malaria in a Thai population (Naka et al., 2014). Here, we show that EPCR-binding CIDRα1 domains are extremely diverse, even in the residues that directly contact EPCR. However, we find that conserved structural features with conserved bonding potential are retained to maintain this binding phenotype. This shows the extent to which such a parasite protein family can diversify while retaining high-affinity ligand binding and characterizes the features that should be targeted in development of therapeutics to block EPCR binding in severe malaria.

## Results

### Extensive Sequence Diversity among EPCR-Binding CIDRα1 Domains

Endothelial protein C receptor binding was identified as a property of CIDRα1 domain variants found in PfEMP1 proteins containing two particular combinations of domains: domain cassette 8 (DBLα2-CIDRα1.1-DBLβ12-DBLγ4/γ6) and domain cassette 13 (DBLα1.7-CIDRα1.4) (Turner et al., 2013). These cassettes are present in PfEMP1s expressed in a large proportion of tested children suffering from severe malaria (Lavstsen et al., 2012; Bertin et al., 2013) and also in parasites selected for adhesion to brain endothelial cells (Avril et al., 2012; Claessens et al., 2012), suggesting a pivotal role in severe outcomes of *P. falciparum* infections. EPCR binding by PfEMP1s was mapped exclusively to their CIDRα1.1 and CIDRα1.4 domains. Indeed, the CIDRα1.1 domain of the IT4var20 PfEMP1 protein bound to EPCR with an affinity comparable to that of the whole ectodomain (Turner et al., 2013). Other CIDR domain classes, not present in DC8 and DC13 domain cassettes, such as the CIDRα2 and CIDRα3 domains, did not interact with EPCR but bound to CD36 (Turner et al., 2013).

**Figure 1:**
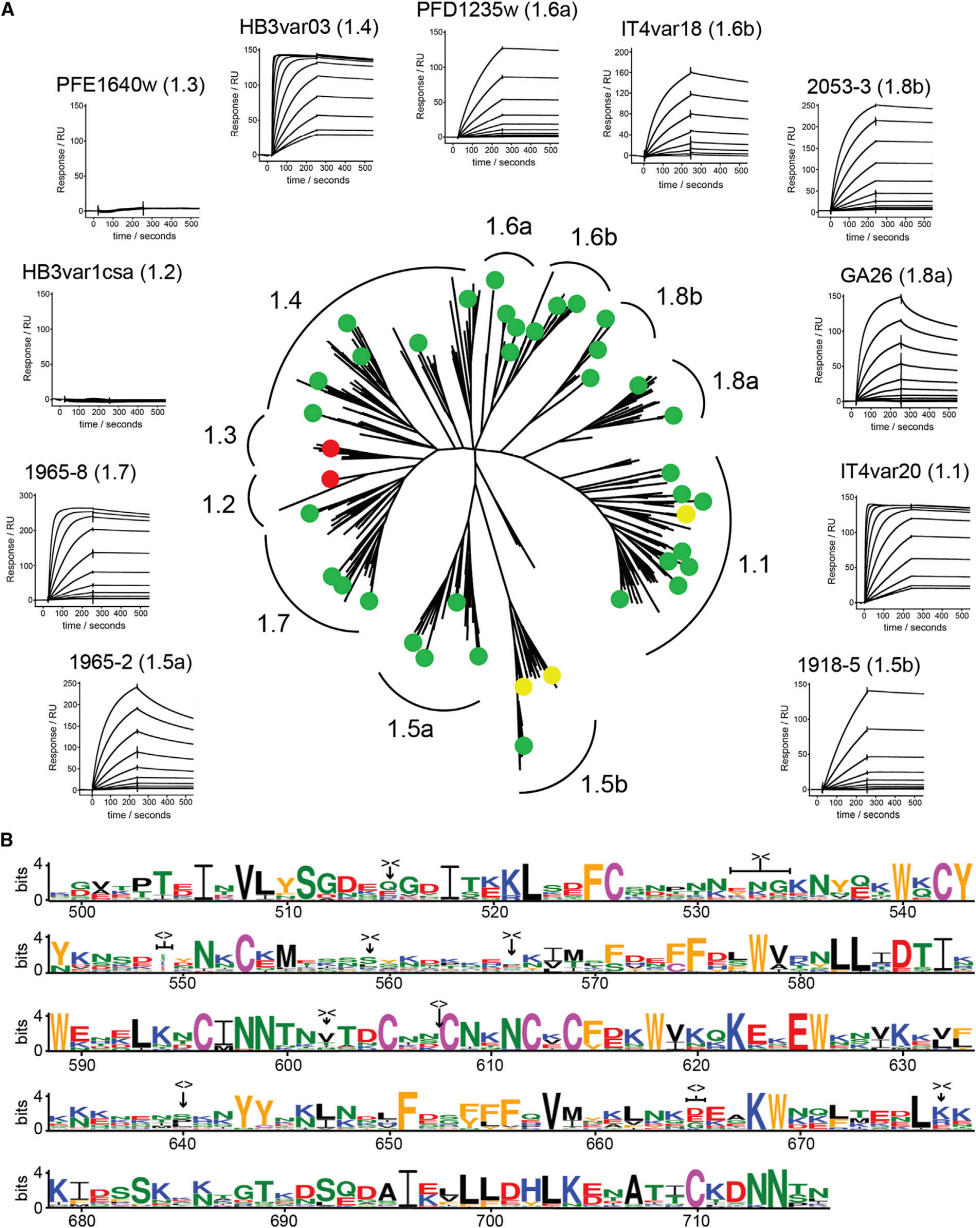
High Sequence Diversity in EPCR-Binding CIDRα1 Domains (A) A maximum likelihood tree (bootstrap n = 50) of 885 full-length CIDRα1 domains used in this study showing branching into previously identified subclasses CIDRα1.1–1.8 and the bipartition of subclasses CIDRα1.5, CIDRα1.6, and CIDRα1.8. Circles represent the degree of EPCR binding by ELISA with positive (green), negative (red), and weakly positive (yellow). Also shown are representative SPR traces for each CIDRα1 subclass showing binding to EPCR. (B) All sequences of CIDRα1 subclasses 1.1 and 1.4–1.8 were aligned, and a sequence logo was generated of residues equivalent to those found in the HB3var03 CIDRα1 domain (numbered as in HB3var03). Deletions (> < ) and insertions (< > ) are indicated as explained in Table S3. See also Figure S1.

To test the depth of diversity of EPCR-binding domains, we expanded our collection of CIDRα1 domain sequences from the previously described 66 sequences, originating mainly from seven parasite genomes (Kraemer and Smith, 2006; Rask et al., 2010), by addition of domain sequences extracted from assemblies of whole-genome sequencing data from 226 parasite isolates collected in both Africa and Asia (Manske et al., 2012), resulting in a total data set of 885 sequences. These domains were grouped, based on phylogenetic analysis, into eight previously defined subclasses (CIDRα1.1–1.8) with an additional splitting of CIDRα1.5, CIDRα1.6, and CIDRα1.8 variants into two, generating CIDRα1.5a/b, CIDRα1.6a/b, and CIDRα1.8a/b (Figure 1A; Table S1, available online). To determine which subclasses contain features required to bind EPCR, members of each subclass, chosen to represent the diversity across CIDRα1 domains, were produced in an insect cell system and tested for binding to EPCR and CD36 by ELISA. All proteins bound to EPCR, with the exception of CIDRα1.2 and CIDRα1.3 domains, which are both found in *var1* genes considered to be pseudogenes (Figures 1A and S1A).

Binding was further characterized by surface plasmon resonance (SPR), allowing determination of binding affinities and kinetic constants. We developed an SPR assay in which EPCR was produced with an N-terminal biotin, allowing coupling to a chip with an orientation matching that found on the cell surface and allowing complete regeneration between measurements. This was used to show that all members of subclasses CIDRα1.1 and CIDRα1.4–1.8 bound to EPCR. The majority of domains bound with high affinities in the range of 0.3–60 nM, but with a few weaker binders (Figures 1A and S1B, Table S2). Despite differences in affinity, it was noticeable that all domains bound with a slow off rate. Indeed, kinetic analysis showed less variation in rate constants for dissociation than in those for association, with a propensity toward slow off rates (Figure S1; Table S2), suggesting that these domains are under selection pressure to form a stable complex with EPCR.

To better understand the degree of diversity of EPCR-binding domains, we analyzed 737 different CIDRα1 sequences from members of the six EPCR-binding subclasses (CIDRα1.1 and CIDRα1.4–1.8). These showed little identity between variants, with just 14 residues (6.5%) absolutely conserved and a further 22 residues conserved in more than 90% of the domains (Figure 1B; Table S3). Most conserved residues are cysteines or aromatics. This is reminiscent of the PfEMP1 DBL domains in which the small percentage of conserved cysteine and aromatic residues are found in the domain core where they play a structural role (Batchelor et al., 2011, 2014; Higgins, 2008; Higgins and Carrington, 2014; Hodder et al., 2012; Khunrae et al., 2010; Lin et al., 2012; Malpede et al., 2013; Singh et al., 2006; Tolia et al., 2005; Vigan-Womas et al., 2012). All residues conserved in the EPCR-binding domains are also totally conserved in CIDRα1.2 and CIDRα1.3 subclasses that do not bind EPCR, showing that CIDRα1 domains are an extremely diverse subfamily that lacks conserved residues that correlate with EPCR binding. We therefore determined cocrystal structures to allow us to understand the molecular basis for EPCR binding and to rationalize how sequence diversity is compatible with the retention of this binding phenotype.

### The Structural Basis for EPCR Binding by PfEMP1s

**Figure 2:**
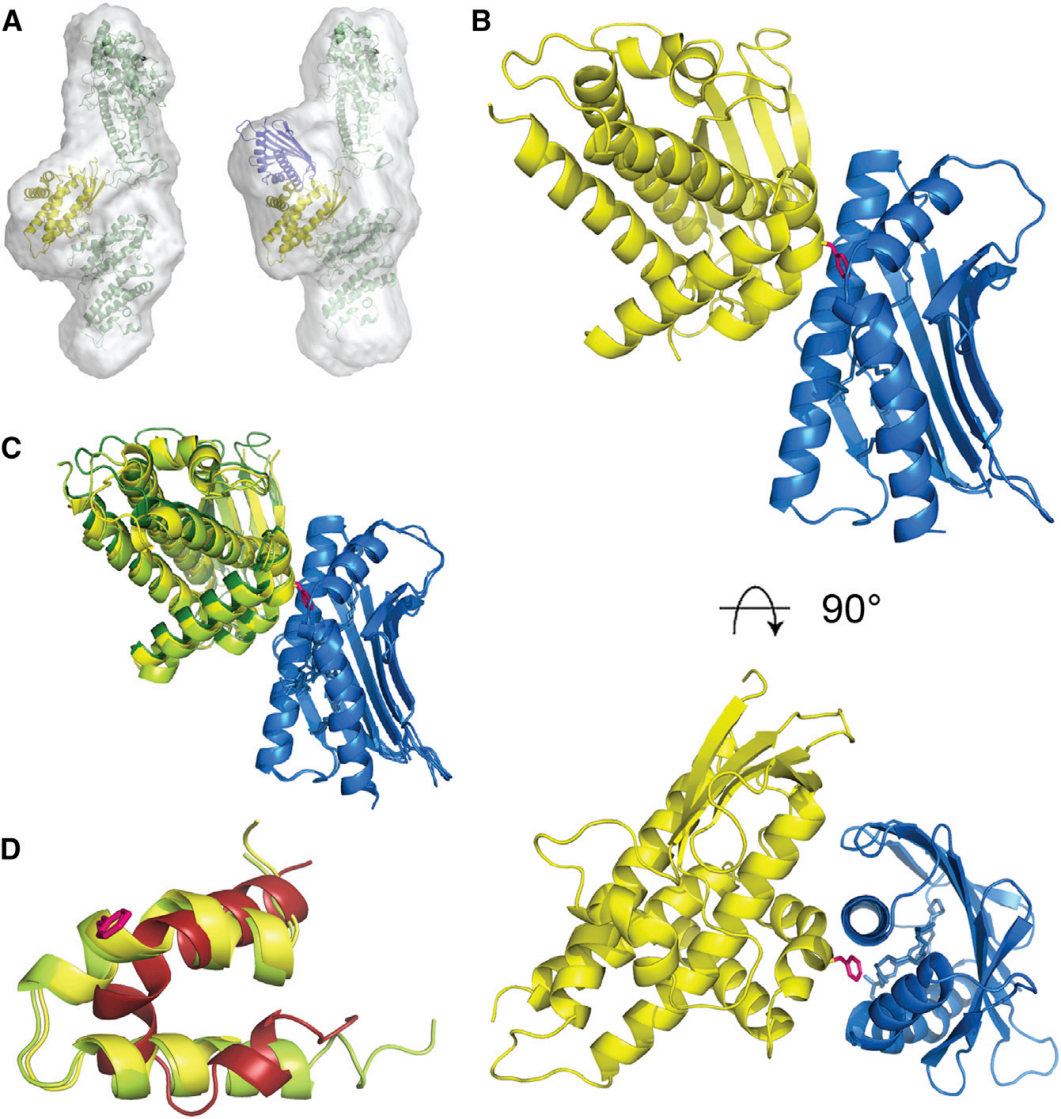
The Structure of the CIDRα1:EPCR Complex (A) Molecular envelopes derived from small-angle X-ray scattering for DD2var32 domains DBLα1.7-CIDRα1.4-DBLβ1 without (left) and with (right) EPCR. (B) Structure of a complex of the HB3var03 CIDRα1 domain (yellow) bound to EPCR (blue). F656 of the CIDRα1 domain is shown as pink sticks. (C) Structural overlay of complexes of EPCR with IT4var07 CIDRα1 (pale green) and the two copies of HB3var03 CIDRα1 (yellow and dark green) found in the crystal. (D) A close up of the EPCR-binding region of HB3var03 CIDRα1 (yellow), IT4var07 CIDRα1 (green), and the equivalent region of var0 CIDRγ (red) reveals the different architecture of the CIDRα1 domains in this region. See also Figure S2.

We have previously shown that a single CIDRα1 domain binds to EPCR with the same affinity as the full-length PfEMP1 protein, demonstrating that EPCR binding capability is contained entirely within CIDRα1 (Turner et al., 2013). Our strategy here was to select a diverse set of domains, increasing the likelihood of identifying a complex that would crystallize, and then combine the structures we obtained with sequence analysis and biophysical studies to rationalize EPCR binding by the protein family. We therefore generated a panel of CIDRα1 domains with domain boundaries appropriate for crystallization and used SPR and isothermal titration calorimetry (ITC) to confirm binding to EPCR with nanomolar affinities and slow dissociation rates (Figures S1 and S2). These domains were reconstituted into complexes with the extracellular domain of EPCR and examined using small-angle X-ray scattering, analytical ultracentrifugation, and multi-angle laser light scattering. In each case, a 1:1 complex formed with no higher-order assemblies observed (Figure S2). Small-angle X-ray scattering of a protein containing the three membrane distal domains of DD2var32 (DBLα1.7-CIDRα1.4-DBLβ1), alone and in complex with EPCR, also revealed the formation of a 1:1 PfEMP1:EPCR complex (Table S4). In addition, molecular envelopes showed a predominantly elongated architecture for these three domains (Figure 2A). This architecture did not alter in the presence of EPCR, but instead a single additional protein density was evident, attached to the central CIDRα1 domain (Figure 2A), supporting the notion of a modular arrangement for the PfEMP1 protein with a single EPCR binding site on the CIDRα1 domain.

The CIDRα1:EPCR complexes were next subjected to crystallization trials. Crystals of the HB3var03 CIDRα1.4:EPCR complex formed and diffracted to 2.65Å resolution. The structure was determined by molecular replacement using the structure of EPCR (PDB 1L8J) as a search model, followed by iterative model building and refinement (Figures 2B and S3; Table S5). The structure was consistent with an envelope obtained from solution small-angle X-ray scattering (Figures S2D–S2F), and the two copies of HB3var03 CIDRα1 in the asymmetric unit aligned with a root-mean-square deviation (rmsd) of just 0.09Å, showing them to be extremely similar.

A second complex, containing IT4var07 CIDRα1.4:EPCR complex also crystallized, and crystals diffracted to 2.9Å resolution. Despite a sequence identity of 78.5% compared to the HB3var03 CIDRα1 domain, this complex crystallized in a different space group and with different crystal packing. The structure was determined using HB3var03 CIDRα1 and EPCR as separate search models in molecular replacement. HB3var03 and IT4var07 CIDRα1 are extremely similar (rmsd = ∼0.3Å), but despite differences in space groups and crystal packing, both CIDRα1 domains bind to EPCR using the equivalent surface (Figure 2C).

**Figure 3:**
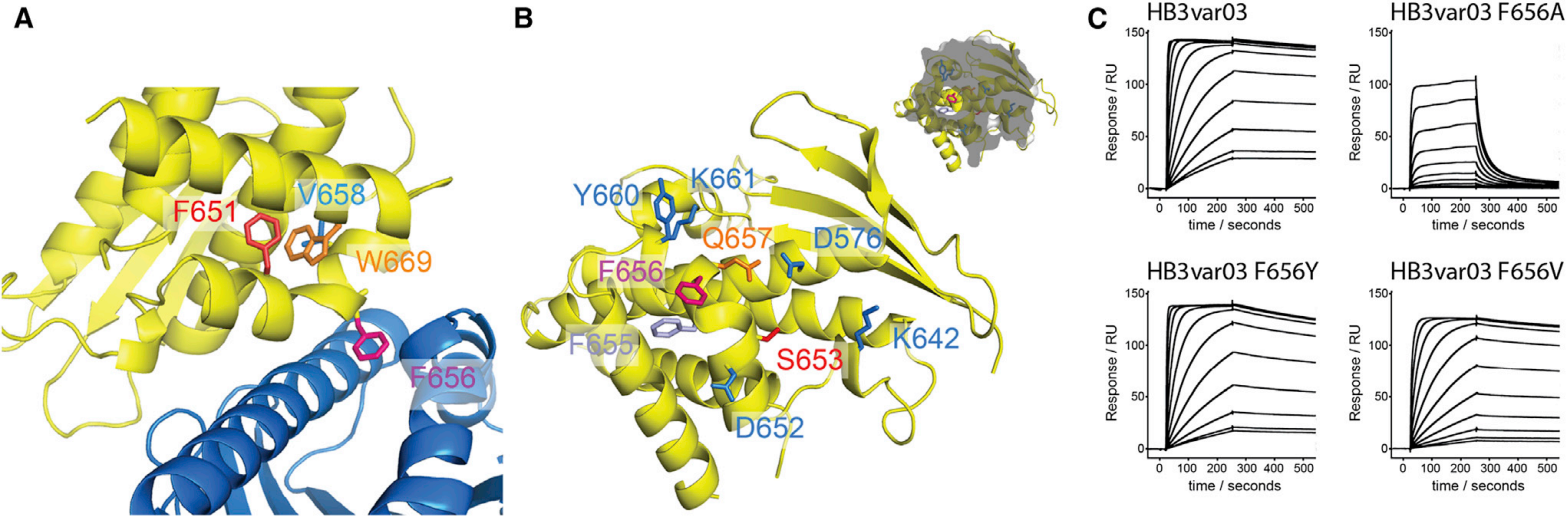
The Architecture of the EPCR Binding Site (A) A close up of the EPCR binding site with HB3var03 CIDRα1 (yellow) and EPCR (blue). Three residues (F651, V658, and W669) that lie beneath the kinked helix are labeled. This kink causes F656 to protrude and interact with the hydrophobic groove of EPCR. (B) Structure of the EPCR-binding surface of HB3var03 CIDRα1. Residues shown as sticks directly interact with EPCR. The inset shows a view of the HB3var03 CIDRα1 domain in the same orientation with a gray cross-section of EPCR chosen to show F656 protruding into the hydrophobic groove of EPCR. (C) SPR data showing binding of HB3var03 and its F656A, F656V, and F656Y mutants to EPCR. See also Figures S3 and S4.

The two CIDRα1 domains are built around a long three-helical core bundle. On one side of this bundle lies a four-stranded β sheet. On the opposite side, between the second and third core helices, an insertion folds into a kinked α helix and a long α helix that lie approximately perpendicular to the core bundle, stabilized by residues F651, V658, and W669 and forming the majority of the EPCR-binding surface (Figure 3A). The α-helical core of the CIDRα1 domain, and the EPCR-binding surface, are well ordered and well defined in the crystal structure, with B factors of 20–40 (Figures S2I and S2J). However, away from the binding surface, the domain is decorated with a variety of loops, some of which are not observed in the electron density, while others are characterized by high B factors, suggesting flexibility. Comparison with the two existing CIDR domain structures shows the CIDRα1 domains to be more similar to CIDRγ from var0 (Vigan-Womas et al., 2012) (Figure 2D) than to CIDRα2 from CD36-binding MC179 (Klein et al., 2008), with the most significant structural differences in the EPCR-binding region. This region is part of a homology block (HB121) that is unique to CIDRα1 domains (Rask et al., 2010).

**Figure 4:**
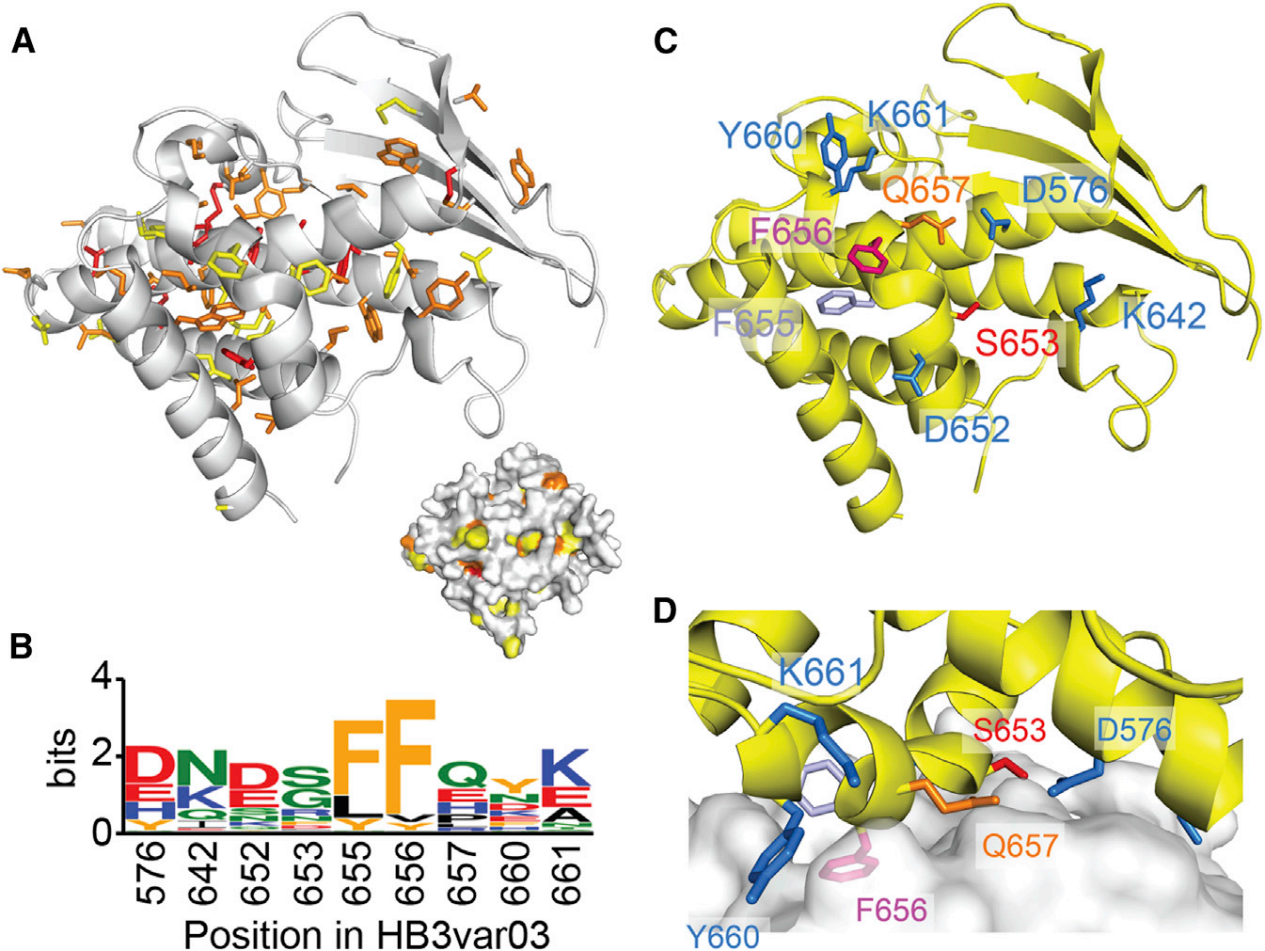
Diversity and Conservation in the CIDRα1 Domains (A) The 14 completely conserved residues in CIDRα1 domains, shown as red sticks on the HB3var03 CIDRα1 structure. Residues with a property entropy score of less than 0.2 (but not totally conserved) are orange, and those with scores of 0.2–0.3 are yellow. The inset shows a surface representation in the same orientation and colors, showing that conserved residues cluster in the domain center. (B) A sequence logo showing variation in CIDRα1 residues that directly contact EPCR. (C and D) Structure of the EPCR-binding surface of the HB3var03 CIDRα1 domain. Residues shown as sticks make direct interactions with EPCR. See also Figure S5.

The HB3var03 CIDRα1:EPCR interface has a surface area of 978Å^2^ and surrounds the kinked helix (Figure 3A). At its center is a small hydrophobic patch containing F656 and F655, which interact with a hydrophobic patch on EPCR. In particular, F656 is positioned at the bend in the kinked α helix, where it protrudes from the domain surface, allowing it to insert into the hydrophobic groove of EPCR (Figures 2B and 3A). This patch lies within a larger surface that is complementary in shape to EPCR and contains a series of amino acids (D576, K642, D652, S653, Q657, Y660, and K661) that make hydrogen bonds to EPCR side chains (Figures 3B and 4).

Both the hydrophobic contacts and the hydrogen bonds play an important role in the interaction. ITC measurements show that binding is driven by a negative enthalpy change (Figure S2A), most likely due to formation of hydrogen bonds. However, insertion of F656 into the hydrophobic groove of EPCR is also important for complex stability. Mutation of F656 to hydrophobic residues, tyrosine or valine, reduced the affinity by only ∼4-fold. However, the F656A mutation, which removes the majority of this interaction, had a 35-fold effect on the affinity and significantly increased the off rate, with a 100-fold change in the dissociation rate constant (Figures 3C and S4; Table S6). Therefore, the insertion of F656 into the hydrophobic groove of EPCR increases the lifetime of the complex. In combination with hydrogen bond formation, this leads to a high affinity and stable interaction, as required to hold infected erythrocytes in place against the buffeting of blood flow.

### PfEMP1 and the Natural Ligand Protein C Share an Overlapping Binding Site on EPCR

**Figure 5:**
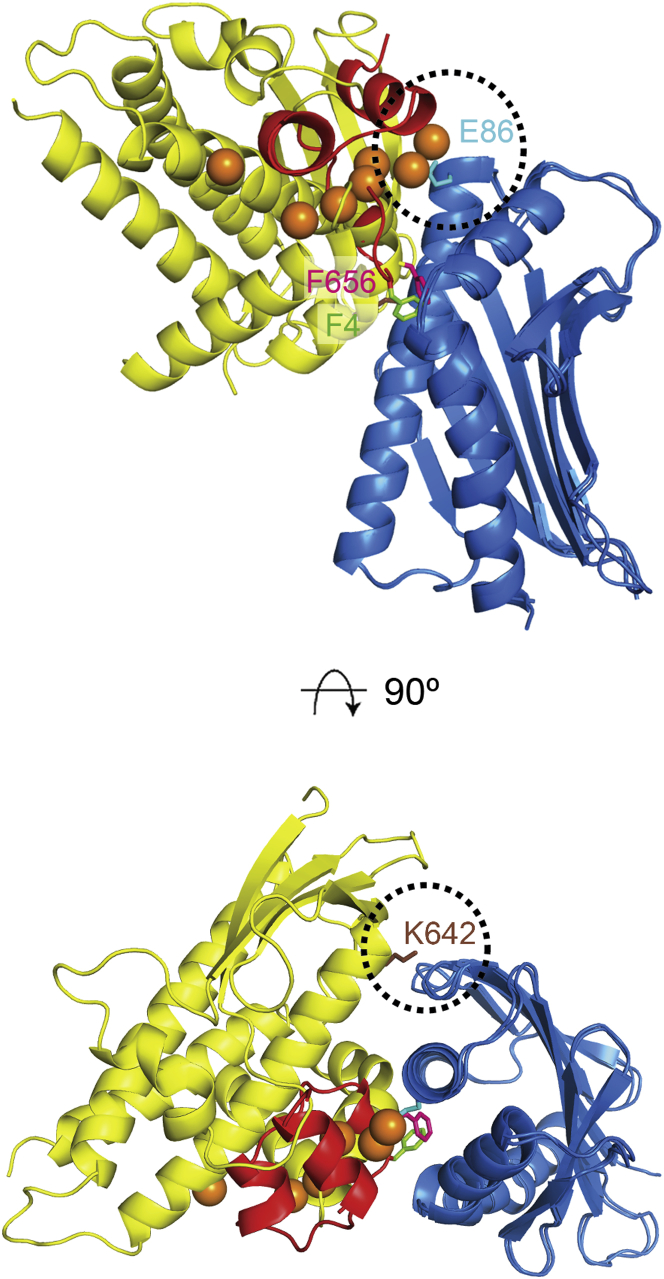
The CIDRα1 Domains Overlap the Protein C Binding Site on EPCR Structure of a complex of the CIDRα1 domain of HB3var03 (yellow) bound to EPCR (blue), overlaid with that of the Gla domain of activated protein C (red) bound to EPCR. Calcium ions in the Gla domain are shown as orange spheres. Residues F4 of protein C (green) and F656 of HB3var03 CIDRα1 (pink) bind the same pocket of EPCR. Residue E86 of EPCR (cyan) interacts with the calcium ions of protein C, forming a binding surface largely unused by CIDRα1. Loops of CIDRα1 domain, including K642 in HB3var03 (brown), interact with loops from EPCR not contacted by protein C.

We previously demonstrated that preincubation of EPCR with CIDRα1 domains prevents the interaction with its natural ligand, protein C, suggesting that infected erythrocytes might interfere with EPCR-mediated signaling in severe malaria (Turner et al., 2013). Indeed, comparison of the structures of the CIDRα1:EPCR complexes with that of EPCR bound to the Gla domain of activated protein C (Oganesyan et al., 2002) reveals significant overlap, with both protein C and CIDRα1 domains interacting with the same region of the hydrophobic groove of EPCR (Figure 5).

Most of the interactions between protein C and EPCR (718Å^2^ of the total 771Å^2^ interaction surface) are mediated through a hydrophobic loop from protein C that positions residues F4 and L5 to bind into the hydrophobic groove of EPCR. In particular, F4 binds to the same site as F656 from HB3var03 CIDRα1 (Figure 5). The overlapping binding sites of protein C and CIDRα1 domains will likely cause sequestered infected erythrocytes to inhibit the binding of activated protein C. It is conceivable that evolution has driven PfEMP1 to interact with this functionally important ligand-binding site of EPCR to reduce the likelihood of host mutations occurring in the interface that would disrupt PfEMP1 binding.

Although the binding sites overlap, there are also significant differences. The CIDRα1-binding site is larger than that of protein C, extending to make contacts with two additional EPCR loops (residues 22–25 and 44–47) through K642 of the HB3var03 CIDRα1 domain. In contrast, protein C makes strong electrostatic interactions through an associated calcium ion with E86 of EPCR, a residue that plays little role in the interaction with CIDRα1 domains. Therefore, although protein C and CIDRα1 domains overlap, there are differences in their binding sites, which may be exploitable in the development of therapeutic EPCR variants or compounds that interact with just protein C or PfEMP1.

### Structural Conservation and Surface Diversity of the EPCR-Binding CIDRα1 Domains

Having identified residues and structural features used by the HB3var03 and IT4var07 CIDRα1 domains to bind to EPCR, we next assessed the degree of conservation of these features in 737 CIDRα sequences from the EPCR-binding CIDRα1 subclasses. As predicted, the 14 totally conserved residues were all located in the core of the domain, with aromatic residues packing together at the interfaces between the helices and cysteines forming disulphide bonds to stabilize the structure (Figure 4A).

With so little absolute sequence conservation, we next assessed the degree to which residues varied while retaining their chemical property. The aligned sequences were interrogated at each amino acid position, using the Shannon entropy of physiochemical property method (Capra and Singh, 2007; Mirny and Shakhnovich, 1999), and scores were plotted onto the HB3var03 CIDRα1 structure. Side chains showing significant conservation of surface property (a score of <0.3) are also mostly internal and are likely to contribute to the fold (Figure 4A). These include aromatic residues (F651, V658, and W669 in HB3var03) that lie between the core bundle and binding helix and contribute to the formation of the kink and arrangement of residues critical for binding (Figure 3A). Indeed, single point mutations of these residues, designed to disturb these structural features, reduced EPCR binding affinity by up to 100-fold (Figure S4; Table S6). Therefore, as seen in the DBL domains (Higgins and Carrington, 2014), most conserved residues in CIDRα1 are internal, most likely stabilizing the domain architecture and correctly positioning surface residues for ligand binding.

In contrast, surfaces of CIDRα1 domains, which are under immune selection pressure to diversify, vary significantly. This extends even to residues that make direct contacts with EPCR. Here, the most significant conservation is at positions occupied by F655 and F656 in HB3var03 (Figures 4B–4D and S5). While phenylalanine is most common in both positions, other aromatic or hydrophobic side chains, such as tyrosine, leucine, or valine, are also observed. These relatively conservative changes retain the hydrophobic nature of the protrusion, maintaining its capacity to insert into the hydrophobic groove of EPCR. Indeed, the F655L, F655Y, F656Y, and F656V mutants of HB3var03 all bind to EPCR with affinities of less than 5 nM (Figure S4; Table S6). The other residues that interact with EPCR, through hydrogen bonding interactions, are more divergent. However, at each of these positions, the most common substitution is with another amino acid that can form hydrogen bonds (Figure 4).

Interestingly, the CIDRα1.2 and CIDRα1.3 subclasses, shown not to bind EPCR, contain residues in these nine positions consistent with binding. However, they also share a lysine at the position equivalent to Q657 in HB3var03, a change that is not observed in other subclasses and that places a positive charge next to an arginine in EPCR, most likely leading to repulsion. Indeed, the Q657K mutation of HB3var03 leads to an ∼200-fold reduction in EPCR binding affinity and a dramatic increase in the off rates (Figure S4; Table S6), suggesting that this change makes a significant contribution to the lack of EPCR binding by these subclasses.

Therefore, the EPCR binding surface shows significant sequence variability but retains its structure through conservation of core residues. It also retains the essential chemical nature of its surface residues through retention of a hydrophobic protrusion and a surrounding network of hydrogen bond donors and acceptors.

### Patient Sera Contain Antibodies that Disrupt EPCR Binding with Cross-Inhibitory Potential

The high surface sequence variability of CIDRα1s, driven by the selection pressure to avoid immune detection, will reduce the likelihood of acquisition of cross-reactive antibodies that prevent EPCR binding and erythrocyte sequestration. However, despite this diversity, natural immunity to severe malaria is acquired after only one or two severe infections and involves IgG that target PfEMP1 (Bull et al., 1998; Salanti et al., 2004; Lusingu et al., 2006; Cham et al., 2009; Gupta et al., 1999; Nielsen et al., 2002; Gonçalves et al., 2014). We therefore investigated whether individuals (aged 4–15 years) from a malaria-endemic region of Tanzania had acquired antibodies that bind recombinant HB3var03 CIDRα1.4 and IT4var20 CIDRα1.1 domains and to what extent these antibodies can prevent EPCR binding by a diverse set of CIDRα1 domains. HB3var03 CIDRα1.4 and IT4var20 CIDRα1.1 domains share only four out of nine residues that contact EPCR and were selected to represent the two major divergent groups of CIDRα1 domains (see Figure 1A), the CIDRα1.2–CIDRα1.7 domains and the CIDRα1.1/CIDRα1.8 domains, encoded by *var* genes controlled by UPSA and UPSB promoters, respectively (Lavstsen et al., 2003; Sander et al., 2014). We found that a large fraction of tested individuals had acquired IgG capable of inhibiting EPCR binding by HB3var03 CIDRα1.4 or IT4var20 CIDRα1.1 (41/45 and 51/76, respectively).

**Figure 6:**
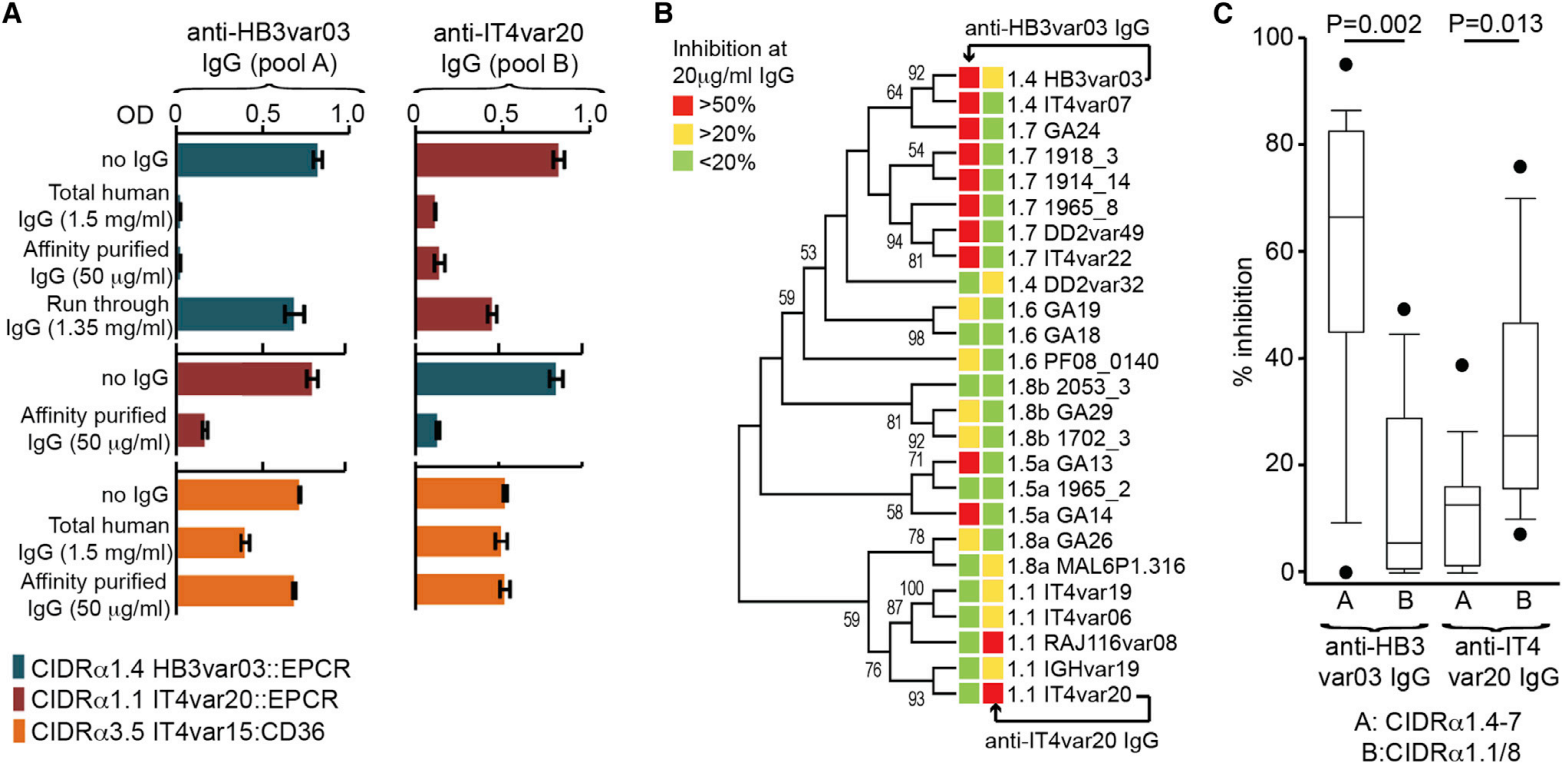
Human Sera Contain Antibodies that Block the CIDRα1:EPCR Interaction (A) Human IgG preparations from Tanzanian individuals inhibit ELISA binding of HB3var03 CIDRα1.4 or IT4var20 CIDRα1.1 to EPCR, but not the binding of CIDRα3.5 to CD36. Antibodies tested included total human IgG reactive to the CIDRα1 domain under study (Total human IgG), IgG affinity purified using a peptide covering the EPCR-binding site of the CIDRα1 domain under study (Affinity purified IgG), and IgG that did not bind to this affinity column (Run through IgG). This was done for a UPSA PfEMP1 (HB3var03, pool A) and a UPSB PfEMP1 (IT4var20, pool B). (B) Inhibition of binding of 25 CIDRα1 domains to EPCR by two peptide affinity-purified human IgG preparations (anti-HB3var03 and anti-IT4var20 IgG). The sequence similarity of the region corresponding to the peptide sequence of the 25 domains is shown by the maximum likelihood tree. The level of binding inhibition of each CIDRα1 domain to EPCR by the IgG preparations is shown by color-coded boxes plotted on the tree. (C) A summary of the percentage of EPCR binding inhibition (median and 10^th^, 25^th^, 75^th^, and 90^th^ percentiles) of each affinity purified IgG preparation on CIDRα1 domains belonging to UPSA or UPSB (rank-sum p values) shows the greatest cross-inhibition of UPSA domains by UPSA-purified sera and of UPSB domains by UPSB-purified sera. See also Figure S6.

Next, to assess the cross-inhibitory potential of these antibodies, plasma from individuals with IgG reactive to HB3var03 were pooled, and IgG was affinity purified using a synthetic peptide containing the EPCR-binding region of HB3var03 (generating IgG pool A). The same procedure was used for IT4var20 reactive sera using a binding-site peptide from IT4var20 (IgG pool B). Despite being purified on different peptides, both IgG preparations reacted in ELISA with both HB3var03 and IT4var20 CIDRα1 domains, but not with CD36 binding CIDRα3 control domains (Figure S6). In addition, at 50 μg/ml IgG concentration, both IgG pool A and IgG pool B showed almost complete inhibition of the binding of both IT4var20 and HB3var03 CIDRα1 domains to EPCR (Figure 6A). We also tested the ability of pool A IgG to recognize activated protein C and saw no cross reactivity, indicating that these IgG will not affect the EPCR:APC interactions (Figure S6B). Indeed, although CIDRα1 domains and APC share an overlapping binding site on EPCR (Figure 5), they are structurally very different, making it unlikely that antibodies raised against CIDRα1 domains will cross-react with APC.

To assess the degree to which these IgG cross-inhibit EPCR binding, they were tested against a set of 25 EPCR binding CIDRα1 domains, selected to represent the breadth of sequence diversity. At a lower concentration of 20 μg/ml, pool A IgG reduced EPCR binding by most of the UPSA CDIRα1 domains tested and had a significantly lower effect on CIDRα1 variants from UPSB. Conversely, pool B IgG reduced EPCR binding by many of the UPSB CIDRα1.1 domains and showed statistically significant lower reduction of binding by UPSA domains (Figures 6B and 6C).

**Figure 7:**
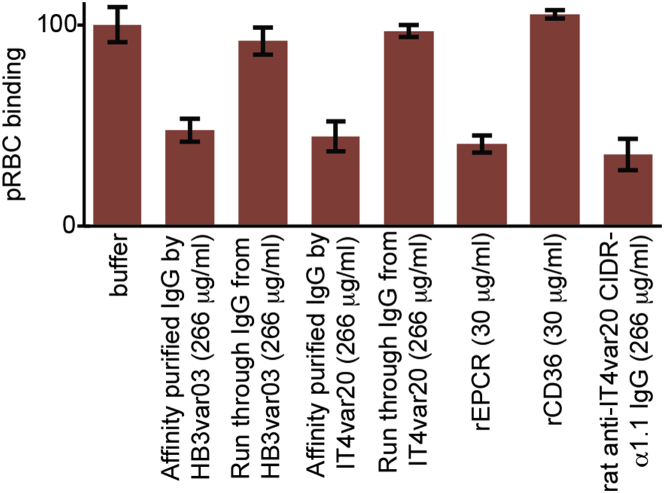
Human Sera Contain Antibodies that Block Infected Erythrocytes from Binding to EPCR Binding inhibition of parasite-infected erythrocytes expressing native IT4var20 (pRBC) to HBMECs by IgG preparations (Affinity purified IgG by either HB3var03 CIDRα1 or IT4var20 CIDRα1 peptides), control IgG (Run through IgG from either HB3var03 or IT4var20), soluble recombinant EPCR and CD36, and IgG from a rat immunized with IT4var20 CIDRα1.

Finally, we tested whether the purified IgG could also block endothelial cell binding by parasite-infected erythrocytes expressing IT4var20, a PfEMP1 shown to bind only to EPCR (Turner et al., 2013). Both pool A and pool B IgG inhibited this binding to the same extent as soluble EPCR or IgG raised against the CIDRα1.1 domain of IT4var20 (Figure 7). These data show that individuals living in malaria-endemic areas acquire functional antibodies that target the ligand-binding region of CIDRα1 through natural infection. These antibodies have the capacity to block both EPCR binding of CIDRα1 domains and endothelial cell binding by parasite-infected erythrocytes. They also show some cross-inhibitory potential, with IgG affinity purified on the EPCR-binding region of one CIDRα1 domain able to reduce EPCR binding by other, diverse CIDRα1 domains.

## Discussion

Parasites frequently express surface protein families that lie at the interface between pathogen and host, experiencing the selection pressure to diversify to avoid immune detection while maintaining conserved features required for their function in host-parasite interactions. The roles of these protein families vary significantly. Some, such as the Trypanosome variant surface glycoproteins (VSGs), form a structural coat, protecting the parasite surface underneath, but have no known human ligands (Schwede and Carrington, 2010). In contrast, the PfEMP1 proteins from *Plasmodium falciparum* have a more complex function with the requirement to retain host receptor binding (Smith et al., 2013). This imposes additional constraints on protein family evolution, raising questions about the degree to which such a protein family can diversify while retaining host interaction capability. It also raises questions for those engaged in vaccine development about whether features found on the protein surface are sufficiently conserved to allow the immunogen-mediated induction of a broadly neutralizing immune response.

In this study we provide the structure of a module from a divergent parasite-expressed surface protein family in complex with its host ligand. This reveals the structural features that the CIDRα1 domains from the PfEMP1 proteins have evolved in order to interact with EPCR. In particular, we see the acquisition of a loop between the second and core third helices and the folding of this loop into a platform on which EPCR docks. We see the presence of a kinked helix, promoting the protrusion of a hydrophobic residue into the hydrophobic groove of EPCR, mimicking F4 from the natural ligand of EPCR, protein C. We also see a surface decorated with hydrogen bond donors and acceptors that makes further interactions with EPCR and stabilizes the complex.

This structure provides us with the framework to understand analysis of 737 sequences of EPCR-binding CIDRα1 domains. It reveals that most conserved residues are found in the interior of the domain, with conserved disulphide bonds stabilizing the fold, and conserved aromatic residues facilitating helical packing, as in the related DBL domains (Higgins and Carrington, 2014). Conserved aromatic residues also stabilize the kinked architecture of the EPCR-binding loop, making it extremely likely that this is conserved across all CIDRα1 variants.

In contrast, surface sequence conservation is extremely low. Even residues that directly contact EPCR exhibit significant diversity. However, the potential for bond formation is largely retained, with the hydrophobic protrusion remaining hydrophobic in the large majority of CIDRα1 domains, and residues with hydrogen bonding potential are largely replaced with other hydrogen bond donors and acceptors. Therefore the CIDRα1 domains appear to retain a conserved architecture but with extensive surface divergence. However, the EPCR-binding surface retains sufficient chemical similarity to allow retention of the capacity to form a stable complex with EPCR.

This study focuses on the interaction of PfEMP1 with one host receptor. However, we expect other such surface protein families to follow similar principles. As we see here for EPCR, it has been challenging, or impossible, to find conserved residues on PfEMP1 domains that interact with other host receptors, such as ICAM-1 or CD36, by sequence analysis alone (Robinson et al., 2003; Howell et al., 2008). With immune pressure driving surface variation to extremes, it is likely that retention of conserved domain architecture and surface interaction potential, rather than conservation of amino acid identity, is a general feature of PfEMP1 and other divergent parasite protein families. Future structural biology studies will be required to confirm this and to identify the molecular determinants required to bind to other host receptors.

Such extensive surface variation, even across the ligand-binding surface, raises questions about whether it will be possible to design immunogens that induce immunoglobulins that can block EPCR binding by all PfEMP1. Such an immunogen would have significant value in the prevention of severe malaria. Our studies of IgG affinity purified from individuals from Tanzania provide some hope, as they reveal that natural infection has led to the acquisition of IgG with the capacity to reduce EPCR binding by diverse CIDRα1 domains and endothelial cell binding by the FCR3 IT4var20 parasite line.

How cross-inhibitory these responses can become, to what extent they protect against severe pediatric malaria, and whether improved immunogens can be developed that allow their induction remain questions for the future. In addition, it is currently unknown what fraction of EPCR-binding PfEMP1 contains domains that interact with additional endothelial receptors or whether preventing EPCR occupancy is sufficient alone to ameliorate disease symptoms. However, the retention of bonding potential across the EPCR-binding surface of the CIDRα1 domains does suggest that it might be possible to raise IgG, which present a chemical surface that mimics features of EPCR, containing the ability to bind to and block the EPCR-binding surfaces of all CIDRα1s. Future studies will need to determine whether such IgG can be generated, assessing whether the necessity for PfEMP1 to retain conserved structural features to allow EPCR binding can provide a route to target the parasite and contribute to the prevention of severe malaria.

## Experimental Procedures

More detailed methods are in the Supplemental Experimental Procedures.

### Protein Expression and Purification

CIDRα1 domains for binding studies were expressed in baculovirus-infected High Five cells and purified by metal affinity chromatography. For crystallization, CIDRα1 domains were expressed in *E. coli* in inclusion bodies and were refolded on a Ni-NTA column, followed by size exclusion chromatography.

EPCR was expressed in a stable *Drosophila* S2 cell line (ExpreS^2^ion Biotechnologies). Culture media was buffer exchanged and EPCR purified by Ni-NTA affinity chromatography and size exclusion gel chromatography. Protein for crystallography was deglycosylated by treatment with endoglycosidase H_f_ and endoglycosidase F3, and tags were removed using TEV protease.

### Crystal Structure Determination

HB3var03 CIDRα1:EPCR and IT4var07 CIDRα1:EPCR complexes were purified by size exclusion chromatography. Crystals were grown using sitting-drop vapor diffusion. HB3var03 CIDRα1-EPCR crystals grew with a reservoir solution of 0.2 M NaNO_3_, 0.1 M BTP (pH 8.5), 20% PEG 3350 and were cryo-cooled in well solution containing 25% ethylene glycol. IT4var07 CIDRα1-EPCR crystals grew with a reservoir solution of 0.2 M NaNO_3_, 0.1 M BTP (pH 7.5), 20% PEG 3350 and were cryo-cooled in well solution with 25% MPD.

Data were collected on beamlines I02 and I04 (Diamond Light Source), indexed, refined using iMosflm (Leslie and Powell, 2007), and scaled using SCALA (Collaborative Computational Project, Number 4, 1994). Molecular replacement using Phaser-MR (Collaborative Computational Project, Number 4, 1994) found two copies of EPCR (PDB ID: 1L8J) in the asymmetric unit of HB3var03 CIDRα1:EPCR crystals. CIDRα1 domain models were built using a cycle of refinement, in Refmac (Collaborative Computational Project, Number 4, 1994) and autobuster (Bricogne et al., 2011), and model building was done in Coot (Emsley et al., 2010). The IT4var07 CIDRα1:EPCR structure was determined using Phaser-MR with the HB3var03 CIDRα1:EPCR complex as a search model and refined as above.

### Surface Plasmon Resonance

SPR experiments were carried out in a Biacore T200 instrument (GE Healthcare). EPCR was biotinylated by incubation with BirA and coupled to a biotin capture chip (GE Healthcare) to 150 RU. Binding partners were injected for 240 s with a dissociation time of 300 s before chip regeneration. Specific responses were calculated by subtracting the response from a surface lacking EPCR. The kinetic sensorgrams were fitted to a 1:1 interaction model to allow calculation of kinetic rate constants and dissociation constant.

### Isothermal Titration Calorimetry

ITC measurements were performed at 25°C in a MicroCal iTC200 System (GE Healthcare) with 60 μl of EPCR at 36 μM titrated into a cell containing 300 μl of CIDRα1 HB3var03 at 2.8 μM. Data were integrated and fit by nonlinear least-squares fitting using Origin ITC Software (GE Healthcare).

### Small-Angle X-Ray Scattering

SAXS data were collected on beamline P12 at DESY (Hamburg, Germany) and processed using the ATSAS processing suite. The resulting model was converted into an envelope using Situs (Wriggers, 2010) before model docking using Sculptor (Birmanns et al., 2011).

### Sequence Analysis

CIDRα1 sequences were extracted from assemblies of Illumina whole-genome sequencing data available through the MalariaGEN community and assembled with Velvet (Zerbino, 2010). The Shannon property entropy was calculated on the basis of physiochemical property groupings.

### Human IgG Antibody Purification

Plasma samples collected in 2005 during a cross-sectional malaria survey in an area of high malaria transmission (Tanzania) were screened for ability to inhibit the EPCR binding of HB3var03 CIDRα1.4 or IT4var20 CIDRα1.1 in ELISA. Inhibitory plasma were screened by ELISA for reactivity to peptides covering the EPCR binding region of HB3var03 CIDRα1.4 or IT4var20 CIDRα1.1. IgG preparations were purified by affinity to HB3var03 or IT4var20 peptides, and their binding properties were analyzed by ELISA.

### Parasite Assays

Human brain microvascular endothelial cells (HBMECs) were grown to a monolayer. Ring-stage infected erythrocytes from the FCR3 IT4VAR20 parasite line were tritiated, and 24 hr later, radioactively labeled late trophozoite and schizont stages were purified and incubated with HBMECs for 1 hr at 37°C. Unbound infected erythrocytes were removed with a washing robot (Biomek 2000, Beckman Coulter) and radioactivity measured on a Topcount NXT (PerkinElmer). Adhesion was calculated as the percentage of bound radioactively labeled infected erythrocytes out of the total amount of radioactively labeled infected erythrocytes added per well.

## Author Contributions

C.K.Y.L. purified and crystallized proteins, collected and analyzed SAXS data, and performed SPR, ITC, MALLS, and AUC. C.K.Y.L. and M.K.H. prepared crystals, collected data, and solved structures. E.D.L. collected data. L.T. produced protein and prepared human IgG and performed ELISA studies. J.L. contributed sera. J.S.J., B.P., and T.L. performed bioinformatic analysis. C.W.W. and J.E.V.P. performed parasite binding experiments. C.K.Y.L., L.T., T.T., T.L., and M.K.H. devised the study and wrote the manuscript.

## Accession Numbers

Coordinates and experimental data are deposited in the PDB with accession numbers 4V3D and 4V3E.

## Supplemental Information

Document S1. Supplemental Experimental Procedures, Figures S1–S6, and Tables S1–S6

Document S2. Article plus Supplemental Information
