# Supplementary material for: Structural Conservation Despite Huge Sequence Diversity Allows EPCR Binding by the PfEMP1 Family Implicated in Severe Childhood Malaria
Source: Cell Host Microbe. 2015 Jan 14;17(1):118–29. doi: 10.1016/j.chom.2014.11.007 (PMC4297295; doi:10.1016/j.chom.2014.11.007)
Supplement: Document S1. Supplemental Experimental Procedures, Figures S1–S6, and Tables S1–S6 [file mmc1.pdf]

Cell Host & Microbe, Volume *17*

Supplemental Information

Structural Conservation Despite Huge Sequence Diversity Allows EPCR Binding by the PfEMP1 Family Implicated in Severe Childhood Malaria

Clinton K.Y. Lau, Louise Turner, Jakob S. Jespersen, Edward D. Lowe, Bent Petersen, Christian W. Wang, Jens E.V. Petersen, John Lusingu, Thor G. Theander, Thomas Lavstsen, and Matthew K. Higgins

Supplemental experimental procedures

Expression and purification of CIDRα1 domains

Domain sequences originate from *Plasmodium falciparum* reference genomes (Rask et al., 2010) or sequenced Tanzanian patient isolates (four digit names from Lavstsen et al., 2012 and unpublished; ERSxxxxxx names from Miotto et al., 2013). In addition to previously published CIDRα1 domains (Turner et al., 2013), new CIDRα1 DNA sequences were optimised for expression in *Trichoplusia ni* cells using the software GeneOptimizer. They were synthesised by Geneart (Regensburg, Germany) after modification to include C-terminal V5 and His tags and were subcloned into the baculovirus expression vector pAcGP67-A (BD Biosciences). FlashBac DNA (Oxford Expression Technologies, OE-100150) was co-transfected with the recombinant vectors into Sf9 insect cells for the generation of recombinant virus particles.

High-Five insect cells grown in 600 ml of serum-free media (10486; GIBCO) were infected with 18 ml of the second amplification of the recombinant virus particles. Two days later the cells were centrifuged (8000g, 4 °C, 10 min), and the supernatant was filtered using two 10-kDa NMWC PES membranes (0.45 µm) (56-4112-04; GE Healthcare) with a total surface area of 200 cm^2. The supernatant was then concentrated to 30 ml and diafiltrated using an ÄKTA cross-flow (GE Healthcare) into buffer A (20 mM Tris (pH 9.0) and 500 mM NaCl). Retentate was recovered from the system and filtered (0.2 µm), yielding a final volume of 40 ml. Before loading onto a 1 ml HisSelect column (H8286; Sigma-Aldrich), 150 µl of 1 M imidazole (pH 7.4; Sigma-Aldrich) was added to the sample, giving a final imidazole concentration of 15 µM. The bound protein was eluted with buffer A+200 mM imidazole.

For crystallisation, CIDRα1 domains from HB3var03 and IT4var07 were expressed in *E. coli*. Domain boundaries were selected using a structure-based alignment against pdb 2YK0 (Vigan-Womas et al., 2012) using FUGUE (Shi et al., 2001). Genes were amplified from HB3 or IT4 strain *P. falciparum* genomic DNA respectively and were cloned into the pEt15b vector. This allowed expression of the protein with an N-terminal hexa-histidine tag and a TEV cleavage site using *Escherichia coli* (BL-21 strain). Transformed *E. coli* were grown up to an optical density at 600 nm of 1.0 and expression was induced by the addition of IPTG to a final concentration of 1 mM. Cells were harvested after 3 h at 27°C. The CIDRα1 domains expressed in the form of inclusion bodies, which were unfolded by incubation at room temperature in 6 M guanidine-hydrochloride, 20 mM Tris pH 8, 300 mM NaCl, 15 mM imidazole for 15 h. Refolding was achieved by gradual buffer exchange into 20 mM Tris pH 8, 300 mM NaCl, 15 mM imidazole in the presence of a glutathione redox buffer (3 mM reduced glutathione, 0.3 mM oxidised glutathione) while the protein was bound to a Ni-NTA column. Refolded protein was eluted and further purified by size exclusion gel chromatography (HiLoad Superdex 75 16/60, GE Healthcare) into 20 mM HEPES pH 7.5, 150 mM NaCl.

Single site mutants were generated from the wild type HB3var03 CIDRα1 construct as described by the Quikchange mutagenesis method (Stratagene) and

plasmid sequences were verified. Proteins from these constructs were expressed and purified in *E. coli* as above.

Recombinant EPCR expression and purification

A codon-optimised synthetic gene was designed for EPCR (Syngene), using domain boundaries obtained from the crystal structure of pdb 1L8J (Oganesyan et al., 2002) and was produced by Geneart. This gene was cloned into the pExpreS^2 vector, in frame with a BIP leader sequence and an N-terminal extension containing a BAP tag, an N-terminal hexa-histidine tag, and a TEV cleavage site. The vector was then transfected into *Drosophila* S2 cells using ExpreS^2 Insect TRx5 liposome transfection reagent (Expres^{2}ion Biotechnologies). Stably transfected cells were selected using zeocin (Invitrogen, Thermo Fisher Scientific). Culture media containing EPCR was buffer exchanged into 20 mM Tris pH 8, 500 mM NaCl and protein was purified by Ni-NTA affinity chromatography and size exclusion gel chromatography (HiLoad Superdex 75 16/60, GE Healthcare) using 20 mM HEPES pH 7.5, 150 mM NaCl.

Protein for crystallography was deglycosylated by treatment with endoglycosidase H_f (Sigma) and endoglycosidase F3 at enzyme:protein ratios of 1:50 in 50 mM MES pH 6.5 for 15 h. N-terminal tags were cleaved using TEV protease at an enzyme:protein ratio of 1:50 in PBS (Melford) with 3 mM reduced glutathione, 0.3 mM oxidised glutathione for 15 h at 25°C.

CIDRα1-EPCR complex crystallisation

HB3var03 and IT4var07 CIDRα1s were mixed separately with EPCR at 1:1 molar ratios and complexes were obtained by size exclusion chromatography on a Superdex 75 HiLoad 16/60 column (GE Healthcare). Fractions containing complexes were concentrated up to 10.7 mg/ml (HB3var03) and 8.1 mg/ml (IT4var07). Crystals were grown using the sitting-drop vapour-diffusion method in 96 well plates. Initial crystals were obtained through a broad screen with droplets containing 100 nl of protein solution mixed with 100 nl of well solution. Optimisation screens were performed including seed stocks obtained from initial crystals, with each droplet contained 100 nl of protein solution, 50 nl of seed solution and 50 nl of well solution and 50 nl of additive solution (Silver Bullets, Hampton Research).

HB3var03 CIDRα1-EPCR crystals grew as needle clusters with a reservoir solution of 0.2 M $NaNO_3$, 0.1 M BTP pH 8.5, 20% PEG 3350. Needle clusters were transferred into a drop of well solution containing 25% ethylene glycol and single needles detached. These needles were then cryo-cooled in liquid nitrogen for storage and data collection.

IT4var07 CIDRα1-EPCR crystals grew as hexagonal prisms with a reservoir solution of 0.2 M $NaNO_3$, 0.1 M BTP pH 7.5, 20% PEG 3350. MPD was gradually

added to the crystals in a drop of mother liquor to a final concentration of 25%. Crystals were cryo-cooled in liquid nitrogen for storage and data collection.

Data collection and structure determination

Data from crystals containing the HB3var03 CIDRα1-EPCR complex were collected at beamline I04 (Diamond Light Source, UK) using radiation with a wavelength of 0.98Å and a Pilatus 6M-F detector. These data were indexed and refined using iMosflm (Leslie and Powell, 2007), then scaled using SCALA (CCPN4, 1994) to resolution of 2.65 Å. Molecular replacement using Phaser-MR (CCPN4, 1994) found two copies of EPCR (pdb code 1L8J) (Oganesyan et al., 2002) in the asymmetric unit. The models of the CIDRα1 domains were built into the remaining electron density using an iterative cycle of refinement using Refmac (CCPN4, 1994) and autobuster (Bricogne et al., 2011) and manual model building using Coot (Emsley et al., 2010).

Data from crystals containing the IT4var07 CIDRα1-EPCR complex were collected at beamline I02 (Diamond Light Source, UK) using a Pilatus 6M detector. These data were indexed and refined using iMosflm and scaled using SCALA to a resolution of 2.9 Å. Phaser-MR using the structure of the HB3var03 CIDRα1-EPCR complex found one copy in the asymmetric unit. The model of the IT4var07 CIDRα1:EPCR complex was built using iterative cycles of manual model building using Coot and refinement using autobuster.

Surface plasmon resonance

EPCR was coupled to an SPR chip using a biotin attached to the N-terminal BAP tag. This strategy was designed to allow EPCR to be immobilised with an orientation matching that found on the endothelial surface, and to generate a surface that could readily be regenerated. The S2 cell produced EPCR was not biotinylated during expression, so 1 mg EPCR (30 μM) in 20 mM HEPES pH 7.5, 150 mM NaCl was incubated with 20 μg BirA, 0.3 μM biotin, 5 mM ATP, for 15 h at 25°C.

SPR experiments were carried out on a Biacore T200 instrument (GE Healthcare). All experiments were performed in 20 mM HEPES pH 7.5, 150 mM NaCl, 0.005% Tween-20 at 25°C. Two-fold dilution series of each CIDRα1 were prepared for injection over an EPCR-coated chip. For each cycle, biotinylated recombinant EPCR was immobilised on a CAP chip using the Biotin Capture Kit (GE Healthcare) to a total loading of 150 RU. Binding partners were injected for 240 s with a dissociation time of 300 s. The chip was regenerated in between cycles using regeneration solution from the Biotin Capture Kit (GE Healthcare). The specific binding response of the CIDRα1s to EPCR was determined by subtracting the response given by CIDRα1 from a surface to which no EPCR had been coupled. The kinetic sensorgrams were globally fitted to a 1:1 interaction model to allow calculation of the association rate constant, k_a; the dissociation

rate constant, k_d; and the dissociation constant K_d using BIAevaluation software version 1.0 (GE Healthcare).

Isothermal titration calorimetry

ITC measurements were carried out on a MicroCal iTC200 System (GE Healthcare). Samples were dialysed for 15 h into 20 mM HEPES pH 7.5, 150 mM NaCl at 4°C. Experiments were performed at 25°C with 60 µl of EPCR at 50 µM titrated into a cell containing 300 µl of HB3var03 CIDRα1.4 at 5 µM. Concentrations of both components were checked using a BCA assay kit (Merck Millipore). Data were integrated and fit by nonlinear least-squares fitting using Origin ITC Software (GE Healthcare).

Small angle X-ray scattering

SAXS data were collected on beamline P12 at DESY (Hamburg, Germany) at a wavelength of 0.124 nm using a Pilatus 2M pixel X-ray detector. For each protein sample, a series of data sets were collected for a 2-fold dilution series from 4 mg/ml to 0.125 mg/ml, with buffer measurements taken in between each sample measurement. All samples were in 20 mM HEPES pH 7.5, 150 mM NaCl.

Concentration series for each sample, after normalisation and buffer subtraction, were extrapolated to zero to check for concentration dependent aggregation in Primus (Konarev et al., 2003). The radius of gyration, R_g, and zero angle scattering, I(0), were calculated using AutoRg. Data points from the lowest scattering angle (q), as well as at those at higher q with a low signal to noise ratio, were removed, depending on AutoRg results and Kratky plots. Inverse Fourier calculations of I(q) were used to yield P(r) functions, I(0), R_g and the maximum dimension (D_{max}) using GNOM (Svergun, 1992).

Initial bead models for these data were created *ab initio* using DAMMIF (Franke and Svergun, 2009). These were averaged using DAMAVER (Volkov and Svergun, 2003), and refined from the averaged model, based on the original P(r) function, using DAMMIN (Svergun, 1999). The resulting model was converted into an envelope using Situs (Wriggers, 2010), before docking pdb models into the envelope using Sculptor (Birmanns et al., 2011). Chain A from the HB3var03:EPCR structure was used for the CIDRα1 domain model and chain B from the HB3var03:EPCR structure as the EPCR model in the HB3var03:EPCR envelope; the NTSDBL domain from PDB 2YK0 (Vigan-Womas et al., 2012) and the DBL3x domain from PDB 3BQK (Higgins, 2008) were used in addition to the aforementioned domains as models in the DD2var32:EPCR envelope.

Size exclusion chromatography-multiangle laser light scattering

Samples were purified by size exclusion chromatography on a Superdex 75 gel filtration column (analytical grade, GE Healthcare), then analysed using laser light scattering detected at 662 nm wavelength at 8 scattering angles between 20.6° and 149.1° using a Heleos 8 instrument (Wyatt Technology). Molecular weights were calculated using the Zimm equation by ASTRA 6.1 (Wyatt Technology).

Equilibrium analytical ultracentrifugation

Analytical ultracentrifugation was carried out using a Beckman Optima XL-1 analytical ultracentrifuge. Experiments were run until equilibrium at 20°C, with HB3var03 at 8 μM. Absorbance data were taken at a wavelength of 280 nm at equilibrium at 10,000, 12,000, 14,000 and 16,000 rpm analysed the data using sedphat (Vistica et al., 2004), globally calculating the molecular weight modeling a single species of an interacting system.

Sequence analysis

To facilitate analysis of CIDRα1 sequence diversity we expanded our collection of CIDRα1 domains from the previously described 66 sequences, originating mainly from seven whole genome sequenced parasites (Lavstsen et al., 2012; Rask et al., 2010), with domains extracted from assemblies of Illumina whole genome sequencing data from 226 samples collected in both Africa and Asia (Miotto et al., 2013) (Study number ERP000190), available through the MalariaGEN community. All data available from each sample was collectively assembled with Velvet (Zerbino, 2010) using an optimized k-mer value. Assembled contigs were subjected to the Virtual Ribosome open reading frame (ORF) finder (Wernersson, 2006) searching all reading frames, resulting in ~3.4 million ORFs ranging from 21 bp to 30,940 bp, median 258 bp (starting with any codon) and ~850,000 ORFs longer than 750 bp. The 66 different previously classified CIDRα1 sequences (Lavstsen et al., 2012; Rask et al., 2010) were each used to blastp search (Altschul, 1997) the ORFs keeping all hits with an expectation value of $< 10^{-10}$ to extract even very divergent CIDRα1 variants. This resulted in ~1050 hits for each query and a total of 1,894 non-redundant ORF hits. The amino acid sequence of these ORFs were then aligned together with the 66 query sequences using MUSCLE (Edgar, 2004) and the CIDRα1 region excised. Incomplete domain sequences were discarded (domain borders defined in Turner et al, 2013), and the remaining sequences were blastp searched against a library of all known annotated PfEMP1 domains (Rask et al., 2010) (including 649 CIDR domains of all types). Query domains whose best hit was not a CIDRα1 domain were discarded, leaving 831 complete CIDRa1 sequences originating from 198 of the 226 assembled samples. Hand corrected MUSCLE alignments were used to generate sequence logos by WebLogo 3 (Crooks et al., 2004) and

sequence distance trees by MEGA (Tamura et al., 2013). As a measure of the residue-wise conservation/variation of EPCR binding CIDRα1 domains, the Shannon property entropy was calculated on the basis of the physiochemical property groupings defined by Mirny and Shakhnovich (aliphatic [AVLIMC], aromatic [FWYH], polar [STNQ], positive [KR], negative [DE] and special conformation [PG]) and using sequence weighting (Capra and Singh, 2007; Mirny and Shakhnovich, 1999). As shown in Figure 1 and Table S1 these domains grouped into the eight previously defined subtypes with the additional separation of CIDRα1.5, -6 and -8 variants into two, i.e. CIDRα1.5a/b, 1.6a/b and 1.8a/b.

Human IgG antibody purification

Plasma samples collected in 2005 during a cross sectional malaria survey in an area of high malaria transmission (Tanzania) were screened for the ability to inhibit the EPCR binding of HB3var03 CIDRα1.4 or IT4var20 CIDRα1.1 in ELISA. A set of 45 or 76 plasma samples were screened for each protein, respectively. 41 of 45 and 51 of 76 samples inhibited the EPCR binding to OD values <50% of the no plasma control. Inhibitory plasma samples were then screened by ELISA for reactivity to peptides covering the EPCR binding region of HB3var03 CIDRα1.4 or IT4var20 CIDRα1.1, respectively.

The HB3var03 peptide sequence:
LFTNKHDIPKKYYLNINDLFDSFFFQVIYKFNEGEAKWNELKENLKKQIASSKANNGTKDSEA.
The IT4var20 peptide sequence:
VFGNNNRMSYIYYNNLSRVFDSFLFQVMFALDQDEKGKWDQFTEDLKKKFEPSKTNTPTGKSQD

Fifteen of 41 (average donor age: 10.5 years; range 4.3-14.3 years) and 17 of 51 (average donor age: 9.4 years; range 4.7-15.3 years) samples were found to react with the respective peptide (OD>0.7). Plasma interacting with each peptide was collected to create two pools and IgG was purified yielding approximately 30 mg total IgG/pool. These IgG preparations were then purified by affinity to the HB3var03 or the IT4var20 peptides to generate a human anti-HB3var03 peptide IgG preparation (pool A) and a human anti-IT4var20 peptide IgG preparation (pool B). The IgG yield was 0.2 mg for both preparations.

IgG was prepared from each pool according to manufacturer's protocol (GammaBind Plus Sepharose, GE Healthcare). Briefly, the plasma was diluted two fold in PBS, filtered and passed seven times through 1 ml packed slurry. After washing, the bound IgG was eluted in a total of 10 ml using Tris–glycine (pH 2.4), and neutralised by dialysis against phosphate-buffered saline.

The affinity purification was carried out according to the manufacturer's protocol. Briefly, 1 mg of each peptide was coupled to NHS columns (NHS activated HP, GE Healthcare). The dialysed IgG from each pool was passed through the respective column twice. Both the eluted sample and the run-through IgG were dialysed against phosphate-buffered saline and the affinity

purified IgG sample was concentrated up using a spin column (Vivaspin VS2022) to a concentration of 0.2 mg/ml.

ELISA

Unless stated otherwise, ELISAs were carried out as described here. Recombinant human EPCR and CD36 were coated at 3 μg/ml and PfEMP1 proteins/peptides and APC were coated at 5 μg/ml in PBS, overnight at 4 ^{o}C. Plates were blocked using PBS+3% skimmed milk (150μl/well) and washed three times after all steps with PBS+0.05% Tween-20 (all 50μl/well). All test samples and secondary antibodies were diluted in PBS+1% skimmed milk. All tests were run in duplicate and incubations were carried out at room temperature, for 1 hour with gentle shaking. Plates were developed using 100 μl of a phosphate solution with 0.012% H_2O_2 substrate and o-phenylenediamine per well. The colorimetric reaction was stopped with 100 μl of 3 M H_2SO_4 after 10 minutes and the optical density (OD) was measured at 490 nm. The quality of the APC was tested by confirming binding to EPCR.

For specific cases:

PfEMP1:EPCR binding: Recombinant CIDRα1 domains were added to EPCR coated plates at a concentration of 5 ug/ml. Secondary antibody anti-V5-HRP was diluted at 1:3000 (Invitrogen R96125).

Plasma sample screens for inhibition of CIDRα1:EPCR binding: Recombinant HB3var03 CIDRα1.4 or IT4var20 CIDRα1.1 protein was pre-incubated at 0.5 ug/ml with 25% plasma, as well as a no plasma control. The protein-plasma sample was then transferred to EPCR coated plates and developed using anti-V5-HRP at 1:3000.

Plasma sample screens for recognition of peptides: Plates coated with peptides as described were incubated with a 1:50 dilution of plasma in PBS+1% skimmed milk and developed using rabbit anti-human IgG-HRP (Dako P214) at 1:3000.
Testing CIDRα1 reactivity of affinity purified IgG: IgG pools were added to recombinant CIDRα coated plates at 1:50 in PBS+1% milk. Secondary rabbit anti-human IgG-HRP was added at 1:3000 for detection.

Testing CIDRα1 reactivity of affinity purified IgG: IgG pools were added to recombinant CIDRα or APC coated plates at 1:50 in PBS+1% milk. Secondary rabbit anti-human IgG-HRP was added at 1:3000 for detection.

Testing CIDRα1:EPCR binding inhibition of affinity purified IgG: Recombinant CIDRα1 at 0.0012 mg/ml was pre-incubated with nothing or with human IgG preparations at concentrations shown in Figure 6 and Figure S6 for 1 hour before transferring to plates coated with EPCR or CD36. Secondary anti-V5-HRP was added at 1:3000.

Parasite assays

The FCR3 IT4VAR20 parasite line was cultured and PfEMP1 expression was maintained by selection for binding to recombinant EPCR as described (Turner et al., 2013). The parasite adhesion assay was carried out as previously described (Turner et al., 2013). In short, HBMECs kindly provided by M. Stins (Johns Hopkins University) were grown to a monolayer over two days. Ring-stage infected erythrocytes were radioactively labelled with tritiated hypoxanthine the day before the adhesion assay. Radioactively labelled late trophozoite and schizont stages were purified and adjusted to 1.25×10^7 cells ml^{-1} in 2% FCS (in RPMI 1640). For binding inhibition, antibodies or proteins diluted in PBS were added to HBMECs in triplicates to final concentrations as shown in figure 3. PBS alone was added as a control. Twenty microlitres of late-stage infected erythrocytes were added to HBMECs and co-incubated for 1 h at 37 °C. Unbound infected erythrocytes were removed with a washing robot (Biomek 2000, Beckman Coulter) and radioactivity was measured on a Topcount NXT (PerkinElmer). The total amount of radioactivity added per well (max-value) was measured and adhesion was calculated as the proportion (percentage) of bound radioactively labelled infected erythrocytes out of the total amount of radioactively labelled infected erythrocytes added per well. The binding was then normalised into units by assigning the value 100 to the percentage radioactivity bound and recovered under optimal conditions.

Sequences of recombinant CIDR domains used in this study

>CIDRa1.4 HB3var03_HB3
PDCGVECKNETCTPKTVIYPDCGKNEKYEPPGDAKNTEINVINSGDKEGYIFEKLSEFC
TNENNENGKNYEQWKCYYDNKKNNNKCKMEINIANSKLKNKITSFDEFFDFWVRKL
LIDTIKWETELTYCINNTDVTDCNKCNKNCVCFDKWVKQKEDEWTNIMKLFTNKHD
IPKKYYLNINDLFDSFFFQVIYKFNEGEAKWNELKENLKKQIASSKANNGTKDSEAAIK
VLFNHIKEIATICKDNNTNEGC
>CIDRa1.1 IT4var20_IT4
PDCVVQCKGGKCTEDKKNDKCRSKIIKKILQSEEPTEIHVLNSDDKQGDITKKLEVFCS
STTNYEGRNVQKWKCYNKNSDYNNCEMNISSYKDSTDANVMLSVECFHSWAKNLLI
DTIKWEHQLKNCINNTNVTYCESKCIKNCECYEKWIKRKEHEWEKVKNVFGNNNRM
SYIYYNNLSRVFDSFLFQVMFALDQDEKGKWDQFTEDLKKKFEPSKTNTPTGKSQDAI
EFLLDHLKDNALTCRDNNSNESCDVSKKVKTNPC
>CIDRa1.1 PFD0020c_3D7
PDCVVECDGKTCTQKTDDDKNCRSKIIQKILESETPIEIEVLYSDDKQGVITEKLKDFCR
GPNNYNDENLQKWKCYNKNSEYNKCEMISWLYQDPKEYNLMLSVECFHSWAKNLLI
DTIRWEHQLKNCINNTNVTDCTSKCIKNCECYEAWIERKKDEWEKLKEVLNKKDETS
HNYYNKLKDVFDRFLFQVMFALDQDEKGKWDQFTEDLKKKFGPSVESAGTANSQDA
IEFLLDHLKDNALTCRDNNSIKPCTYPPNPTPNPC
>CIDRa1.8a MAL6P1.316_3D7
PACVVECDGGKCEEKKNSDGTCIEAQIYTVVRDETPTPIKVLFSGDHQKDITKKLSSFC
KNPESENNRDYQTWQCYYKSSDYNNCEMKGSLYKVEGDPNIIVSHECFHLWVQSLLI

DTIKWETKLKKCINNTNVTNCYNKCNKNCECFENWVEQKKKEWENVNDVYKDQKQ
SLGIYYEKLENLFKSNFFQVMKALEGDEKGKWYQFKDDLKKKFEPSEKNTRTTDSQD
AIKLILDHLKDNATTCKDNNSLEEDENC
>CIDRa1.6a PFD1235w_3D7
PECGVQCSGTTCTPKKVIHPNCKDKETYEPGDAKTTDITVLYSGDEEGDIAQKLQDFC
NDKNKENDENYEKWQCYYKSSEINKCQMTPSSHKVPKHGYIMSFYAFFDLWVKNLLI
DSINWKNDLTNCINNTNVTDCKNDCNTNCKCFENWAKTKENEWKKVKTIYKNENG
NTNNYYKKLNNHFQGYFFHVMKELNKEEKWYKLMEDLKEKIDSSNLKNGTKDSEGA
IKVLFDHLKDIAERCIDNNSKDSC
>CIDRa1.6b IT4var18_IT4
PYCGLDCGGKTCTAKQEIYPDCVYNGAYEPPNGAETTEITVLYNDNEGDMSKKLSQFC
SNENKENIENYQKWKCYYKDRDDIECEMISSSQKDEKHRKVMIFYNFFDLWVKNLLR
DSIKWEIELKDCINNTNVTDCNNKCNKNCECFDEWVKQKEKEWGSIKDVLKKESNM
PEKYYININKNFEFYFFRVMFELKKEEKWNKLMENLRIKIKSSKSNKGTIDSDGAIKVL
FDHLKEIAEKCIDNNSKDSC
>CIDRa1.6a HB3var02_HB3
PECGVQCSGTTCTPKKVIHPNCKDKETYEPGDAKTTDITVLYSGDEEGDIAQKLQDFC
NDKNKENDENYEKWQCYYKSSEINKCQMTRSSKKVPKHRGVMSFYAFFDLWVKNLL
IDSINWKNDLTNCINNTNVTDCNKDCNKNCVCFDQWVIKKEEEWKNVNKVFENKNI
DLHDYYNKLNKHFEGYFFHVMKELNKEEKWYKLMEDLKEKIDFSKGKADGKNSEGA
IKVLFDHLKEIAEKCIHNNSNESCEASTNRTPNPC
>CIDRa1.1 IT4var06_IT4
PDCVVQCDGKRCAEDTKDNNCRSKIIEEILQSEETTDIYVLYSGKGPGAITKKLHDFCS
NTNKEDRKYYKKWKCYNKNKDYNNCEMNISSYKDATDPNVMLSVECFHSWAKNLLI
DTIRWEHQLKNCINNTNVTDCKSKCNSNCQCYEKWIKEKEKEWPQVKGVLKKKDET
SHNYYDKLKDVFDRFLFEVKGALDQDEKGKWDQFTKDLEKKFGPSVESAGTANSQD
AIELLLDHLNDNAITCKDNNSLKEDKNC>CIDRa1.2 HB3var1csa_HB3
PDCGVKCDGIKYTHKSDNDRERVNNEDYKPPWGVKPTNITVLYSGNEQGDITQKLEN
FCNSSTNYKDKNNQKWECYYKDENINRCKLEQNTEINNDNPKIISFHNFFELWVTYLL
RDTIKWNDKLKTCINNTTTHCIDECNRNCLCFDRWVKQKEEEWNSIKKLFTKKKNM
QQSYYSNINNLFEGYFFKVMDKLDKNEAKWKELMENIKKKKNEFSNLKNNRDYLEN
AIELLLDHLKETSTICKDNNTNEAC
>CIDRa1.3 PFE1640w_3D7
PHCEVDCENGNCEVKNKPDGNCGKNVKYKPPYGVKPTEITVLYSGNEKGDISKKLSEF
CSNKNNINVKNNETWKCYYKNSDNNKCKMESNSENNKGAEKITSFHEFFELWVKNL
LKDTMKWENEIKDCINNTNITDCNDECNKNCVCFDKWVKQKEEEWKNVKKVFENK
KYIQDKYYLDINKLFESFLFKVISELDQGEAKWNQLKEELKKKIESSKANEGIKDSESAI
ELLLDHLKESATTCKDNNANEAC
>CIDRa1.4 DD2var32_DD2
PDCGVECNKGTCKKKPNDSNCRNNEAYIPPRDVTPTKINVLYSGDGHGDITKKLKGFC
SNPTDYDGKNYENWQCYYKSSEDNKCQMTSLSQTLQKHHYVMSFYAFFDFWVRKFL
IDTIKWENELKNCINNTTTDCSDGCNKHCVCFDKWVKQKEKEWNSIKKLFTKKNNV
PQPYYTNINNLFDSFYFQVMYELNHDEAKWKELTQELRNKINSSKKNTDAADSKDAI
ELLLEYLKEKSTICKDNNTNEAC
>CIDRa1.4 PF11_0521_3D7
PDCGVDCSSGTCIEKKDDINCGKKINYEPPHGVKPIDIIVLYSGNEEGEITKRLSEFCTDS
SNNKGKNYEQWKCYYKNGDDNKCKMVKNSGNNITEEKIISFDEFFYVWVRKLLIDSIK
WENELNNCIDNTSTHCNKECNKNCECFDKWVKKKEDEWKNVKNVFENKNGTSHNY

YNKLNGLFKGFFFEVMDKLNKDETKWNKLIESLRTKIDSSKENIGTGNTQDTIKVLLD
HLKETATICKDNNTNEAC
>CIDRa1.5a DD2var43_DD2
PDCGVECKNGRCKKKMDTGNNCGEPYIYNIPKNVNPIGINILNSDDEHVDIVKRLSEFC
RDSKKENGKNTEIWHCYYIGDKHNQCKMEKAVAENKHQTKITTFDFFFDLWIKNLLR
DTINWKSELKNCINNKNTEKCNKECNENCKCFEKWVKQKEQEWKNVKKVFENKNG
TSQNYYNKLKSHFDNYFFLVINNVNQGEEKWKKFTDELRKKMDFSKANTGTNDAQD
SIKILLDHEKKNAGTCLENNPSEPC
>CIDRa1.7 DD2var49_DD2
PHCGVVCNNGTCRDKPNNGNCGNNVIYNPPQGVTPTNLNVLYSADQEGDISNKLSEF
CNEEIEKNSQKWQCYYVNSYINACKMEKKNGNNTSEEKITKFHNFFEMWVTYLLTET
ITWKDKLKTCMNNTKTADCIDECSTNCVCFDKWVKQKEQEWNSIKELLTEEQKNPK
QNYGNINIYFESFFFHVMKKLNKEAKWNKLMDELRNKIELSKGNEGTKDLQDAIELLL
DHLKEIATICKDNNTNEAC
>CIDRa1.1 2110-3_2110
CPDCVVECVGGECKQKTDDDKNCRSKIIEEILKGEEPTVIDVLYSGNGQGVITKKLHDF
CSSTNKEDDKYYKKWKCYNKNSDYNNCELISSLSTDPNDPNVMLSIKCFDSWARNLL
VDALKWEHQLKNCINNTNVTDCKSNCNNNCKCYEEWIKRKGREWKQVKGVIENND
EGSHYYYKNVKNLFYNFLYPVIYKLEKEEKNGKWDQFMEDLKKKIESSETNTHTTDS
QDAIELLLDHLKENAITCKDNNSLEEDKNC
>CIDRa1.1 IT4var19_IT4
PDCVVVCEGGNCKEKTEDDNCRSEIIKKILKYETPTPIDVLYSGKGQGLITKKLEDFCSS
TTNYEGTNVQKWKCYNKNKDYNNCEMNISSYKDATDPNVMLSVECFHSWAKNLLID
TIRWEHQLKNCINNTNVTDCTSKCIKNCECYEKWIKEKEKEWPQVKGVLKKKDETSH
NYYDKLKDVFDRFLFEVKGALDQDEKGKWDQFTKDLEKKFGPSVESAGTANSQDAIE
LLLDHLNDNATTCKDNNSLAVENC
>CIDRa1.4 IT4var07_IT4
PDCGVICENGKCVVKENGSNCRHYNIYEPAPDVKTTEINVIVSGDEQGIITKKLQDFCM
NPNNENGTNNQIWKCYYKDEKENKCKVETKSGNSTYKEKITSFDEFFDFWVRKLLID
TIKWETELTYCINNTTNADCNNECNKNCVCFDKWVKQKEKEWKNIMDLFTNKHDIP
KKYYLNINDLFNSFFFQVIYKFNEGEAKWNKLKENLKKKTESSKKNKGTKDSEAAIKV
LFDHLKETATICKDNNTNEAC
>CIDRa1.1 IGHvar19_IGH
PDCVVVCSNGTCSQKKDDDHCRSEIIKKILKYETPTPIEVLYSGDGQGLITEKLHEFCRG
PNKVDSKNYKTWNCYNKNNDYNKCEMISWLYEDPKKSNLMLSIQCFDSWVQNLLID
TIKWEYELKDCINNTYDADCNDECNKNCECYEKWIKQKEKEWQKVKNVFGNNNRM
SYIYYNNLSRIFDSFLFPVIYKLQKEEKDGKWDQFTKDLKKKFESSETNTPTGNSQDAI
EFLLDHLKDNATICKDNNTNEAC
>CIDRa1.1 PFCLINvar30_PFCLIN
PDCVVKCDGKTCEQKKDDENCRSKIIQKILQGEEPTVIDVLYSGKGQGLITKKLHDFCS
STNKEDDKYYKKWKCYNKNSDYNNCELISSLSTDPTDPKVMLSIKCFDSWARNLLVD
ALKWEHQLKNCINNTNVTDCKSNCNNNCKCYEEWIKQKEKEWQKVKGVLKKKDKN
SDNYYKNVKNLFYSFLFQVIYELEKEEKNGKWDQFMEDLKKKIEASQKNKGTENSQD
AIELLLDHLKDNATICKDNNTNEAC
>CIDRa1.1 RAJ116var08_RAJ116
PDCVVDCTGGNCKENKKHDNCRSKIIEEILRSEETTDIDVLYSGKGPGAITKKLHDFCS
NPNNYKRANVQKWKCYNKNKDYNNCEMNISSYKDATDPNVMLSVQCFYSWAQNLL
IDIIRWEHQLKDCINNTNVTDCDNECNKNCECFEKWIKQKQKEWQQVKEVLKKKDE

NSHNYYNKLRSVFDSFLYQVMFALNNEEKGKWDQFTEDLEKKFESSKKSAGTGNSQD
AIEFLLDHLNDNAITCKDNNSKESC
>CIDRa1.1 GA011_ERS009959
PDCVVYCEGGECKENENGDNCRSEIIKEIVRSETPTVIDVLYSGNGQDHITEKLDHFCSS
TNKEDRKYYKTLKCYNKNNDYNKCEMISWLYQDPKESNLMLSIQCFYSWAQNLLIDT
IRWEHQLKDCINNTNVTDCKSNCNKNCKCYKKWIEQKGSEWQQVKQVLKKKDKNSE
NYYDKLKDVFDTLYFEVMHELNKGKEKGKSEELTEDLKKKFGPSKKNTDAADSKDAI
EFLLDHLNDNAITCKDNNSLAVENCTRTKSNPC
>CIDRa1.5a 1965-2_1965
PDCGVECKNGRCKKKTDADGNCGKTQIYDIPKDVTPTDINVLYSGDEYGDIAKRLSEFC
RDSKKENGKNTEIWHCYYIGDKHNQCKMEKAVAENKHQTKITTFDFFFDLWIKNLLR
DTINWKSQLKNCINNTNKTNCNKTCNENCKCFENWVNQKEKEWNNMKELFKNKN
RTSQNYYNKLKSHFDNYFFLVINNVNKGEAKWKKFTDELRNKIDSSKAKNGTSDSQD
SIKMLLEHLKEDGTTCTANNPDSICNTPGTDARSLEPASKIEPQNKDTRTNPC
>CIDRa1.5a GA013_ERS010323
PYCGVDCSNGTCKENGNDDNCGEQQNYNIPKGVDPTNINVLYSVDGHGDIAKRLSTFC
NDKNNKNAKNNEIWQCYYIGHKNNQCKIEKSVSQKKHQTKIAPFDYFFDLWVTNLLR
DNIDWKNELKNCINNKNTEKCNKECNDNCKCFQSWLNKKEDEWNKITGLLKNKNGI
LQNYYNKLKSHFDYYFFQVINNINKGEEKWKKLKEDLKKEIDFSKLKTNTGDSQDSIK
LLLDHENKNAGTCLKNNPIDSCPKAEPQKSDEKVQPQDTPPNSC
>CIDRa1.5a GA014_ERS010022
PYCGVDCNGTTCTPKTVIYPDCGNNDVYEPPRGVTPTDINVLYSGDEYGDIAKRLSEFC
NDKNNKNGKKNETWKCYYENSEKNMCKMDKNSKNHTSEEKITSFDYFFDLWVTNL
LRDTINWKNDLMNCINNKKMKNCNKTCNDNCKCFQSWLNKKEDEWNKITGLLKNK
NRTSQNYYNKLNNHFQGYFFQVTNEVNKDEAKWNQFTEELRKKMDFSKANTGTND
SQDAIKVLLEHLKEDGKTCTANNPDSACNNPKAARIIIRATTRNPC
>CIDRa1.5b 1918-5_1918
PDCGVKCTNGKCEEKTDVDGNCGNKETYIPPRDISPTEISVLYSGYKRDDISEKLETFC
REPTNNKSKNNETWKCYYKHSYNNKINKCIRQNEENIKNKLIINLDTFFEFWVRSFLN
DTIDWKYDLNTCMNFTNTTKCNNNCNKNCKCFEQWVNKKETEWKNVAQYFFKHN
EISKKKYCEILKDIFENYYVEVIKKVFKGDNKWKEIMVDIKNKIDCSNLKNGTEHLEKII
NVLINQEQADATTCLKNNPIESCPKAEPQKSDENTQPQDTPPNSC
>CIDRa1.5b 1983-13_1983
PDCGVECNGKTCTKKEETDENCGKPPNYTIPTDVTPTDINVLYSGDEQGYITKRLSEFC
GPTKNYIGKKNELWKCYYDDKQKNNNNNNNICKLQPNAHIKNENIIINFDNFFEFWV
RRFLNDTIDWKYDLNTCMNFTNTTKCNNNCNKNCKCFEQWVNIKKTEWKNVTEYF
FKHDETSKKKYCEILKDIFENYYVEVIEKFYKGKHKWKERIEKLKNMDCSQVKIGTNA
SQDQIDIFLSNLKGDATQCTSKNPKSACNKPHAARIITRATTRNPC
>CIDRa1.5b GA017_ERS010601
PDCGVECTKGRCEKKAETDDNCGKPPIYTIPTDVTPTDINVLYSGDEQGYITKRLSKFC
GPRKNYIGKKNELWKCYYDDKHNNKDNICITDTNQKITNRYIMNFDNFFEFWVRRLL
IDTINWEYKLKTCIDNNNNDKCISGCNEDCKCFDKWVDQKEKEWKSVKKHIAKEKEI
GKNKYCEKLKKIFSDYYVQVIETIYKGKHKWKRRIESIKKIDCSQVKIGNKDSEYQIDTF
FRSVKEGATQCTSKNPKSACDKPKAARIITPATTRNPC
>CIDRa1.6b GA018_ERS010570
PYCGLDCVGTECTAKEEIYPDCVYNGEYDPPKDVRPTEINVIDSGNAVNISEKLNDFCT
NPTNPNDKIYQKWQCYYKSSKVNKCQMTSLTQTLQKHHYVMTFYIFFDLWVKNLLR
DSVKWEIELKDCINNTNVTDCNNKCNKNCVCFDKWVKQKKKEWDSIKKLFKTEKDE

MKKYYTNINKNFEFFFFRVMYELNNEEAKWNKLMENLRIKIKSSKRNKRTKDSEGVI
KVLFDHLKETATICKDNNTNEACVSSQNATTNPC
>CIDRa1.6b GA019_ERS010031
PYCGLDCVGKTCTAKQEIYPDCVYNGDYEPPNGAETTEITVLYNDNEGDMSKKLSQFC
SNENKENIENYQKWKCYYKDRDDIECEMISSSQKDEKHRKVMIFYNFFDLWVKNLLR
DSIKWEIELKDCINNTNVTDCKSVCNVNCECFDKWVKQKEKEWDSIKKLLKKKKNVS
KKYYTNINKNFEFFFFRVMYELNNEEAKWNKLMENLRTKINSYKKNKRTKDSEGAIK
VLFDHLKETATICKDNNTNEACVSSQNATTNPC
>CIDRa1.7 IT4var22_IT4
PYCGLDCGGKTCTAKQEIYPDCVYNGAYEPPNGAETTEITVLYSADQEGDISNKLSEFC
NDENNKNSQKWQCYYVSSENNGCKMEKKNANHTPEVKITKFHNFFEMWVTYLLTE
TITWKDKLKTCMNNTKTADCIHECNKNCVCFDKWVKQKEDEWNSIKKLFTKEKKM
PKQYYGNINIYFESFFFHVMKKLNKEAKWNKLMDELRNKIELSKGNEGTKDLQDAIEL
LLEYLKEKSTICKDNNTNEACDPTVDPTKNPC
>CIDRa1.7 1965-8_1965
PHCGVDCNGKKCTLKSDNDPQCVNKLKYEPPYGVKPTEITVLYSADQEGDISKKLSEF
CNDEKKINSKNIETWKCYYKSTYNNACKMDKNSKNHTPEVKITKFHNFFEMWIVYLL
TETITWNDKLKTCMNNIKTTDCIDECNTNCVCFDKWVKQKGKEWNSIKKLFIKEQKN
PKQNYGNINIYFESFFFHVMDKLNHDEAKWNELMNELKQIIDSSKANTDNKNLQDAI
KVLFDHLKEIATICKDNNTNEACEDSKKETQNPC
>CIDRa1.4 1918-3_1918
PDCGVECNGETCTPKKEKYPECLNKEIYTPNGAKTTEINVIVSGNEQGDITKKLEDFCN
NSTNYKGKNYQKWQCYYENSKKNMCKMDKNSKNHTSEEKITKFHNFFELWVIYLLT
ETIRWNDKIKNCINNTTIHCIDKCNKNCVCFDKWIKQKEQEWNSIKKLFIKKQKMPN
EYYLNIKNHFEGYFFHVMKKLNKEAKWNELMENLRTKINSSKKNKGTKDSEGAIKVL
LDHLKETATICKDNNTNEGCVSSKKSKTNPC
>CIDRa1.7 1914-14_1914
PDCGVQCNGEKCKEKEYDHECENDEDYGLPSDVTPIDITVLYSGNEAGDISEKLKDFC
NISTKYEGKNYENWQCYYKNKNKNKCKMEQNSKKDKDKPKITKFHNFFELWVIYLL
TETIRWNDKIKNCMNNTNITDCSDGCNKHCVCFDKWVKQKEEEWNSIKKLFIKEQE
MPNEYYLNIKNHFEGYFFHVMDKLNHDEAKWKELTQELKKKIDSSKEKPGTKDSESA
IELLLDHLKEIATICKDNNTNEACASSKKSKTNPC
>CIDRa1.4 GA024_ERS010438
PDCGVECTNGRCEKKAETDGNCGNKETYKPPHGVEPIDITVLYSGNEERÉITKRLSEFC
TDSSNNKGKNYENWQCYYKNGDDNKCQMTSLTQTDEKHRYVMTFHKFFNFWVRNL
LIDTINWETDLRNCLNNTGITDCNDGCNNNCTCFDKWVKQKEGEWNSIKLLLAKEQ
KMPKKYYLNINDLFDSFFFEVMDKLNKDETKWNKLKENLKKKIKSSNENRGTKDSES
AIELLLDHLKEIATICKDNNANEACETSRNRKTNPC
>CIDRa1.8a GA026_ERS010178
PDCVVDCKGAKCEQKMKPDGTCEKPQIYTRPKDVTPKIIKVLFSGDNQEDITEKLSSFC
SNPKSKIDRNYQTWKCYYKSGHYNICEMKGSLYKYPEHPNDMLSVECFHLWVKNLLI
DTIKWETKLKKCINNTNVTDCDNECNKNCECFENWVEQKKKEWEEVRKVYKDQKE
SLGIYYKKLENLFDTYYFEVMGALENEEKHGKWNQLTAKLKQIIESHKKNRHSGNSQ
DAIELLLDHLKETATTCKDNNSNESCDVSKDSKTNPC
>CIDRa1.8b 2053-3_2053
PICGVKCENKSCTDKENDDDCKNKKKYDPPKGVTPIDIPILYSGDKQGDITKKLEDFCY
NRTKENEKTYQNWKCYYKDSEFNKCKMESKSGKSTTQEKIISFDEFFYLWVNNLLIDS
IMWENDIKHCINNTNVTNCKNKCNENCKCFKNWVKKKEEEWTKVKQILGNRSENLN

NYYNKLNSLFKGFFFEVVYKFNNKEEKWNKLTEKLEQKIGSSKGKEGVENPKDAIELL
LDHLKENAITCKDNNSLEEDKNCPKIKINPC
>CIDRa1.8b 1702-3_1702
PDCIVVCDYKGCKENKNDENCKSKRTYSLPPDVNSTEIEVLFSGDNQEDIVEKLSSFCK
NTNNENGENVEKWECYYESEHNNKCKMTSPKHKDEKRPTVMIFDEFFDFWVTHLIK
DTIKWESDINDCINNTNVTDCDSACNENCKCFDEWVEKKEVEWGNVKKVLGNRSEN
LNIYYNKLNGIFSGFFFGVMHELKKKEAKQGVKAEEAQEAEETQEAQEEEAKWNKLT
AKLQEIIDSSKGKADTANSQDAIKPLLEYIKETATTCIDNNSLAVENCPKTKINPC
>CIDRa1.8b GA029_ERS010532
PDCVVVCERGNCTEKKGDDKCRKKYTYEIPAGMESTKIDILFSGENQEDITEKLRSFCK
NTNNENGENIEKWECYYKNEYDNKCKMTSLKREDQKHHDLMSYYEFFDFWVTHLIK
DTIKWESDINDCINNTNVTNCNNGCNENCICFEKWVGQKEKEWENVKKVLKNPSEN
LNNYYNKLNGIFSGFFFGVMHELKKKEAKQGVKVEKAEKSEEAQEAQEEEAKWNKLT
AKLEEIIKSHKENTGTGNTQDAIEPLLKYLDENAITCKDNNSLKEDKNCPKTKKNPC
>CIDRa1.6a PF08_0140_3D7
PDCGVEYKNGRYTAKDQKYPDCRNEKYDPNNAETTDITVLYSGDVGDFSEKLQDFCN
DINNGKVKNYQIWQCYYENSEINKCQMTPSSHKVPKHGYIMSFFAFFDLWVKNLLIDT
INWKNELTNCINNTNVTDCKNDCNTNCKCFENWAKTKEKEWENVKTIYKNENRNT
NNYYKKLNDLFKGYFFHVMYELNNEEAKWNKLMKNLRTKIDSSRKNAGNEDSEGAI
KVLFDHLKDIAERCIDNNSIKPC
>CIDRa1.4 GA045_PREICH
PECGVKCEHGSCRDKPKDDHCINKNEYDPPRDVTPTNITVIYSGNEEGEITKRLSAFCT
DSSKDEGKNYQKWECYYVNSDSNRCKMDKTSGKNMTEDKITSFDEFFYSWVQNFLID
TIKWENELNNCINNTILTDCNDGCNSNCVCFDKWVKKKENEWKKVKNVFENKSGIS
DNHYKKLNGLFDSFFFPVMHALNEQEKRKWKELTKKLEEISGFSKGKTGTVNSQDTI
KVLLDHLKENATICKDNNTNEAC
>CIDRa3.1 DD2var01_DD2
PYCGVKKVNNGGSSNEWEEKNNGKCKSGKLYEPKPDKEGTTITILKSGKGHDDIEEKL
NKFCDEKNGDTINSGGSGTGGSGGGNSGRQELYEEWKCYKGEDVVKVGHDEDDEEDY
ENVKNAGGLCILKNQKKNKEEGGNTSEKEPDEIQKTFNPFFYYWVAHMLKDSIHWK
KKLQRCLQNGNRIKCGNNKCNNDCECFKRWITQKKDEWGKIVQHFKTQNIKGRGGS
DNTAELIPFDHDYVLQYNLQEEFLKGDSEDASEEKSENSLDAEEAEELKHLREIIESED
NNQEASVGGGVTEQKNIMDKLLNYEKDEADLCLEIHEDEEEEKEKGDGNECIEEGENF
RYNPC
>CIDRa3.5 IT4var15_IT4
PGCGVELIGNEWKEKNKGECKGGKRYNIPKGTKHNVIPVLSFGDEHKEIIEKIEQFCAE
SNSDSSKLTEQWKCYYGDKEYEVCTLENRNKSEEDPEEIQKTFHNFFYFWIRHLLNDS
IEWRDKINNCIEKAKEGKCKNECKTDCGCFQRWIGKKKEEWGEIKKHFKTQDGFSIFG
NNYDFVLENVLNIDELFQDITEAYGNSQKIQGIKDTLAKKKTQAADDATEQKNTIDLL
FEYDSEEAEKCKKIQEECQPKKPTKVRNPC

Supplemental figure legends:

Supplemental Figure 1 – Binding of CIDRα1 variants to EPCR – related to Figure 1

A. ELISA was used to study binding of 40 recombinant CIDRα1 and two CD36-binding CIDRα3 variants binding to EPCR and CD36. Domains were classified as EPCR binders if OD > 0.1. **B.** Surface plasmon resonance was used to quantify the binding of CIDRα1 domains to an EPCR coupled surface. EPCR was coupled by capture using a biotin attached to a BAP tag at the N-terminus and the chip was regenerated and coupled with fresh EPCR after each cycle. CIDRα1 domains were injected at concentrations described in Table S2.

Supplemental Figure 2 – Structural and biophysical characterisation of the CIDRα1:EPCR interaction – related to Figure 2

A. An isothermal titration calorimetry experiment was carried out using a MicroCal iTC200 machine in which HB3var03 CIDRα1.4 domain was placed into the cell and subjected to injections of 36 μM EPCR at 25°C. **B.** Multi-angle laser light scattering (MALLS) measurements of HB3var03 CIDRα1.4 (red, MW=26.3 kDa), EPCR (orange, MW=22.1 kDa) or the HB3var03 CIDRα1.4:EPCR complex (purple) show the formation of a 1:1 complex with a mass of 47.8 kDa . **C.** Equilibrium analytical ultracentrifugation of the HB3var03:EPCR complex revealed a mass of 47.1 kDa, demonstrating formation of a 1:1 complex. **D.** The theoretical scattering calculated from *ab initio* reconstructions (yellow is for HB3var03 CIDRα1, red for EPCR and blue for the CIDRα1:EPCR complex), superimposed into experimental scattering data. Guinier plots are superimposed. **E.** Distance distribution functions of HB3var03 CIDRα1 (yellow), EPCR (red) and the CIDRα1:EPCR complex (blue), derived from small angle x-ray scattering. **F.** The structures of HB3var03 CIDRα1, EPCR and their complex, docked into *ab initio* molecular envelopes calculated from scattering data. **G.** The theoretical scattering calculated from *ab initio* reconstructions (blue is DD2var32 DBLα1.7-CIDRα1.4-DBLβ1, red for EPCR and green for the DD2var32:EPCR complex), superimposed into experimental scattering data. Guinier plots are superimposed. **H.** Distance distribution functions of DD2var32 DBLα1.7-CIDRα1.4-DBLβ1 (blue), EPCR (red) and the DD2var32:EPCR complex (green), derived from small angle x-ray scattering. **I.** A representation of the HB3var03:EPCR crystal structure (molecules A and B), with the thickness of the wire representing the B factors associated with the residue showing the interface between HB3var03 CIDRα1.4 and EPCR to be ordered while loops from the CIDRα1.4 domain are flexible. **J.** The electron density, contoured at 1.0 σ for the residues from HB3var03 CIDRα1.4 that form the helix and kinked helix at the heart of the EPCR binding site.

Supplemental Figure 3 - A sequence logo describing conservation across the EPCR-binding CIDRα1 domains – related to Figure 2

All sequences of CIDRα1 subclasses 1.1 and 1.4-8 were aligned and a sequence logo generated (of positions corresponding to residues of the HB3var03 CIDRα1 domain). Residue positions are numbered according to the position in HB3var03. Below the logo is shown the secondary structure in HB3var03. Size polymorphisms are indicated >< for deletions and <> for insertions (see table for details). Residues that make direct contact with EPCR are annotated with * above the logo. Cysteines are labelled, with C5-C12, C6-C7, C8-C10, C11-C13 forming disulphide bonds in HB3var03.

Supplemental Figure 4 – Binding of HB3var03 CIDRα1 mutants to EPCR – related to Figure 3

Surface plasmon resonance was used to quantify the binding of mutants of the HB3var03 CIDRα1.4 domain to an EPCR coupled surface. EPCR was coupled using a biotin attached to a BAP tag at the N-terminus and the chip was regenerated and coupled with fresh EPCR after each cycle. CIDRα1 domains were injected at concentrations described in Table S6.

Supplemental Figure 5 – Sequence diversity of the nine EPCR interacting residues of CIDRα1 domains

A maximum likelihood tree built on the nine residues that directly interact with EPCR forms subgroups similar to trees built on the whole domains. Branches are coloured according to the sub-classification of the whole domain given in Figure 1. Sequence logos for each CIDRα1 subclass show the diversity of the residues making direct contacts with EPCR.

***Supplemental Figure 6* – Human IgG pool reactivity to recombinant CIDRα1 domains – related to Figure 6**

A. Two human IgG pools were made from human plasma samples selected after screening for reactivity to either a UPSA CIDRα1 (HB3var03 CIDRα1.4) or a UPSB CIDRα1 (IT4var20 CIDRα1.1) as described in materials and methods. From the pools, IgG was affinity purified on 63-amino acid peptides corresponding to the EPCR binding region of either HB3var03 or IT4var20 CIDRα1 domains. The graph shows CIDRα reactivity of the IgG pools prior to purification, the purified IgG pools and the run-through IgG (that did not stick to the peptide) pools. **B.** The reactivity of IgG purified on the HB3var03 CIDRα1 domain was tested against activated protein C, showing negligible cross reactivity.

Supplemental references:

Altschul, S. (1997). Gapped BLAST and PSI-BLAST: a new generation of protein database search programs. Nucleic Acids Res. 25, 3389–3402.

Crooks, G.E., Hon, G., Chandonia, J.-M., and Brenner, S.E. (2004). WebLogo: a sequence logo generator. Genome Res. 14, 1188–1190.

Edgar, R.C. (2004). MUSCLE: multiple sequence alignment with high accuracy and high throughput. Nucleic Acids Res. 32, 1792–1797.

Franke, D., and Svergun, D.I. (2009). DAMMIF , a program for rapid ab-initio shape determination in small-angle scattering. J. Appl. Crystallogr. 42, 342–346.

Higgins, M.K. (2008) The structure of a chondroitin sulfate-binding domain important in placental malaria. J Biol Chem. 283, 21842-6.

Konarev, P. V., Volkov, V. V., Sokolova, A. V., Koch, M.H.J., and Svergun, D.I. (2003). PRIMUS : a Windows PC-based system for small-angle scattering data analysis. J. Appl. Crystallogr. 36, 1277–1282.

Miotto, O., Almagro-Garcia, J., Manske, M., Macinnis, B., Campino, S., Rockett, K.A., Amaratunga, C., Lim, P., Suon, S., Sreng, S., et al. (2013). Multiple populations of artemisinin-resistant Plasmodium falciparum in Cambodia. Nat. Genet. 45, 648–655.

Shi, J., Blundell, T.L., and Mizuguchi, K. (2001). FUGUE: sequence-structure homology recognition using environment-specific substitution tables and structure-dependent gap penalties. J. Mol. Biol. 310, 243–257.

Svergun, D.I. (1992). Determination of the regularization parameter in indirect-transform methods using perceptual criteria. J. Appl. Crystallogr. 25, 495–503.

Svergun, D.I. (1999). Restoring low resolution structure of biological macromolecules from solution scattering using simulated annealing. Biophys. J. 76, 2879–2886.

Tamura, K., Stecher, G., Peterson, D., Filipski, A., and Kumar, S. (2013). MEGA6: Molecular Evolutionary Genetics Analysis version 6.0. Mol. Biol. Evol. 30, 2725–2729.

Vistica, J., Dam, J., Balbo, A., Yikilmaz, E., Mariuzza, R.A., Rouault, T.A., and Schuck, P. (2004). Sedimentation equilibrium analysis of protein interactions with global implicit mass conservation constraints and systematic noise decomposition. Anal. Biochem. 326, 234–256.

Volkov, V. V., and Svergun, D.I. (2003). Uniqueness of ab initio shape determination in small-angle scattering. J. Appl. Crystallogr. 36, 860–864.

Wernersson, R. (2006). Virtual Ribosome--a comprehensive DNA translation tool with support for integration of sequence feature annotation. Nucleic Acids Res. 34, W385–8.

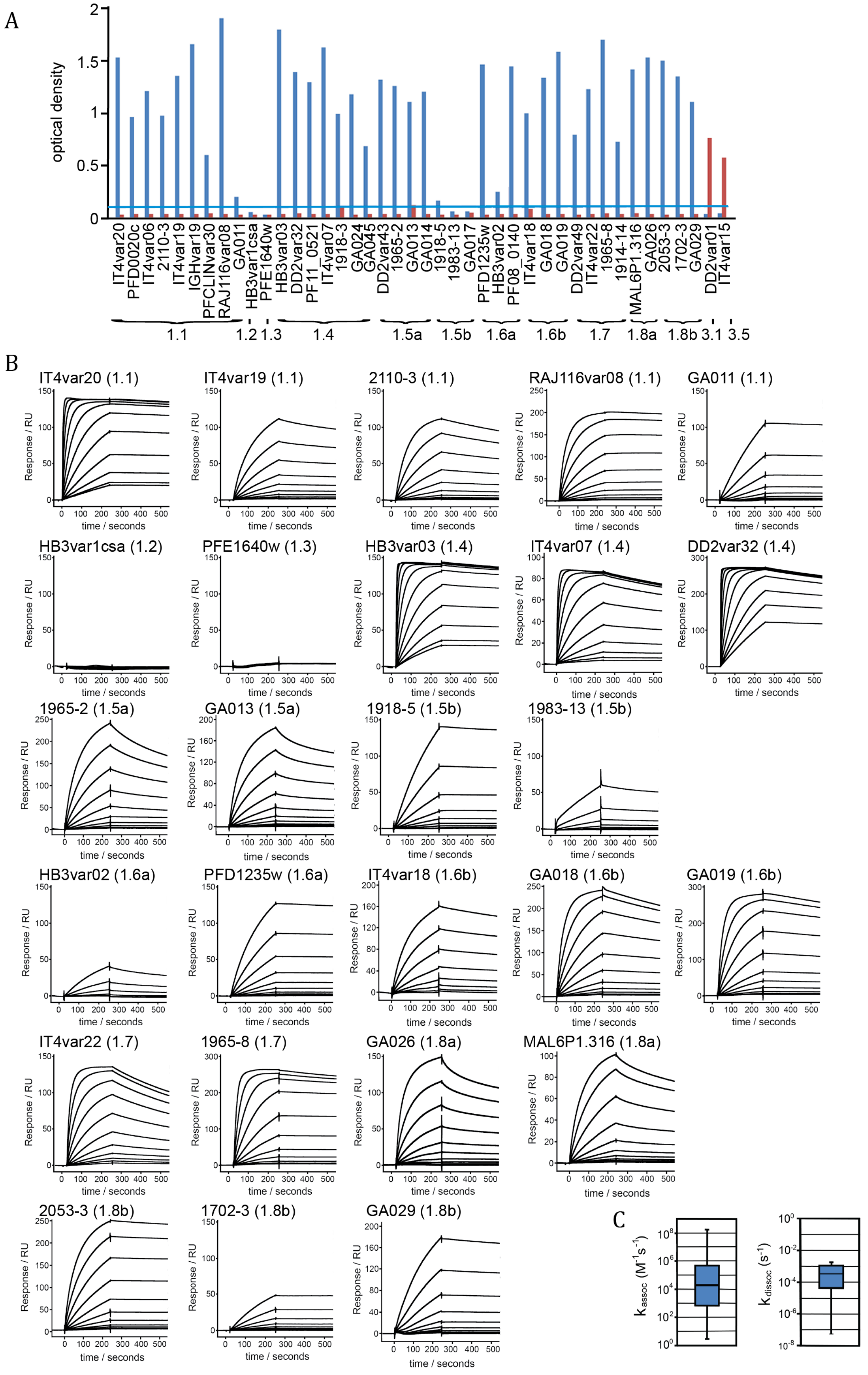
A
optical density
IT4var20
PFD0020c
IT4var06
2110-3
IT4var19
IGHvar19
PFCLINvar30
RAJ116var08
GA011
HB3var1csa
PFE1640w
HB3var03
DD2var32
PF11_0521
IT4var07
1918-3
GA024
GA045
DD2var43
1965-2
GA013
GA014
1918-5
1983-13
GA017
PFD1235w
HB3var02
PF08_0140
IT4var18
GA018
GA019
DD2var49
IT4var22
1965-8
1914-14
MAL6P1.316
GA026
2053-3
1702-3
GA029
DD2var01
IT4var15
1.1
1.2
1.3
1.4
1.5a
1.5b
1.6a
1.6b
1.7
1.8a
1.8b
3.1
3.5
B
IT4var20 (1.1)
IT4var19 (1.1)
2110-3 (1.1)
RAJ116var08 (1.1)
GA011 (1.1)
HB3var1csa (1.2)
PFE1640w (1.3)
HB3var03 (1.4)
IT4var07 (1.4)
DD2var32 (1.4)
1965-2 (1.5a)
GA013 (1.5a)
1918-5 (1.5b)
1983-13 (1.5b)
HB3var02 (1.6a)
PFD1235w (1.6a)
IT4var18 (1.6b)
GA018 (1.6b)
GA019 (1.6b)
IT4var22 (1.7)
1965-8 (1.7)
GA026 (1.8a)
MAL6P1.316 (1.8a)
2053-3 (1.8b)
1702-3 (1.8b)
GA029 (1.8b)
Response / RU
time / seconds
C
k_assoc (M^-1 s^-1)
k_dissoc (s^-1)

Figure S1

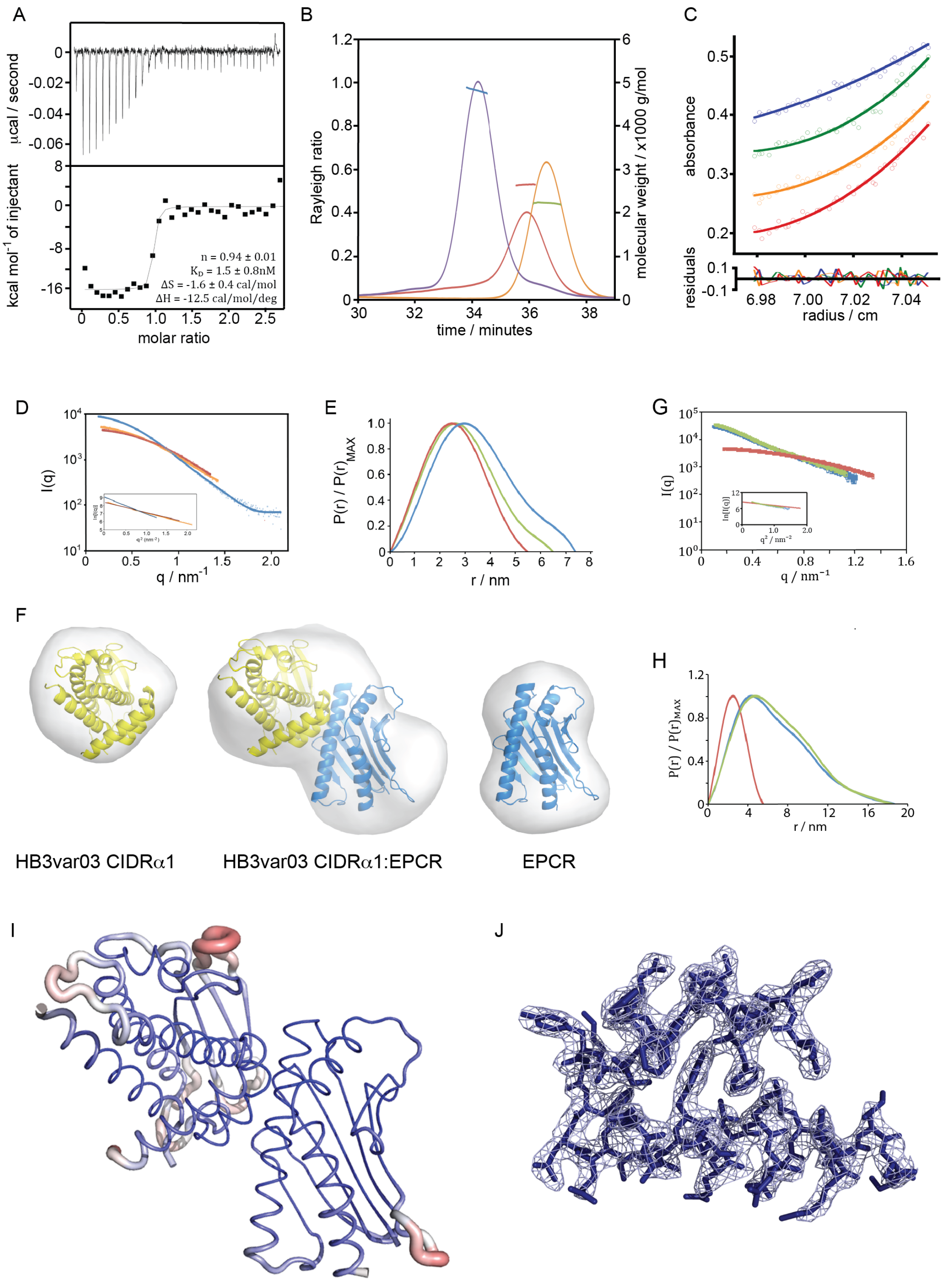
A
µcal / second
kcal mol^{-1} of injectant
molar ratio
n = 0.94 ± 0.01
K_D = 1.5 ± 0.8nM
ΔS = -1.6 ± 0.4 cal/mol
ΔH = -12.5 cal/mol/deg
B
Rayleigh ratio
molecular weight / x1000 g/mol
time / minutes
C
absorbance
residuals
radius / cm
D
I(q)
q / nm^{-1}
E
P(r) / P(r)$_{MAX}$
r / nm
G
I(q)
q / nm^{-1}
ln[I(q)]
q^2 / nm^{-2}
F
HB3var03 CIDRα1
HB3var03 CIDRα1:EPCR
EPCR
H
P(r) / P(r)$_{MAX}$
r / nm
I
J

Figure S2

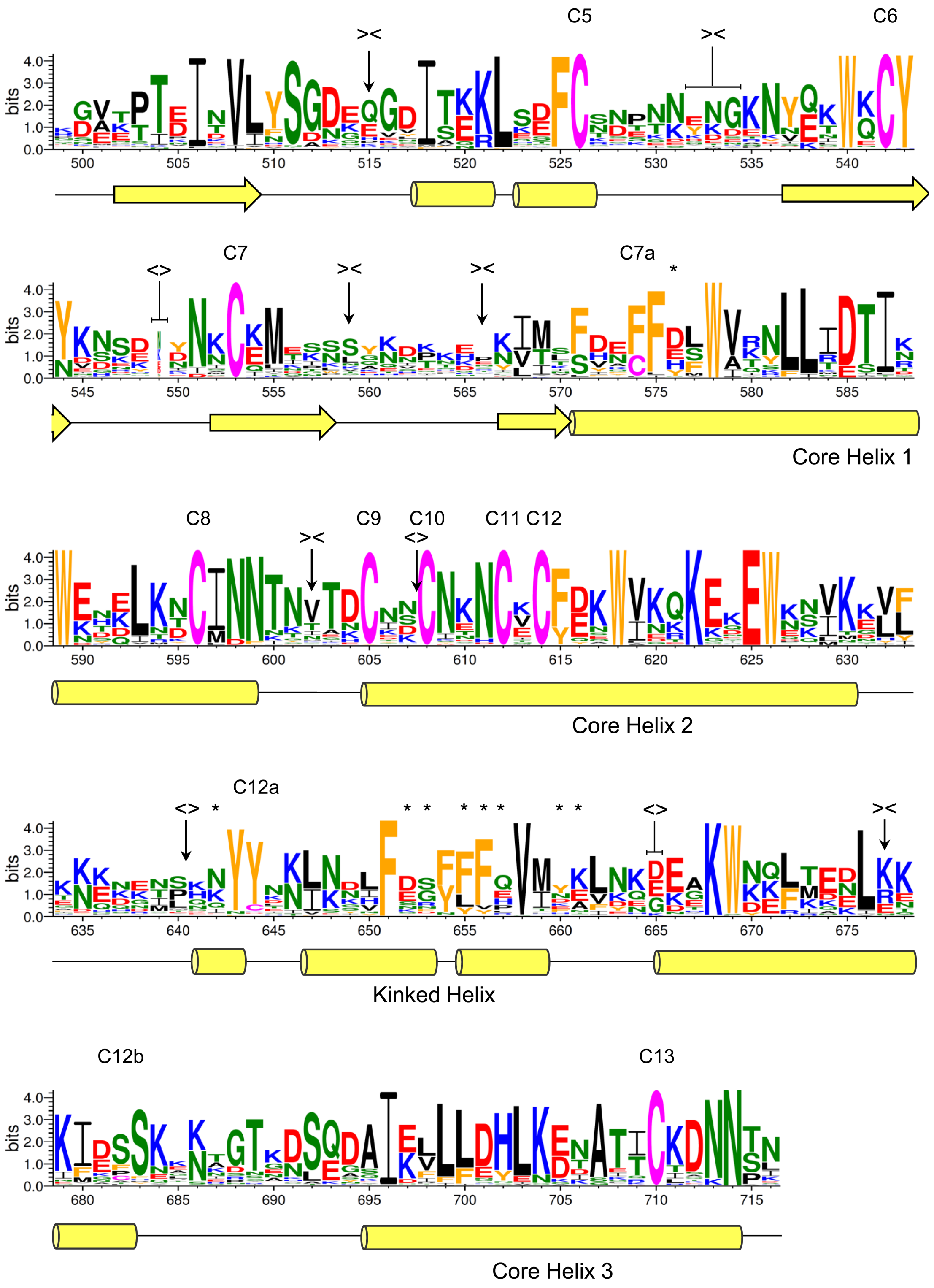

C5
C6
C7
C7a
Core Helix 1
C8
C9
C10
C11 C12
Core Helix 2
C12a
Kinked Helix
C12b
C13
Core Helix 3
bits

Figure S3

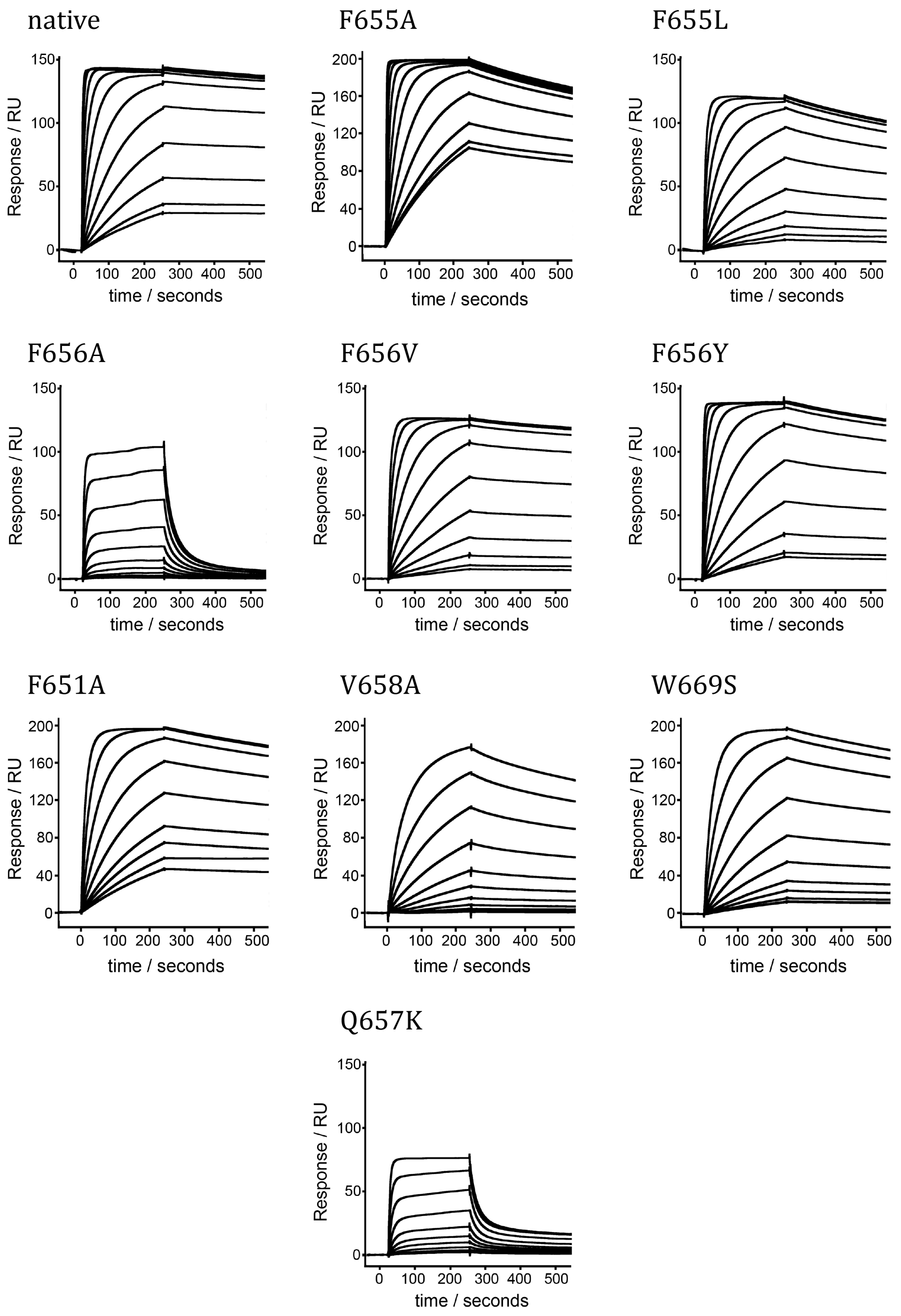
native
F655A
F655L
F656A
F656V
F656Y
F651A
V658A
W669S
Q657K
Response / RU
time / seconds
0
50
100
150
200
40
80
120
160
300
400
500

Figure S4

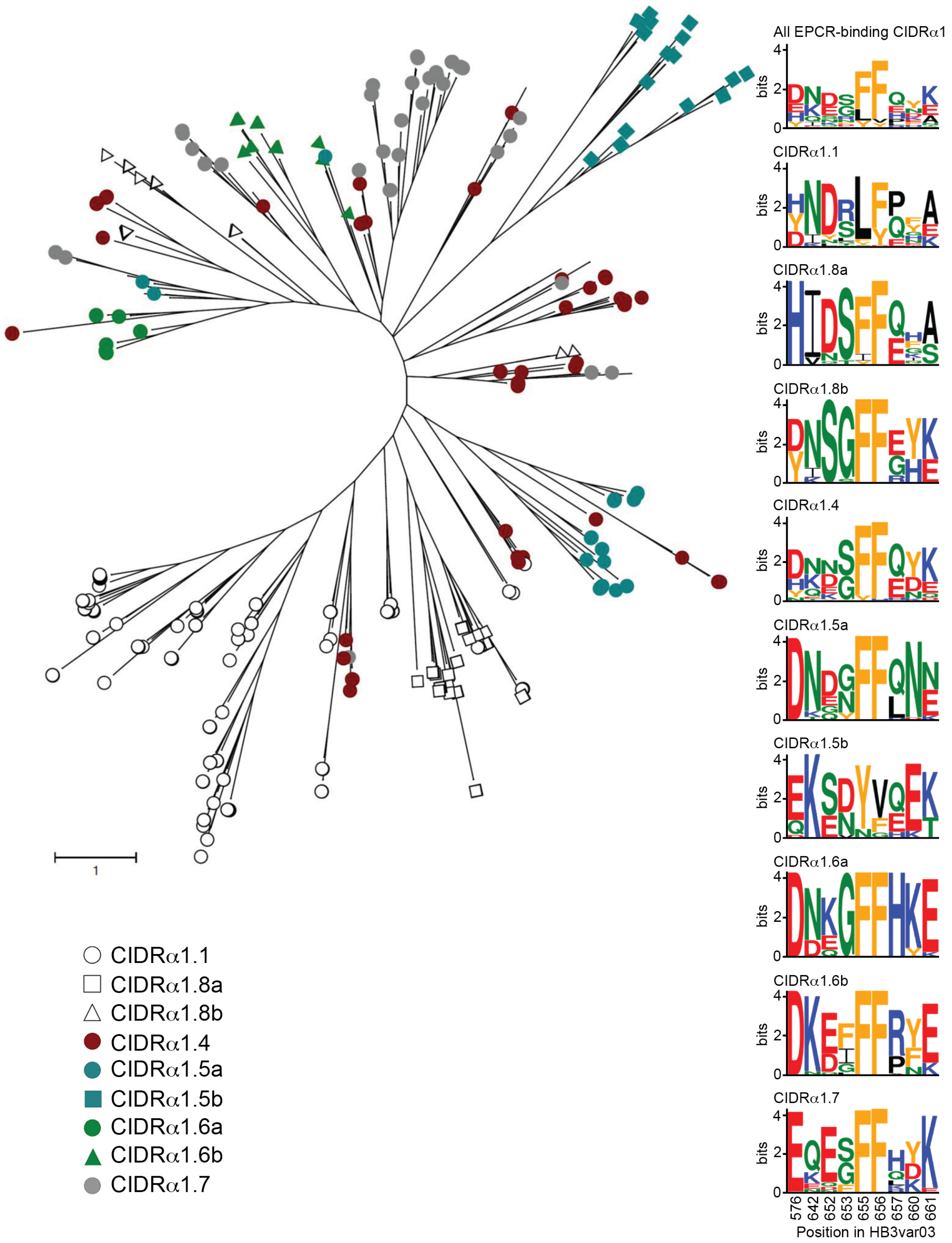
All EPCR-binding CIDRα1
CIDRα1.1
CIDRα1.8a
CIDRα1.8b
CIDRα1.4
CIDRα1.5a
CIDRα1.5b
CIDRα1.6a
CIDRα1.6b
CIDRα1.7
bits
576 642 652 653 655 656 657 660 661
Position in HB3var03
1
CIDRα1.1
CIDRα1.8a
CIDRα1.8b
CIDRα1.4
CIDRα1.5a
CIDRα1.5b
CIDRα1.6a
CIDRα1.6b
CIDRα1.7

Figure S5

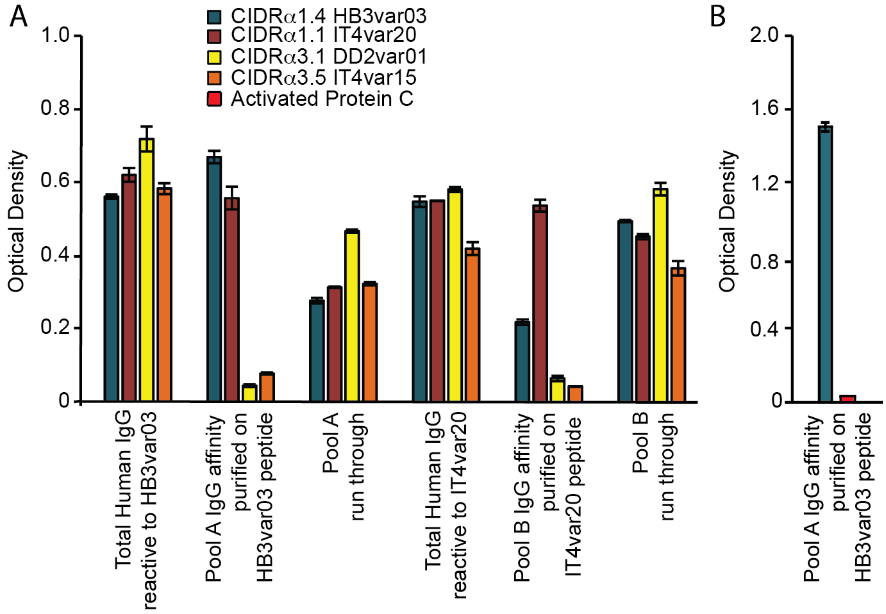
A
B
CIDRα1.4 HB3var03
CIDRα1.1 IT4var20
CIDRα3.1 DD2var01
CIDRα3.5 IT4var15
Activated Protein C
Optical Density
1.0
0.8
0.6
0.4
0.2
0
2.0
1.6
1.2
0.8
0.4
Total Human IgG reactive to HB3var03
Pool A IgG affinity purified on HB3var03 peptide
Pool A run through
Total Human IgG reactive to IT4var20
Pool B IgG affinity purified on IT4var20 peptide
Pool B run through

Figure S6

Table S1: Distribution and pairwise sequence similarity of the analysed CIDRα1 subclasses – related to Figure 1

Based on the maximum likelihood tree in Figure 1A we annotated the 885 CIDRα1 domains utilized for sequence variation analysis. [§]Redundancy was evaluated from the amino acid sequence. *Three small clusters of sequences of intermediate subtypes with highest resemblance to 1.4, 1.7 and 1.6a subtypes, respectively.

Subtype	Total obs.	Non-redundant sequences[§]	Sequence pair wise identity (%)		
			Minimum	Average	Median
CIDRα1.1	168 (19%)	124 (74%)	50	68	67
CIDRα1.2	86 (10%)	25 (29%)	57	94	95
CIDRα1.3	62 (7%)	12 (19%)	56	86	100
CIDRα1.4	130 (15%)	109 (84%)	40	59	57
CIDRα1.5a	74 (8%)	54 (73%)	45	66	64
CIDRα1.5b	69 (8%)	50 (72%)	40	63	65
CIDRα1.6a	33 (4%)	25 (76%)	58	81	83
CIDRα1.6b	41 (5%)	28 (68%)	54	70	67
CIDRα1.7	104 (12%)	75 (72%)	51	68	66
CIDRα1.8a	48 (5%)	41 (85%)	59	74	71
CIDRα1.8b	32 (4%)	24 (75%)	49	69	69
Unclassified*	38 (4%)	35 (92%)	-	-	-
All	885	602 (68%)	27	49	46

Table S2: Affinities of CIDRα1.1 domains for ECPR as measured by surface plasmon resonance – related to Figure 1

CIDR	Subclass	Range	K_D (nM)	k_{on} ($M^{-1}s^{-1}$)	k_{off} (s^{-1})
IT4var20	1.1	1uM-0.9nM	0.37	3.32×10^5	1.23×10^{-4}
IT4var19	1.1	0.5uM-0.5nM	16	2.27×10^4	3.64×10^{-4}
2110-3	1.1	1uM-0.9nM	32	1.57×10^4	5.11×10^{-4}
RAJ116var08	1.1	1uM-0.9nM	1.4	2.79×10^4	3.85×10^{-5}
ERS009959	1.1	8uM-7.8nM	182	3.89×10^2	7.24×10^{-5}
HB3var1csa	1.2	1uM-0.9nM	-	-	-
PFE1640w	1.3	1uM-0.9nM	-	-	-
HB3var03	1.4	1uM-0.9nM	0.37	5.32×10^5	1.97×10^{-4}
IT4var07	1.4	1uM-0.9nM	1.3	3.70×10^5	4.68×10^{-4}
DD2var32	1.4	1uM-0.9nM	0.3	1.06×10^6	3.43×10^{-4}
1965-2	1.5a	1uM-0.9nM	54	1.89×10^4	1.03×10^{-3}
ERS010323	1.5a	1uM-0.9nM	57	1.47×10^4	8.32×10^{-4}
1918-5	1.5b	8uM-7.8nM	229	4.71×10^2	1.08×10^{-4}
1983-13	1.5b	8uM-7.8nM	168700	2.8	4.70×10^{-4}
HB3var02	1.6a	8uM-7.8nM	1400	9.75×10^2	1.39×10^{-3}
PFD1235w	1.6a	1uM-0.9nM	8.8	7.55×10^3	6.62×10^{-5}
IT4var18	1.6b	2uM-1.8nM	56	5.62×10^3	5.63×10^{-8}
ERS010570	1.6b	1uM-0.9nM	15	3.51×10^4	5.31×10^{-4}
ERS010031	1.6b	1uM-0.9nM	8.8	3.70×10^4	3.28×10^{-4}
IT4var22	1.7	1uM-0.9nM	17	6.13×10^4	1.05×10^{-3}
1965-8	1.7	1uM-0.9nM	3.5	5.92×10^4	2.10×10^{-4}
ERS010178	1.8a	1uM-0.9nM	48	1.77×10^4	8.42×10^{-4}
MAL6P1.316	1.8a	1uM-0.9nM	50	1.67×10^4	8.28×10^{-4}
2054-3	1.8b	1uM-0.9nM	4	2.02×10^4	7.65×10^{-5}
1702-3	1.8b	1uM-0.9nM	6	3.64×10^3	2.28×10^{-5}
ERS010532	1.8b	1uM-0.9nM	24.18	6.55×10^3	1.58×10^{-4}

Table S3: Analysis of insertions and deletions in the CIDRα1.1 domains – related to Figure 1

Size polymorphisms are indicated with >< for deletions and <> for insertions in Figure 1B. Here the details of the type, prevalence and size of these polymorphisms.

Size variation (residues)	Average	Median
199-252	218.7	218

Position	Indel	Size	Prevalence	Associated subtype
515	Deletion	1	8%	1.6
532-534	Deletion	3	6.5%	1.7
549	Insertion	1-6	15.5%	1.4/1.5b
559	Deletion	1	10%	1.5b
566	Deletion	1	6%	1.5b
602	Deletion	1	8%	1.4/1.7
607-608	Insertion	1	95%	
640-641	Insertion	1	9%	1.5b
665	Insertion	1-3	82%	
		18-35	2.3%	1.8b
677	Deletion	1	7%	1.5b

Table S4: Summary of SAXS parameters of PfEMP1 domains, alone and in complex with EPCR – Related to Figure 2

The radius of gyration (R_g) was determined from the Guinier plot, and the maximum particle dimension (Dmax) and the Porod volume were calculated using GNOM. I_0 is an estimate of the scattering intensity at zero angle. An estimate of the molecular weight (Mw_{app}) was obtained by dividing the Porod volume by 1.7 and can be compared with the predicted molecular weight from sequence (Mw_{calc}), assuming a 1:1 complex.

	R_g (nm)	I_0	Volume (nm^3)	Mw_{app} (kDa)	Mw_{calc} (kDa)	D_{max} (nm)
HB3var03 CIDRα1.4	2.34	5643	42.6	25.0	26.1	6.5
EPCR	1.99	4727	38.9	22.9	20.0	5.5
HB3var03 CIDRα1.4:EPCR complex	2.63	9323	76.5	45.0	46.1	7.4
DD2var32 head (DBLα1.7-CIDRα1.4-DBLβ1	5.30	31700	263.9	155.2	140	18.7
DD2var32 head (DBLα1.7-CIDRα1.4-DBLβ1):EPCR complex	5.38	37550	309.0	181.8	160	18

Table S5: Crystallography data collection statistics – Related to Figure 2

	HB3var03 CIDRα1:EPCR			IT4var07 CIDRα1:EPCR		
Beamline	Diamond I-04			Diamond I-02		
Wavelength (Å)	0.98			0.9787		
Space Group	$C222_1$			$P3_221$		
Cell Parameters (Å)	a = 66.14, b = 94.72, c = 290.84; α = β = γ = 90			a = b = 56.02, c = 250.50; α = β = 90, γ = 120		
	Overall	Inner Shell	Outer Shell	Overall	Inner Shell	Outer Shell
Resolution (Å)	53.29-2.65	53.29-8.38	2.79-2.65	83.90-2.90	83.90-9.17	3.06-2.90
R_{mrg}	0.093	0.033	0.58	0.061	0.034	0.882
I/σ(I)	9.2	24.2	2.3	16.4	40	2.3
Completeness (%)	95.3	90.8	95.4	99.9	99.9	99.1
Multiplicity	3.6	3.5	3.6	8.4	7.1	6.9
Anomalous Completeness (%)	81.8	85.4	81.1	99.8	100	99
Anomalous Multiplicity	2.1	2	2	4.4	4.7	3.5

X-ray refinement statistics

	HB3var03 CIDRα1:EPCR	IT4var07 CIDRα1:EPCR
Resolution (Å)	2.65Å	2.90Å
Reflections used for refinement		
R_{work} (%)	22.2	24.7
R_{free} (%)	25.5	27.6
No. of protein residues in model	733	359
rmsd bond lengths (Å)	0.009	0.011
rmsd bond angles (°)	1.25	1.28
Ramachandran plot		
Allowed region	92.8	92.0
Additional allowed region	6.9	7.7
Generously allowed region	0.3	0.3
Disallowed region	0	0

Table S6: Affinities of mutants of the HB3var03 CIDRα1.4 domains for ECPR as measured by surface plasmon resonance – Related to Figure 3

Mutation	Range	K_D (nM)	k_{on} ($M^{-1}s^{-1}$)	k_{off} (s^{-1})
F651A	1uM-0.9nM	2.5	1.60×10^5	3.94×10^{-4}
F655L	1uM-0.9nM	4.3	1.45×10^5	6.30×10^{-4}
F655Y	1uM-0.9nM	0.5	1.10×10^6	5.72×10^{-4}
F656A	1uM-0.9nM	12.9	1.16×10^5	1.50×10^{-2}
F656V	1uM-0.9nM	1.5	1.45×10^5	2.23×10^{-4}
F656Y	1uM-0.9nM	1.9	3.29×10^5	3.79×10^{-4}
V658A	1uM-0.9nM	37	1.94×10^4	7.19×10^{-4}
W669S	1uM-0.9nM	11	4.01×10^4	4.49×10^{-4}
Q657K	1uM-0.9nM	72	6.89×10^4	4.99×10^{-3}
