# Supplementary material for: Structural Conservation Despite Huge Sequence Diversity Allows EPCR Binding by the PfEMP1 Family Implicated in Severe Childhood Malaria
Source: Cell Host Microbe. 2015 Jan 14;17(1):118–29. doi: 10.1016/j.chom.2014.11.007 (PMC4297295; doi:10.1016/j.chom.2014.11.007)
Supplement: Document S2. Article plus Supplemental Information [file mmc2.pdf]

Cell Host & Microbe
Article

Structural Conservation Despite Huge Sequence Diversity Allows EPCR Binding by the PfEMP1 Family Implicated in Severe Childhood Malaria

Clinton K.Y. Lau,[1,5] Louise Turner,[2,5] Jakob S. Jespersen,[2] Edward D. Lowe,[1] Bent Petersen,[3] Christian W. Wang,[2] Jens E.V. Petersen,[2] John Lusingu,[4] Thor G. Theander,[2] Thomas Lavstsen,[2,*] and Matthew K. Higgins[1,*]
[1]Department of Biochemistry, University of Oxford, South Parks Road, OX1 3QU Oxford, UK
[2]Centre for Medical Parasitology, Department of International Health, Immunology & Microbiology, University of Copenhagen and Department of Infectious Diseases, Rigshospitalet, 1017 Copenhagen, Denmark
[3]Center for Biological Sequence Analysis, Technical University of Denmark, 2800 Kgs. Lyngby, Denmark
[4]National Institute for Medical Research, Tanga, 2448 Ocean Road, P.O. Box 9653, Dar es Salaan, Tanzania
[5]Co-first author
*Correspondence: thomasl@sund.ku.dk (T.L.), matthew.higgins@bioch.ox.ac.uk (M.K.H.)
http://dx.doi.org/10.1016/j.chom.2014.11.007
This is an open access article under the CC BY license (http://creativecommons.org/licenses/by/3.0/).

SUMMARY

The PfEMP1 family of surface proteins is central for *Plasmodium falciparum* virulence and must retain the ability to bind to host receptors while also diversifying to aid immune evasion. The interaction between CIDRα1 domains of PfEMP1 and endothelial protein C receptor (EPCR) is associated with severe childhood malaria. We combine crystal structures of CIDRα1:EPCR complexes with analysis of 885 CIDRα1 sequences, showing that the EPCR-binding surfaces of CIDRα1 domains are conserved in shape and bonding potential, despite dramatic sequence diversity. Additionally, these domains mimic features of the natural EPCR ligand and can block this ligand interaction. Using peptides corresponding to the EPCR-binding region, antibodies can be purified from individuals in malaria-endemic regions that block EPCR binding of diverse CIDRα1 variants. This highlights the extent to which such a surface protein family can diversify while maintaining ligand-binding capacity and identifies features that should be mimicked in immunogens to prevent EPCR binding.

INTRODUCTION

Parasites, such as the *Plasmodium* species that cause malaria, have developed strategies to aid survival in a mammalian host and to multiply in the nutrient-rich blood. They must make specific interactions with host molecules, enabling them to invade cells, acquire nutrients, and populate protected environments. At the same time, they must avoid detection by components of the innate and acquired immune systems. A common evolutionary strategy, employed by many unicellular eukaryotic parasites, is expansive development of a family of surface proteins, which lie at the interface between host and parasite. Examples include PfEMP1 (Leech et al., 1984), RIFIN (Kyes et al., 1999), and STEVOR (Cheng et al., 1998) of *Plasmodium falciparum*, VIR of *P. vivax* (del Portillo et al., 2001), variant surface glycoproteins (VSGs) of *Trypanosoma brucei* (Schwede and Carrington, 2010), MASP (El-Sayed et al., 2005) and SAP (Carmo et al., 2001) of *Trypanosoma cruzi*, and SAGs of *Toxoplasma gondii* (Kasper et al., 1983). Expression switching between family members allows parasites to display a series of antigenically distinct surfaces, posing challenges for the immune system and for rational development of vaccines.

The PfEMP1 protein family of *Plasmodium falciparum* is one of the most closely studied surface protein families, with about 60 members encoded in each genome (Smith et al., 2013; Gardner et al., 2002). They are expressed on the surfaces of infected erythrocytes where they interact with various human endothelial receptors, tethering these erythrocytes to blood vessel or tissue surfaces. This prevents spleen-mediated clearance of the parasite and allows the infection to build. It also leads to the most severe symptoms of the disease, resulting in inflammation of the brain and the placenta during cerebral or pregnancy-associated malaria (Miller et al., 2002). PfEMP1 are therefore under dual selection pressure to retain the ability to bind to the vasculature while diversifying into a family of antigenically distinct proteins.

The extracellular ectodomains of the PfEMP1 proteins contain 2–10 copies of two *Plasmodium*-specific domain types, the Duffy-binding-like (DBL) and cysteine-rich interdomain region (CIDR) domains (Baruch et al., 1995; Smith et al., 1995; Su et al., 1995; Gardner et al., 2002). Individual domains frequently act as discrete ligand-binding modules, with a diverse set of host endothelial surface proteins and carbohydrates identified as partners for different domains (Smith et al., 2013). DBL and CIDR domains have been divided into specific classes based on sequence similarity and the presence of constituent homology blocks (Smith et al., 2000; Rask et al., 2010). Specific domain subclasses interact with specific endothelial receptors (Smith et al., 2000). However, even within a domain subclass, sequence diversity is high, making it challenging to identify conserved functional regions required to mediate binding to a particular receptor based on sequence analysis (Robinson et al., 2003; Howell et al., 2008; Higgins and Carrington, 2014).

Despite significant PfEMP1 sequence diversity, natural immunity to severe malaria is acquired after only one or two severe infections, and immunoglobulin G (IgG) that binds PfEMP1 and prevents adhesion plays a significant role (Bull et al., 1998; Salanti et al., 2004; Lusingu et al., 2006; Cham et al., 2009; Gupta et al., 1999; Nielsen et al., 2002; Gonçalves et al., 2014). This raises hope that it will be possible to develop a vaccine to mimic this natural immunity and to prevent severe disease. However, such a vaccine must raise antibodies that recognize a diverse set of PfEMP1 proteins, and rational design of constituent immunogens requires an understanding of this diversity and detailed knowledge of the structures of conserved features that should be targeted by inhibitory antibodies. The lack of a structure of a PfEMP1 protein domain in complex with a protein ligand has made such an analysis impossible.

In this study, we have combined sequence analysis with structural and biochemical studies to determine the extent to which PfEMP1 domains that interact with a particular receptor can diversify and to identify features that remain conserved. We have focused on the interaction between CIDRα1 domains and endothelial protein C receptor (EPCR), as the expression of CIDRα1-containing PfEMP1s and the EPCR-binding phenotype are both associated with severe childhood malaria (Lavstsen et al., 2012; Turner et al., 2013). Indeed, the key role of this interaction in malaria pathogenesis is substantiated by the discovery of altered brain endothelial EPCR expression in cerebral malaria patients (Moxon et al., 2013). Additionally, a polymorphism in the transmembrane domain of EPCR that leads to increased plasma levels of soluble receptor also associates with protection from severe malaria in a Thai population (Naka et al., 2014). Here, we show that EPCR-binding CIDRα1 domains are extremely diverse, even in the residues that directly contact EPCR. However, we find that conserved structural features with conserved bonding potential are retained to maintain this binding phenotype. This shows the extent to which such a parasite protein family can diversify while retaining high-affinity ligand binding and characterizes the features that should be targeted in development of therapeutics to block EPCR binding in severe malaria.

RESULTS

Extensive Sequence Diversity among EPCR-Binding CIDRα1 Domains

Endothelial protein C receptor binding was identified as a property of CIDRα1 domain variants found in PfEMP1 proteins containing two particular combinations of domains: domain cassette 8 (DBLα2-CIDRα1.1-DBLβ12-DBLγ4/γ6) and domain cassette 13 (DBLα1.7-CIDRα1.4) (Turner et al., 2013). These cassettes are present in PfEMP1s expressed in a large proportion of tested children suffering from severe malaria (Lavstsen et al., 2012; Bertin et al., 2013) and also in parasites selected for adhesion to brain endothelial cells (Avril et al., 2012; Claessens et al., 2012), suggesting a pivotal role in severe outcomes of *P. falciparum* infections. EPCR binding by PfEMP1s was mapped exclusively to their CIDRα1.1 and CIDRα1.4 domains. Indeed, the CIDRα1.1 domain of the IT4var20 PfEMP1 protein bound to EPCR with an affinity comparable to that of the whole ectodomain (Turner et al., 2013). Other CIDR domain classes, not present in DC8 and DC13 domain cassettes, such as the CIDRα2 and CIDRα3 domains, did not interact with EPCR but bound to CD36 (Turner et al., 2013).

To test the depth of diversity of EPCR-binding domains, we expanded our collection of CIDRα1 domain sequences from the previously described 66 sequences, originating mainly from seven parasite genomes (Kraemer and Smith, 2006; Rask et al., 2010), by addition of domain sequences extracted from assemblies of whole-genome sequencing data from 226 parasite isolates collected in both Africa and Asia (Manske et al., 2012), resulting in a total data set of 885 sequences. These domains were grouped, based on phylogenetic analysis, into eight previously defined subclasses (CIDRα1.1–1.8) with an additional splitting of CIDRα1.5, CIDRα1.6, and CIDRα1.8 variants into two, generating CIDRα1.5a/b, CIDRα1.6a/b, and CIDRα1.8a/b (Figure 1A; Table S1, available online). To determine which subclasses contain features required to bind EPCR, members of each subclass, chosen to represent the diversity across CIDRα1 domains, were produced in an insect cell system and tested for binding to EPCR and CD36 by ELISA. All proteins bound to EPCR, with the exception of CIDRα1.2 and CIDRα1.3 domains, which are both found in *var1* genes considered to be pseudogenes (Figures 1A and S1A).

Binding was further characterized by surface plasmon resonance (SPR), allowing determination of binding affinities and kinetic constants. We developed an SPR assay in which EPCR was produced with an N-terminal biotin, allowing coupling to a chip with an orientation matching that found on the cell surface and allowing complete regeneration between measurements. This was used to show that all members of subclasses CIDRα1.1 and CIDRα1.4–1.8 bound to EPCR. The majority of domains bound with high affinities in the range of 0.3–60 nM, but with a few weaker binders (Figures 1A and S1B, Table S2). Despite differences in affinity, it was noticeable that all domains bound with a slow off rate. Indeed, kinetic analysis showed less variation in rate constants for dissociation than in those for association, with a propensity toward slow off rates (Figure S1; Table S2), suggesting that these domains are under selection pressure to form a stable complex with EPCR.

To better understand the degree of diversity of EPCR-binding domains, we analyzed 737 different CIDRα1 sequences from members of the six EPCR-binding subclasses (CIDRα1.1 and CIDRα1.4–1.8). These showed little identity between variants, with just 14 residues (6.5%) absolutely conserved and a further 22 residues conserved in more than 90% of the domains (Figure 1B; Table S3). Most conserved residues are cysteines or aromatics. This is reminiscent of the PfEMP1 DBL domains in which the small percentage of conserved cysteine and aromatic residues are found in the domain core where they play a structural role (Batchelor et al., 2011, 2014; Higgins, 2008; Higgins and Carrington, 2014; Hodder et al., 2012; Khunrae et al., 2010; Lin et al., 2012; Malpede et al., 2013; Singh et al., 2006; Tolia et al., 2005; Vigan-Womas et al., 2012). All residues conserved in the EPCR-binding domains are also totally conserved in CIDRα1.2 and CIDRα1.3 subclasses that do not bind EPCR, showing that CIDRα1 domains are an extremely diverse subfamily that lacks conserved residues that correlate with EPCR binding. We therefore determined cocrystal structures to allow us to understand the molecular basis for EPCR

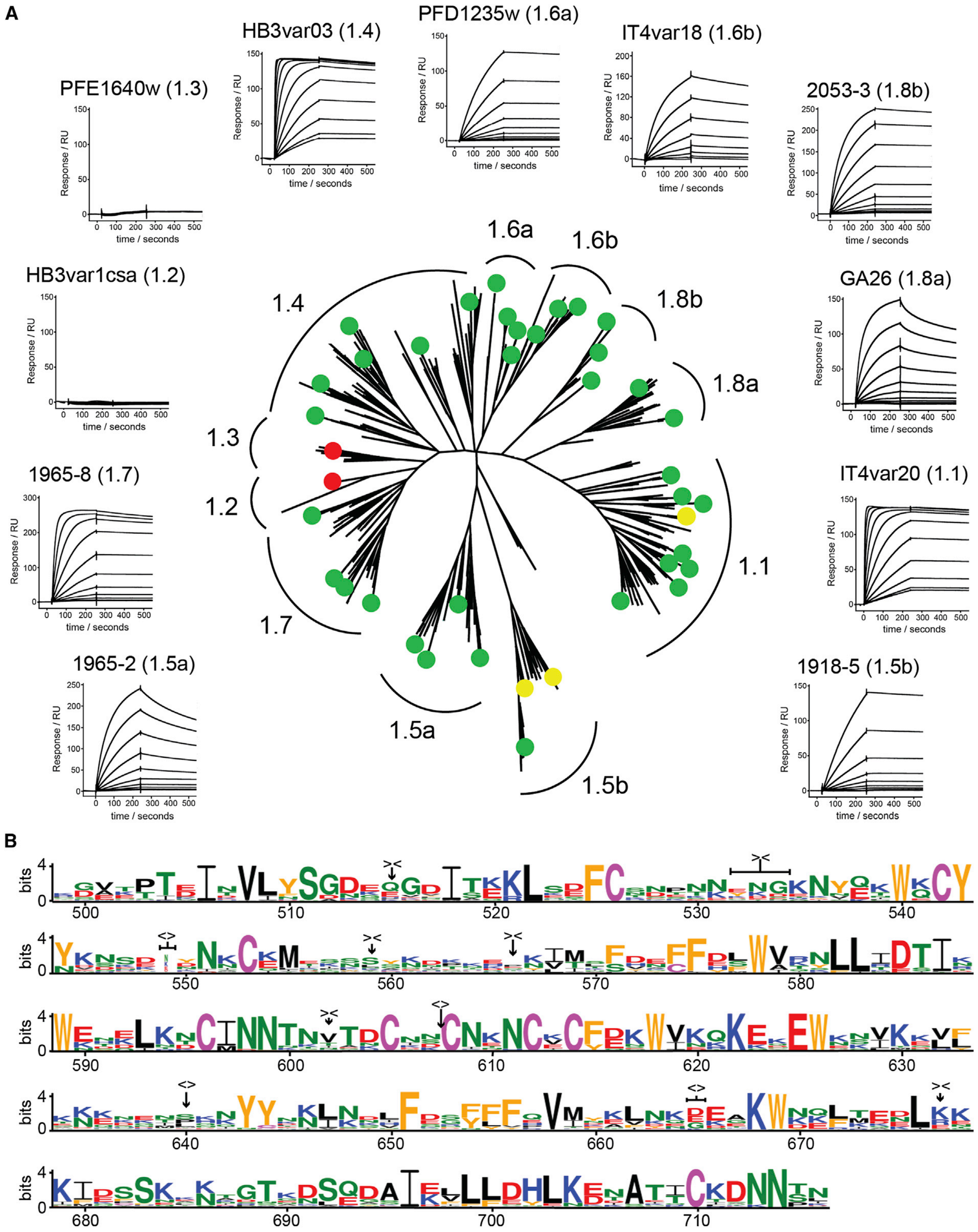

Figure 1. High Sequence Diversity in EPCR-Binding CIDRα1 Domains

(A) A maximum likelihood tree (bootstrap n = 50) of 885 full-length CIDRα1 domains used in this study showing branching into previously identified subclasses CIDRα1.1–1.8 and the bipartition of subclasses CIDRα1.5, CIDRα1.6, and CIDRα1.8. Circles represent the degree of EPCR binding by ELISA with positive (green), negative (red), and weakly positive (yellow). Also shown are representative SPR traces for each CIDRα1 subclass showing binding to EPCR.

(B) All sequences of CIDRα1 subclasses 1.1 and 1.4–1.8 were aligned, and a sequence logo was generated of residues equivalent to those found in the HB3var03 CIDRα1 domain (numbered as in HB3var03). Deletions (> <) and insertions (< >) are indicated as explained in Table S3. See also Figure S1.

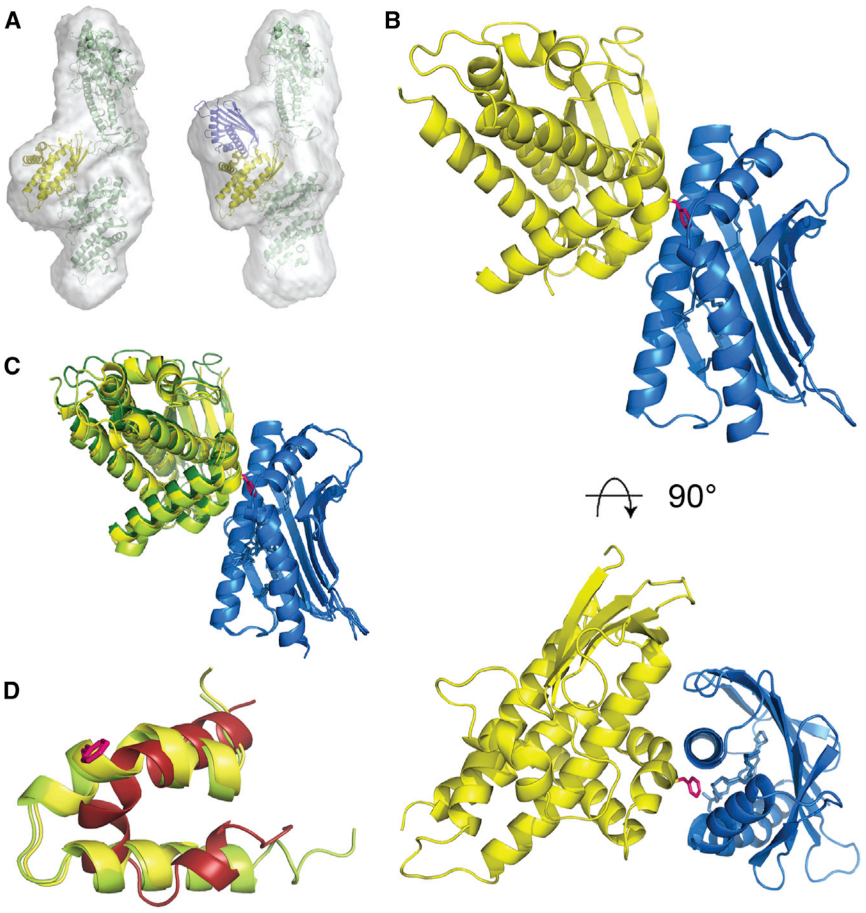

Figure 2. The Structure of the CIDRα1:EPCR Complex

(A) Molecular envelopes derived from small-angle X-ray scattering for DD2var32 domains DBLα1.7-CIDRα1.4-DBLβ1 without (left) and with (right) EPCR.

(B) Structure of a complex of the HB3var03 CIDRα1 domain (yellow) bound to EPCR (blue). F656 of the CIDRα1 domain is shown as pink sticks.

(C) Structural overlay of complexes of EPCR with IT4var07 CIDRα1 (pale green) and the two copies of HB3var03 CIDRα1 (yellow and dark green) found in the crystal.

(D) A close up of the EPCR-binding region of HB3var03 CIDRα1 (yellow), IT4var07 CIDRα1 (green), and the equivalent region of var0 CIDRγ (red) reveals the different architecture of the CIDRα1 domains in this region. See also Figure S2.

binding and to rationalize how sequence diversity is compatible with the retention of this binding phenotype.

The Structural Basis for EPCR Binding by PfEMP1s

We have previously shown that a single CIDRα1 domain binds to EPCR with the same affinity as the full-length PfEMP1 protein, demonstrating that EPCR binding capability is contained entirely within CIDRα1 (Turner et al., 2013). Our strategy here was to select a diverse set of domains, increasing the likelihood of identifying a complex that would crystallize, and then combine the structures we obtained with sequence analysis and biophysical studies to rationalize EPCR binding by the protein family. We therefore generated a panel of CIDRα1 domains with domain boundaries appropriate for crystallization and used SPR and isothermal titration calorimetry (ITC) to confirm binding to EPCR with nanomolar affinities and slow dissociation rates (Figures S1 and S2). These domains were reconstituted into complexes with the extracellular domain of EPCR and examined using small-angle X-ray scattering, analytical ultracentrifugation, and multi-angle laser light scattering. In each case, a 1:1 complex formed with no higher-order assemblies observed (Figure S2). Small-angle X-ray scattering of a protein containing the three membrane distal domains of DD2var32 (DBLα1.7-CIDRα1.4-DBLβ1), alone and in complex with EPCR, also revealed the formation of a 1:1 PfEMP1:EPCR complex (Table S4). In addition, molecular envelopes showed a predominantly elongated architecture for these three domains (Figure 2A). This architecture did not alter in the presence of EPCR, but instead a single additional protein density was evident, attached to the central CIDRα1 domain (Figure 2A), supporting the notion of a modular arrangement for the PfEMP1 protein with a single EPCR binding site on the CIDRα1 domain.

The CIDRα1:EPCR complexes were next subjected to crystallization trials. Crystals of the HB3var03 CIDRα1.4:EPCR complex formed and diffracted to 2.65Å resolution. The structure was determined by molecular replacement using the structure of EPCR (PDB 1L8J) as a search model, followed by iterative model building and refinement (Figures 2B and S3; Table S5). The structure was consistent with an envelope obtained from solution small-angle X-ray scattering (Figures S2D–S2F), and the two copies of HB3var03 CIDRα1 in the asymmetric unit aligned with a root-mean-square deviation (rmsd) of just 0.09Å, showing them to be extremely similar.

A second complex, containing IT4var07 CIDRα1.4:EPCR complex also crystallized, and crystals diffracted to 2.9Å resolution. Despite a sequence identity of 78.5% compared to the HB3var03 CIDRα1 domain, this complex crystallized in a different space group and with different crystal packing. The structure was determined using HB3var03 CIDRα1 and EPCR as separate search models in molecular replacement. HB3var03 and IT4var07 CIDRα1 are extremely similar (rmsd = ∼0.3Å), but despite differences in space groups and crystal packing, both CIDRα1 domains bind to EPCR using the equivalent surface (Figure 2C).

The two CIDRα1 domains are built around a long three-helical core bundle. On one side of this bundle lies a four-stranded β sheet. On the opposite side, between the second and third core helices, an insertion folds into a kinked α helix and a long α helix that lie approximately perpendicular to the core bundle, stabilized by residues F651, V658, and W669 and forming the majority of the EPCR-binding surface (Figure 3A). The α-helical

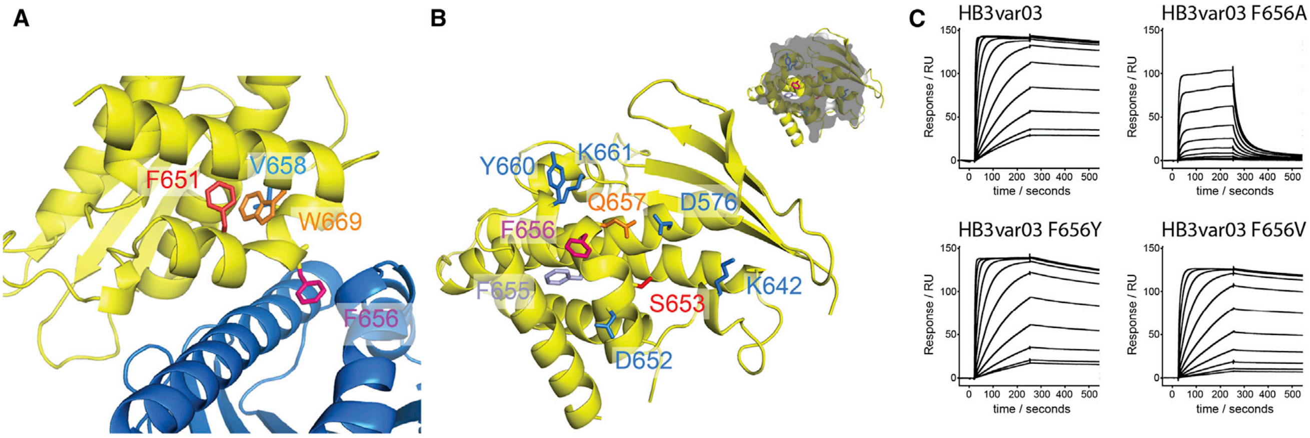

Figure 3. The Architecture of the EPCR Binding Site

(A) A close up of the EPCR binding site with HB3var03 CIDRα1 (yellow) and EPCR (blue). Three residues (F651, V658, and W669) that lie beneath the kinked helix are labeled. This kink causes F656 to protrude and interact with the hydrophobic groove of EPCR.

(B) Structure of the EPCR-binding surface of HB3var03 CIDRα1. Residues shown as sticks directly interact with EPCR. The inset shows a view of the HB3var03 CIDRα1 domain in the same orientation with a gray cross-section of EPCR chosen to show F656 protruding into the hydrophobic groove of EPCR.

(C) SPR data showing binding of HB3var03 and its F656A, F656V, and F656Y mutants to EPCR. See also Figures S3 and S4.

core of the CIDRα1 domain, and the EPCR-binding surface, are well ordered and well defined in the crystal structure, with B factors of 20–40 (Figures S2I and S2J). However, away from the binding surface, the domain is decorated with a variety of loops, some of which are not observed in the electron density, while others are characterized by high B factors, suggesting flexibility. Comparison with the two existing CIDR domain structures shows the CIDRα1 domains to be more similar to CIDRγ from var0 (Vigan-Womas et al., 2012) (Figure 2D) than to CIDRα2 from CD36-binding MC179 (Klein et al., 2008), with the most significant structural differences in the EPCR-binding region. This region is part of a homology block (HB121) that is unique to CIDRα1 domains (Rask et al., 2010).

The HB3var03 CIDRα1:EPCR interface has a surface area of 978Å^2 and surrounds the kinked helix (Figure 3A). At its center is a small hydrophobic patch containing F656 and F655, which interact with a hydrophobic patch on EPCR. In particular, F656 is positioned at the bend in the kinked α helix, where it protrudes from the domain surface, allowing it to insert into the hydrophobic groove of EPCR (Figures 2B and 3A). This patch lies within a larger surface that is complementary in shape to EPCR and contains a series of amino acids (D576, K642, D652, S653, Q657, Y660, and K661) that make hydrogen bonds to EPCR side chains (Figures 3B and 4).

Both the hydrophobic contacts and the hydrogen bonds play an important role in the interaction. ITC measurements show that binding is driven by a negative enthalpy change (Figure S2A), most likely due to formation of hydrogen bonds. However, insertion of F656 into the hydrophobic groove of EPCR is also important for complex stability. Mutation of F656 to hydrophobic residues, tyrosine or valine, reduced the affinity by only ~4-fold. However, the F656A mutation, which removes the majority of this interaction, had a 35-fold effect on the affinity and significantly increased the off rate, with a 100-fold change in the dissociation rate constant (Figures 3C and S4; Table S6). Therefore, the insertion of F656 into the hydrophobic groove of EPCR increases the lifetime of the complex. In combination with hydrogen bond formation, this leads to a high affinity and stable interaction, as required to hold infected erythrocytes in place against the buffeting of blood flow.

PfEMP1 and the Natural Ligand Protein C Share an Overlapping Binding Site on EPCR

We previously demonstrated that preincubation of EPCR with CIDRα1 domains prevents the interaction with its natural ligand, protein C, suggesting that infected erythrocytes might interfere with EPCR-mediated signaling in severe malaria (Turner et al., 2013). Indeed, comparison of the structures of the CIDRα1: EPCR complexes with that of EPCR bound to the Gla domain of activated protein C (Oganesyan et al., 2002) reveals significant overlap, with both protein C and CIDRα1 domains interacting with the same region of the hydrophobic groove of EPCR (Figure 5).

Most of the interactions between protein C and EPCR (718Å^2 of the total 771Å^2 interaction surface) are mediated through a hydrophobic loop from protein C that positions residues F4 and L5 to bind into the hydrophobic groove of EPCR. In particular, F4 binds to the same site as F656 from HB3var03 CIDRα1 (Figure 5). The overlapping binding sites of protein C and CIDRα1 domains will likely cause sequestered infected erythrocytes to inhibit the binding of activated protein C. It is conceivable that evolution has driven PfEMP1 to interact with this functionally important ligand-binding site of EPCR to reduce the likelihood of host mutations occurring in the interface that would disrupt PfEMP1 binding.

Although the binding sites overlap, there are also significant differences. The CIDRα1-binding site is larger than that of protein C, extending to make contacts with two additional EPCR loops (residues 22–25 and 44–47) through K642 of the HB3var03 CIDRα1 domain. In contrast, protein C makes strong electrostatic interactions through an associated calcium ion with E86 of EPCR, a residue that plays little role in the interaction with

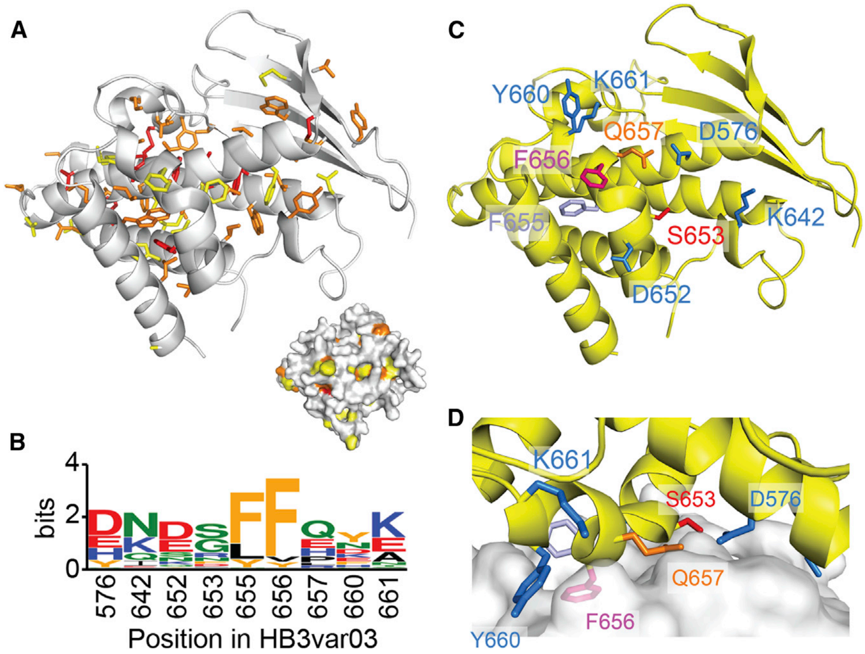

Figure 4. Diversity and Conservation in the CIDRα1 Domains

(A) The 14 completely conserved residues in CIDRα1 domains, shown as red sticks on the HB3var03 CIDRα1 structure. Residues with a property entropy score of less than 0.2 (but not totally conserved) are orange, and those with scores of 0.2–0.3 are yellow. The inset shows a surface representation in the same orientation and colors, showing that conserved residues cluster in the domain center.

(B) A sequence logo showing variation in CIDRα1 residues that directly contact EPCR.

(C and D) Structure of the EPCR-binding surface of the HB3var03 CIDRα1 domain. Residues shown as sticks make direct interactions with EPCR. See also Figure S5.

CIDRα1 domains. Therefore, although protein C and CIDRα1 domains overlap, there are differences in their binding sites, which may be exploitable in the development of therapeutic EPCR variants or compounds that interact with just protein C or PfEMP1.

Structural Conservation and Surface Diversity of the EPCR-Binding CIDRα1 Domains

Having identified residues and structural features used by the HB3var03 and IT4var07 CIDRα1 domains to bind to EPCR, we next assessed the degree of conservation of these features in 737 CIDRα sequences from the EPCR-binding CIDRα1 subclasses. As predicted, the 14 totally conserved residues were all located in the core of the domain, with aromatic residues packing together at the interfaces between the helices and cysteines forming disulphide bonds to stabilize the structure (Figure 4A).

With so little absolute sequence conservation, we next assessed the degree to which residues varied while retaining their chemical property. The aligned sequences were interrogated at each amino acid position, using the Shannon entropy of physiochemical property method (Capra and Singh, 2007; Mirny and Shakhnovich, 1999), and scores were plotted onto the HB3var03 CIDRα1 structure. Side chains showing significant conservation of surface property (a score of <0.3) are also mostly internal and are likely to contribute to the fold (Figure 4A). These include aromatic residues (F651, V658, and W669 in HB3var03) that lie between the core bundle and binding helix and contribute to the formation of the kink and arrangement of residues critical for binding (Figure 3A). Indeed, single point mutations of these residues, designed to disturb these structural features, reduced EPCR binding affinity by up to 100-fold (Figure S4; Table S6). Therefore, as seen in the DBL domains (Higgins and Carrington, 2014), most conserved residues in CIDRα1 are internal, most likely stabilizing the domain architecture and correctly positioning surface residues for ligand binding.

In contrast, surfaces of CIDRα1 domains, which are under immune selection pressure to diversify, vary significantly. This extends even to residues that make direct contacts with EPCR. Here, the most significant conservation is at positions occupied by F655 and F656 in HB3var03 (Figures 4B–4D and S5). While phenylalanine is most common in both positions, other aromatic or hydrophobic side chains, such as tyrosine, leucine, or valine, are also observed. These relatively conservative changes retain the hydrophobic nature of the protrusion, maintaining its capacity to insert into the hydrophobic groove of EPCR. Indeed, the F655L, F655Y, F656Y, and F656V mutants of HB3var03 all bind to EPCR with affinities of less than 5 nM (Figure S4; Table S6). The other residues that interact with EPCR, through hydrogen bonding interactions, are more divergent. However, at each of these positions, the most common substitution is with another amino acid that can form hydrogen bonds (Figure 4).

Interestingly, the CIDRα1.2 and CIDRα1.3 subclasses, shown not to bind EPCR, contain residues in these nine positions consistent with binding. However, they also share a lysine at the position equivalent to Q657 in HB3var03, a change that is not observed in other subclasses and that places a positive charge next to an arginine in EPCR, most likely leading to repulsion. Indeed, the Q657K mutation of HB3var03 leads to an ~200-fold reduction in EPCR binding affinity and a dramatic increase in the off rates (Figure S4; Table S6), suggesting that this change makes a significant contribution to the lack of EPCR binding by these subclasses.

Therefore, the EPCR binding surface shows significant sequence variability but retains its structure through conservation of core residues. It also retains the essential chemical nature of its surface residues through retention of a hydrophobic protrusion and a surrounding network of hydrogen bond donors and acceptors.

Patient Sera Contain Antibodies that Disrupt EPCR Binding with Cross-Inhibitory Potential

The high surface sequence variability of CIDRα1s, driven by the selection pressure to avoid immune detection, will reduce the

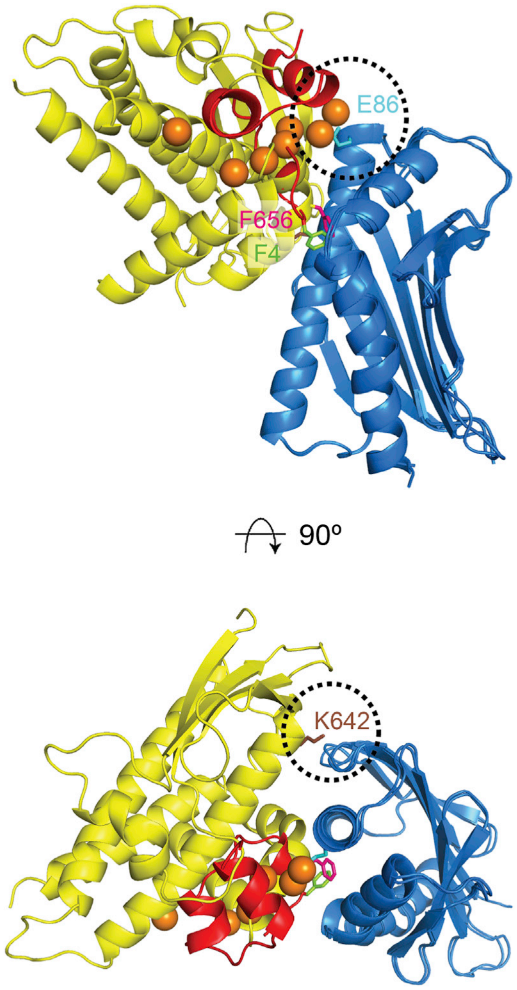

Figure 5. The CIDRα1 Domains Overlap the Protein C Binding Site on EPCR

Structure of a complex of the CIDRα1 domain of HB3var03 (yellow) bound to EPCR (blue), overlaid with that of the Gla domain of activated protein C (red) bound to EPCR. Calcium ions in the Gla domain are shown as orange spheres. Residues F4 of protein C (green) and F656 of HB3var03 CIDRα1 (pink) bind the same pocket of EPCR. Residue E86 of EPCR (cyan) interacts with the calcium ions of protein C, forming a binding surface largely unused by CIDRα1. Loops of CIDRα1 domain, including K642 in HB3var03 (brown), interact with loops from EPCR not contacted by protein C.

likelihood of acquisition of cross-reactive antibodies that prevent EPCR binding and erythrocyte sequestration. However, despite this diversity, natural immunity to severe malaria is acquired after only one or two severe infections and involves IgG that target PfEMP1 (Bull et al., 1998; Salanti et al., 2004; Lusingu et al., 2006; Cham et al., 2009; Gupta et al., 1999; Nielsen et al., 2002; Gonçalves et al., 2014). We therefore investigated whether individuals (aged 4–15 years) from a malaria-endemic region of Tanzania had acquired antibodies that bind recombinant HB3var03 CIDRα1.4 and IT4var20 CIDRα1.1 domains and to what extent these antibodies can prevent EPCR binding by a diverse set of CIDRα1 domains. HB3var03 CIDRα1.4 and IT4var20 CIDRα1.1 domains share only four out of nine residues that contact EPCR and were selected to represent the two major divergent groups of CIDRα1 domains (see Figure 1A), the CIDRα1.2–CIDRα1.7 domains and the CIDRα1.1/CIDRα1.8 domains, encoded by *var* genes controlled by UPSA and UPSB promoters, respectively (Lavstsen et al., 2003; Sander et al., 2014). We found that a large fraction of tested individuals had acquired IgG capable of inhibiting EPCR binding by HB3var03 CIDRα1.4 or IT4var20 CIDRα1.1 (41/45 and 51/76, respectively).

Next, to assess the cross-inhibitory potential of these antibodies, plasma from individuals with IgG reactive to HB3var03 were pooled, and IgG was affinity purified using a synthetic peptide containing the EPCR-binding region of HB3var03 (generating IgG pool A). The same procedure was used for IT4var20 reactive sera using a binding-site peptide from IT4var20 (IgG pool B). Despite being purified on different peptides, both IgG preparations reacted in ELISA with both HB3var03 and IT4var20 CIDRα1 domains, but not with CD36 binding CIDRα3 control domains (Figure S6). In addition, at 50 μg/ml IgG concentration, both IgG pool A and IgG pool B showed almost complete inhibition of the binding of both IT4var20 and HB3var03 CIDRα1 domains to EPCR (Figure 6A). We also tested the ability of pool A IgG to recognize activated protein C and saw no cross reactivity, indicating that these IgG will not affect the EPCR:APC interactions (Figure S6B). Indeed, although CIDRα1 domains and APC share an overlapping binding site on EPCR (Figure 5), they are structurally very different, making it unlikely that antibodies raised against CIDRα1 domains will cross-react with APC.

To assess the degree to which these IgG cross-inhibit EPCR binding, they were tested against a set of 25 EPCR binding CIDRα1 domains, selected to represent the breadth of sequence diversity. At a lower concentration of 20 μg/ml, pool A IgG reduced EPCR binding by most of the UPSA CDIRα1 domains tested and had a significantly lower effect on CIDRα1 variants from UPSB. Conversely, pool B IgG reduced EPCR binding by many of the UPSB CIDRα1.1 domains and showed statistically significant lower reduction of binding by UPSA domains (Figures 6B and 6C).

Finally, we tested whether the purified IgG could also block endothelial cell binding by parasite-infected erythrocytes expressing IT4var20, a PfEMP1 shown to bind only to EPCR (Turner et al., 2013). Both pool A and pool B IgG inhibited this binding to the same extent as soluble EPCR or IgG raised against the CIDRα1.1 domain of IT4var20 (Figure 7). These data show that individuals living in malaria-endemic areas acquire functional antibodies that target the ligand-binding region of CIDRα1 through natural infection. These antibodies have the capacity to block both EPCR binding of CIDRα1 domains and endothelial cell binding by parasite-infected erythrocytes. They also show some cross-inhibitory potential, with IgG affinity purified on the EPCR-binding region of one CIDRα1 domain able to reduce EPCR binding by other, diverse CIDRα1 domains.

DISCUSSION

Parasites frequently express surface protein families that lie at the interface between pathogen and host, experiencing the selection pressure to diversify to avoid immune detection while maintaining conserved features required for their function in host-parasite interactions. The roles of these protein families vary significantly. Some, such as the Trypanosome variant

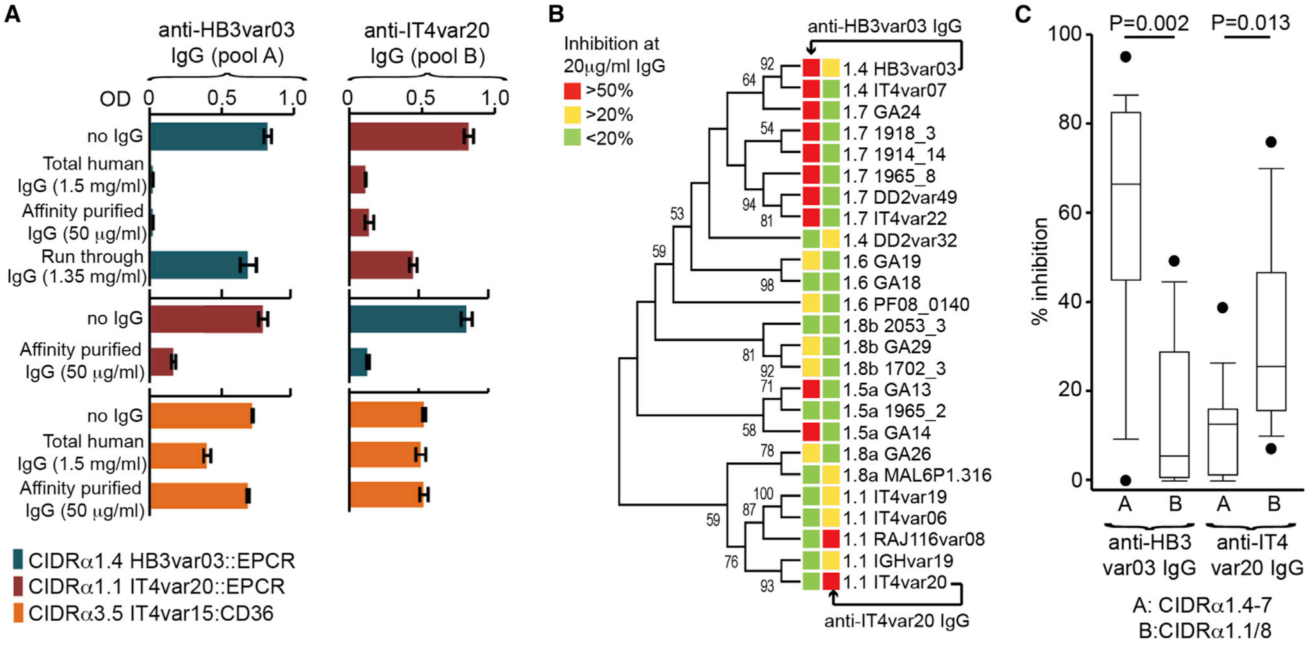

Figure 6. Human Sera Contain Antibodies that Block the CIDRα1:EPCR Interaction

(A) Human IgG preparations from Tanzanian individuals inhibit ELISA binding of HB3var03 CIDRα1.4 or IT4var20 CIDRα1.1 to EPCR, but not the binding of CIDRα3.5 to CD36. Antibodies tested included total human IgG reactive to the CIDRα1 domain under study (Total human IgG), IgG affinity purified using a peptide covering the EPCR-binding site of the CIDRα1 domain under study (Affinity purified IgG), and IgG that did not bind to this affinity column (Run through IgG). This was done for a UPSA PfEMP1 (HB3var03, pool A) and a UPSB PfEMP1 (IT4var20, pool B).

(B) Inhibition of binding of 25 CIDRα1 domains to EPCR by two peptide affinity-purified human IgG preparations (anti-HB3var03 and anti-IT4var20 IgG). The sequence similarity of the region corresponding to the peptide sequence of the 25 domains is shown by the maximum likelihood tree. The level of binding inhibition of each CIDRα1 domain to EPCR by the IgG preparations is shown by color-coded boxes plotted on the tree.

(C) A summary of the percentage of EPCR binding inhibition (median and 10th, 25th, 75th, and 90th percentiles) of each affinity purified IgG preparation on CIDRα1 domains belonging to UPSA or UPSB (rank-sum p values) shows the greatest cross-inhibition of UPSA domains by UPSA-purified sera and of UPSB domains by UPSB-purified sera. See also Figure S6.

surface glycoproteins (VSGs), form a structural coat, protecting the parasite surface underneath, but have no known human ligands (Schwede and Carrington, 2010). In contrast, the PfEMP1 proteins from *Plasmodium falciparum* have a more complex function with the requirement to retain host receptor binding (Smith et al., 2013). This imposes additional constraints on protein family evolution, raising questions about the degree to which such a protein family can diversify while retaining host interaction capability. It also raises questions for those engaged in vaccine development about whether features found on the protein surface are sufficiently conserved to allow the immunogen-mediated induction of a broadly neutralizing immune response.

In this study we provide the structure of a module from a divergent parasite-expressed surface protein family in complex with its host ligand. This reveals the structural features that the CIDRα1 domains from the PfEMP1 proteins have evolved in order to interact with EPCR. In particular, we see the acquisition of a loop between the second and core third helices and the folding of this loop into a platform on which EPCR docks. We see the presence of a kinked helix, promoting the protrusion of a hydrophobic residue into the hydrophobic groove of EPCR, mimicking F4 from the natural ligand of EPCR, protein C. We also see a surface decorated with hydrogen bond donors and acceptors that makes further interactions with EPCR and stabilizes the complex.

This structure provides us with the framework to understand analysis of 737 sequences of EPCR-binding CIDRα1 domains. It reveals that most conserved residues are found in the interior of the domain, with conserved disulphide bonds stabilizing the fold, and conserved aromatic residues facilitating helical packing, as in the related DBL domains (Higgins and Carrington, 2014). Conserved aromatic residues also stabilize the kinked architecture of the EPCR-binding loop, making it extremely likely that this is conserved across all CIDRα1 variants.

In contrast, surface sequence conservation is extremely low. Even residues that directly contact EPCR exhibit significant diversity. However, the potential for bond formation is largely retained, with the hydrophobic protrusion remaining hydrophobic in the large majority of CIDRα1 domains, and residues with hydrogen bonding potential are largely replaced with other hydrogen bond donors and acceptors. Therefore the CIDRα1 domains appear to retain a conserved architecture but with extensive surface divergence. However, the EPCR-binding surface retains sufficient chemical similarity to allow retention of the capacity to form a stable complex with EPCR.

This study focuses on the interaction of PfEMP1 with one host receptor. However, we expect other such surface protein families to follow similar principles. As we see here for EPCR, it has been challenging, or impossible, to find conserved residues on PfEMP1 domains that interact with other host receptors, such

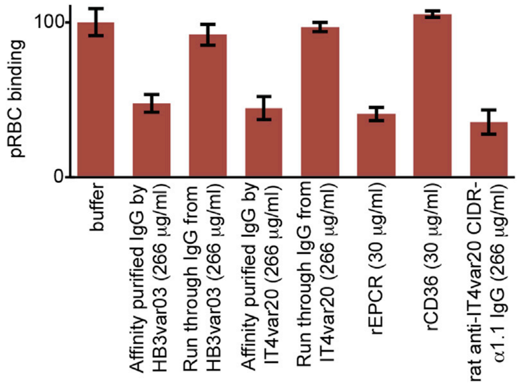

Figure 7. Human Sera Contain Antibodies that Block Infected Erythrocytes from Binding to EPCR

Binding inhibition of parasite-infected erythrocytes expressing native IT4var20 (pRBC) to HBMECs by IgG preparations (Affinity purified IgG by either HB3var03 CIDRα1 or IT4var20 CIDRα1 peptides), control IgG (Run through IgG from either HB3var03 or IT4var20), soluble recombinant EPCR and CD36, and IgG from a rat immunized with IT4var20 CIDRα1.

as ICAM-1 or CD36, by sequence analysis alone (Robinson et al., 2003; Howell et al., 2008). With immune pressure driving surface variation to extremes, it is likely that retention of conserved domain architecture and surface interaction potential, rather than conservation of amino acid identity, is a general feature of PfEMP1 and other divergent parasite protein families. Future structural biology studies will be required to confirm this and to identify the molecular determinants required to bind to other host receptors.

Such extensive surface variation, even across the ligand-binding surface, raises questions about whether it will be possible to design immunogens that induce immunoglobulins that can block EPCR binding by all PfEMP1. Such an immunogen would have significant value in the prevention of severe malaria. Our studies of IgG affinity purified from individuals from Tanzania provide some hope, as they reveal that natural infection has led to the acquisition of IgG with the capacity to reduce EPCR binding by diverse CIDRα1 domains and endothelial cell binding by the FCR3 IT4var20 parasite line.

How cross-inhibitory these responses can become, to what extent they protect against severe pediatric malaria, and whether improved immunogens can be developed that allow their induction remain questions for the future. In addition, it is currently unknown what fraction of EPCR-binding PfEMP1 contains domains that interact with additional endothelial receptors or whether preventing EPCR occupancy is sufficient alone to ameliorate disease symptoms. However, the retention of bonding potential across the EPCR-binding surface of the CIDRα1 domains does suggest that it might be possible to raise IgG, which present a chemical surface that mimics features of EPCR, containing the ability to bind to and block the EPCR-binding surfaces of all CIDRα1s. Future studies will need to determine whether such IgG can be generated, assessing whether the necessity for PfEMP1 to retain conserved structural features to allow EPCR binding can provide a route to target the parasite and contribute to the prevention of severe malaria.

EXPERIMENTAL PROCEDURES

More detailed methods are in the Supplemental Experimental Procedures.

Protein Expression and Purification

CIDRα1 domains for binding studies were expressed in baculovirus-infected High Five cells and purified by metal affinity chromatography. For crystallization, CIDRα1 domains were expressed in *E. coli* in inclusion bodies and were refolded on a Ni-NTA column, followed by size exclusion chromatography.

EPCR was expressed in a stable *Drosophila* S2 cell line (ExpreS^{2}ion Biotechnologies). Culture media was buffer exchanged and EPCR purified by Ni-NTA affinity chromatography and size exclusion gel chromatography. Protein for crystallography was deglycosylated by treatment with endoglycosidase H_f and endoglycosidase F3, and tags were removed using TEV protease.

Crystal Structure Determination

HB3var03 CIDRα1:EPCR and IT4var07 CIDRα1:EPCR complexes were purified by size exclusion chromatography. Crystals were grown using sitting-drop vapor diffusion. HB3var03 CIDRα1-EPCR crystals grew with a reservoir solution of 0.2 M $NaNO_3$, 0.1 M BTP (pH 8.5), 20% PEG 3350 and were cryo-cooled in well solution containing 25% ethylene glycol. IT4var07 CIDRα1-EPCR crystals grew with a reservoir solution of 0.2 M $NaNO_3$, 0.1 M BTP (pH 7.5), 20% PEG 3350 and were cryo-cooled in well solution with 25% MPD.

Data were collected on beamlines I02 and I04 (Diamond Light Source), indexed, refined using iMosflm (Leslie and Powell, 2007), and scaled using SCALA (Collaborative Computational Project, Number 4, 1994). Molecular replacement using Phaser-MR (Collaborative Computational Project, Number 4, 1994) found two copies of EPCR (PDB ID: 1L8J) in the asymmetric unit of HB3var03 CIDRα1:EPCR crystals. CIDRα1 domain models were built using a cycle of refinement, in Refmac (Collaborative Computational Project, Number 4, 1994) and autobuster (Bricogne et al., 2011), and model building was done in Coot (Emsley et al., 2010). The IT4var07 CIDRα1:EPCR structure was determined using Phaser-MR with the HB3var03 CIDRα1:EPCR complex as a search model and refined as above.

Surface Plasmon Resonance

SPR experiments were carried out in a Biacore T200 instrument (GE Healthcare). EPCR was biotinylated by incubation with BirA and coupled to a biotin capture chip (GE Healthcare) to 150 RU. Binding partners were injected for 240 s with a dissociation time of 300 s before chip regeneration. Specific responses were calculated by subtracting the response from a surface lacking EPCR. The kinetic sensorgrams were fitted to a 1:1 interaction model to allow calculation of kinetic rate constants and dissociation constant.

Isothermal Titration Calorimetry

ITC measurements were performed at 25°C in a MicroCal iTC200 System (GE Healthcare) with 60 μl of EPCR at 36 μM titrated into a cell containing 300 μl of CIDRα1 HB3var03 at 2.8 μM. Data were integrated and fit by nonlinear least-squares fitting using Origin ITC Software (GE Healthcare).

Small-Angle X-Ray Scattering

SAXS data were collected on beamline P12 at DESY (Hamburg, Germany) and processed using the ATSAS processing suite. The resulting model was converted into an envelope using Situs (Wriggers, 2010) before model docking using Sculptor (Birmanns et al., 2011).

Sequence Analysis

CIDRα1 sequences were extracted from assemblies of Illumina whole-genome sequencing data available through the MalariaGEN community and assembled with Velvet (Zerbino, 2010). The Shannon property entropy was calculated on the basis of physiochemical property groupings.

Human IgG Antibody Purification

Plasma samples collected in 2005 during a cross-sectional malaria survey in an area of high malaria transmission (Tanzania) were screened for ability to inhibit the EPCR binding of HB3var03 CIDRα1.4 or IT4var20 CIDRα1.1 in

ELISA. Inhibitory plasma were screened by ELISA for reactivity to peptides covering the EPCR binding region of HB3var03 CIDRα1.4 or IT4var20 CIDRα1.1. IgG preparations were purified by affinity to HB3var03 or IT4var20 peptides, and their binding properties were analyzed by ELISA.

Parasite Assays

Human brain microvascular endothelial cells (HBMECs) were grown to a monolayer. Ring-stage infected erythrocytes from the FCR3 IT4VAR20 parasite line were tritiated, and 24 hr later, radioactively labeled late trophozoite and schizont stages were purified and incubated with HBMECs for 1 hr at 37°C. Unbound infected erythrocytes were removed with a washing robot (Biomek 2000, Beckman Coulter) and radioactivity measured on a Topcount NXT (PerkinElmer). Adhesion was calculated as the percentage of bound radioactively labeled infected erythrocytes out of the total amount of radioactively labeled infected erythrocytes added per well.

ACCESSION NUMBERS

Coordinates and experimental data are deposited in the PDB with accession numbers 4V3D and 4V3E.

SUPPLEMENTAL INFORMATION

Supplemental Information includes Supplemental Experimental Procedures, six figures, and six tables and can be found with this article online at http://dx.doi.org/10.1016/j.chom.2014.11.007.

AUTHOR CONTRIBUTIONS

C.K.Y.L. purified and crystallized proteins, collected and analyzed SAXS data, and performed SPR, ITC, MALLS, and AUC. C.K.Y.L. and M.K.H. prepared crystals, collected data, and solved structures. E.D.L. collected data. L.T. produced protein and prepared human IgG and performed ELISA studies. J.L. contributed sera. J.S.J., B.P., and T.L. performed bioinformatic analysis. C.W.W. and J.E.V.P. performed parasite binding experiments. C.K.Y.L., L.T., T.T., T.L., and M.K.H. devised the study and wrote the manuscript.

ACKNOWLEDGMENTS

M.K.H. is funded by an Investigator Award from the Wellcome Trust. C.K.Y.L. is funded by a Medical Research Council studentship. We are grateful for the use of data from the MalariaGEN community project on *Plasmodium falciparum* population genomics as described in Miotto et al. (2013). This work was supported by the Lundbeck Foundation, Danish International Development Agency, the Danish Council for Independent Research, Medical Sciences and the Sapere Aude programme (DFF–4004-00624B), and University of Copenhagen. We thank F. Lennartz and K. Wright for comments on the manuscript and D. Staunton for assistance with biophysical characterization.

Received: August 28, 2014
Revised: September 27, 2014
Accepted: October 30, 2014
Published: December 4, 2014

REFERENCES

Avril, M., Tripathi, A.K., Brazier, A.J., Andisi, C., Janes, J.H., Soma, V.L., Sullivan, D.J., Jr., Bull, P.C., Stins, M.F., Smith, J.D., and Smith, J.D. (2012). A restricted subset of var genes mediates adherence of *Plasmodium falciparum*-infected erythrocytes to brain endothelial cells. Proc. Natl. Acad. Sci. USA *109*, E1782–E1790.

Baruch, D.I., Pasloske, B.L., Singh, H.B., Bi, X., Ma, X.C., Feldman, M., Taraschi, T.F., and Howard, R.J. (1995). Cloning the *P. falciparum* gene encoding PfEMP1, a malarial variant antigen and adherence receptor on the surface of parasitized human erythrocytes. Cell *82*, 77–87.

Batchelor, J.D., Zahm, J.A., and Tolia, N.H. (2011). Dimerization of *Plasmodium vivax* DBP is induced upon receptor binding and drives recognition of DARC. Nat. Struct. Mol. Biol. *18*, 908–914.

Batchelor, J.D., Malpede, B.M., Omattage, N.S., DeKoster, G.T., Henzler-Wildman, K.A., and Tolia, N.H. (2014). Red blood cell invasion by *Plasmodium vivax*: structural basis for DBP engagement of DARC. PLoS Pathog. *10*, e1003869.

Bertin, G.I., Lavstsen, T., Guillonneau, F., Doritchamou, J., Wang, C.W., Jespersen, J.S., Ezimegnon, S., Fievet, N., Alao, M.J., Lalya, F., et al. (2013). Expression of the domain cassette 8 Plasmodium falciparum erythrocyte membrane protein 1 is associated with cerebral malaria in Benin. PLoS ONE *8*, e68368.

Birmanns, S., Rusu, M., and Wriggers, W. (2011). Using Sculptor and Situs for simultaneous assembly of atomic components into low-resolution shapes. J. Struct. Biol. *173*, 428–435.

Bricogne, G., Blanc, E., Brandl, M., Flensburg, C., Keller, P., Paciorek, W., Roversi, P., Sharff, A., Smart, O.S., Vonrhein, C., et al. (2011). Buster version 2.10.0. (Cambridge, UK: Glob. Phasing Ltd.).

Bull, P.C., Lowe, B.S., Kortok, M., Molyneux, C.S., Newbold, C.I., and Marsh, K. (1998). Parasite antigens on the infected red cell surface are targets for naturally acquired immunity to malaria. Nat. Med. *4*, 358–360.

Capra, J.A., and Singh, M. (2007). Predicting functionally important residues from sequence conservation. Bioinformatics *23*, 1875–1882.

Carmo, M.S., Santos, M.R., Cummings, L.M., Araya, J.E., Yamauchi, L.M., Yoshida, N., Mortara, R.A., and Franco da Silveira, J. (2001). Isolation and characterisation of genomic and cDNA clones coding for a serine-, alanine-, and proline-rich protein of *Trypanosoma cruzi*. Int. J. Parasitol. *31*, 259–264.

Cham, G.K., Turner, L., Lusingu, J., Vestergaard, L., Mmbando, B.P., Kurtis, J.D., Jensen, A.T., Salanti, A., Lavstsen, T., and Theander, T.G. (2009). Sequential, ordered acquisition of antibodies to *Plasmodium falciparum* erythrocyte membrane protein 1 domains. J. Immunol. *183*, 3356–3363.

Cheng, Q., Cloonan, N., Fischer, K., Thompson, J., Waine, G., Lanzer, M., and Saul, A. (1998). stevor and rif are *Plasmodium falciparum* multicopy gene families which potentially encode variant antigens. Mol. Biochem. Parasitol. *97*, 161–176.

Claessens, A., Adams, Y., Ghumra, A., Lindergard, G., Buchan, C.C., Andisi, C., Bull, P.C., Mok, S., Gupta, A.P., Wang, C.W., et al. (2012). A subset of group A-like var genes encodes the malaria parasite ligands for binding to human brain endothelial cells. Proc. Natl. Acad. Sci. USA *109*, E1772–E1781.

Collaborative Computational Project, Number 4 (1994). The CCP4 suite: programs for protein crystallography. Acta Crystallogr. D Biol. Crystallogr. *50*, 760–763.

del Portillo, H.A., Fernandez-Becerra, C., Bowman, S., Oliver, K., Preuss, M., Sanchez, C.P., Schneider, N.K., Villalobos, J.M., Rajandream, M.A., Harris, D., et al. (2001). A superfamily of variant genes encoded in the subtelomeric region of Plasmodium vivax. Nature *410*, 839–842.

El-Sayed, N.M., Myler, P.J., Bartholomeu, D.C., Nilsson, D., Aggarwal, G., Tran, A.N., Ghedin, E., Worthey, E.A., Delcher, A.L., Blandin, G., et al. (2005). The genome sequence of Trypanosoma cruzi, etiologic agent of Chagas disease. Science *309*, 409–415.

Emsley, P., Lohkamp, B., Scott, W.G., and Cowtan, K. (2010). Features and development of Coot. Acta Crystallogr. D Biol. Crystallogr. *66*, 486–501.

Gardner, M.J., Hall, N., Fung, E., White, O., Berriman, M., Hyman, R.W., Carlton, J.M., Pain, A., Nelson, K.E., Bowman, S., et al. (2002). Genome sequence of the human malaria parasite *Plasmodium falciparum*. Nature *419*, 498–511.

Gonçalves, B.P., Huang, C.Y., Morrison, R., Holte, S., Kabyemela, E., Prevots, D.R., Fried, M., and Duffy, P.E. (2014). Parasite burden and severity of malaria in Tanzanian children. N. Engl. J. Med. *370*, 1799–1808.

Gupta, S., Snow, R.W., Donnelly, C.A., Marsh, K., and Newbold, C. (1999). Immunity to non-cerebral severe malaria is acquired after one or two infections. Nat. Med. *5*, 340–343.

Higgins, M.K. (2008). The structure of a chondroitin sulfate-binding domain important in placental malaria. J. Biol. Chem. *283*, 21842–21846.

Higgins, M.K., and Carrington, M. (2014). Sequence variation and structural conservation allows development of novel function and immune evasion in parasite surface protein families. Protein Sci. *23*, 354–365.

Hodder, A.N., Czabotar, P.E., Uboldi, A.D., Clarke, O.B., Lin, C.S., Healer, J., Smith, B.J., and Cowman, A.F. (2012). Insights into Duffy binding-like domains through the crystal structure and function of the merozoite surface protein MSPDBL2 from *Plasmodium falciparum*. J. Biol. Chem. *287*, 32922–32939.

Howell, D.P., Levin, E.A., Springer, A.L., Kraemer, S.M., Phippard, D.J., Schief, W.R., and Smith, J.D. (2008). Mapping a common interaction site used by *Plasmodium falciparum* Duffy binding-like domains to bind diverse host receptors. Mol. Microbiol. *67*, 78–87.

Kasper, L.H., Crabb, J.H., and Pfefferkorn, E.R. (1983). Purification of a major membrane protein of *Toxoplasma gondii* by immunoabsorption with a monoclonal antibody. J. Immunol. *130*, 2407–2412.

Khunrae, P., Dahlbäck, M., Nielsen, M.A., Andersen, G., Ditlev, S.B., Resende, M., Pinto, V.V., Theander, T.G., Higgins, M.K., and Salanti, A. (2010). Full-length recombinant *Plasmodium falciparum* VAR2CSA binds specifically to CSPG and induces potent parasite adhesion-blocking antibodies. J. Mol. Biol. *397*, 826–834.

Klein, M.M., Gittis, A.G., Su, H.P., Makobongo, M.O., Moore, J.M., Singh, S., Miller, L.H., and Garboczi, D.N. (2008). The cysteine-rich interdomain region from the highly variable *plasmodium falciparum* erythrocyte membrane protein-1 exhibits a conserved structure. PLoS Pathog. *4*, e1000147.

Kraemer, S.M., and Smith, J.D. (2006). A family affair: var genes, PfEMP1 binding, and malaria disease. Curr. Opin. Microbiol. *9*, 374–380.

Kyes, S.A., Rowe, J.A., Kriek, N., and Newbold, C.I. (1999). Rifins: a second family of clonally variant proteins expressed on the surface of red cells infected with *Plasmodium falciparum*. Proc. Natl. Acad. Sci. USA *96*, 9333–9338.

Lavstsen, T., Salanti, A., Jensen, A.T., Arnot, D.E., and Theander, T.G. (2003). Sub-grouping of Plasmodium falciparum 3D7 var genes based on sequence analysis of coding and non-coding regions. Malar. J. *2*, 27.

Lavstsen, T., Turner, L., Saguti, F., Magistrado, P., Rask, T.S., Jespersen, J.S., Wang, C.W., Berger, S.S., Baraka, V., Marquard, A.M., et al. (2012). *Plasmodium falciparum* erythrocyte membrane protein 1 domain cassettes 8 and 13 are associated with severe malaria in children. Proc. Natl. Acad. Sci. USA *109*, E1791–E1800.

Leech, J.H., Barnwell, J.W., Miller, L.H., and Howard, R.J. (1984). Identification of a strain-specific malarial antigen exposed on the surface of *Plasmodium falciparum*-infected erythrocytes. J. Exp. Med. *159*, 1567–1575.

Leslie, A.G.W., and Powell, H.R. (2007). Processing diffraction data with mosflm. Evol. Methods Macromol. Crystallogr. *41*.

Lin, D.H., Malpede, B.M., Batchelor, J.D., and Tolia, N.H. (2012). Crystal and solution structures of *Plasmodium falciparum* erythrocyte-binding antigen 140 reveal determinants of receptor specificity during erythrocyte invasion. J. Biol. Chem. *287*, 36830–36836.

Lusingu, J.P., Jensen, A.T., Vestergaard, L.S., Minja, D.T., Dalgaard, M.B., Gesase, S., Mmbando, B.P., Kitua, A.Y., Lemnge, M.M., Cavanagh, D., et al. (2006). Levels of plasma immunoglobulin G with specificity against the cysteine-rich interdomain regions of a semiconserved *Plasmodium falciparum* erythrocyte membrane protein 1, VAR4, predict protection against malarial anemia and febrile episodes. Infect. Immun. *74*, 2867–2875.

Malpede, B.M., Lin, D.H., and Tolia, N.H. (2013). Molecular basis for sialic acid-dependent receptor recognition by the *Plasmodium falciparum* invasion protein erythrocyte-binding antigen-140/BAEBL. J. Biol. Chem. *288*, 12406–12415.

Manske, M., Miotto, O., Campino, S., Auburn, S., Almagro-Garcia, J., Maslen, G., O'Brien, J., Djimde, A., Doumbo, O., Zongo, I., et al. (2012). Analysis of *Plasmodium falciparum* diversity in natural infections by deep sequencing. Nature *487*, 375–379.

Miller, L.H., Baruch, D.I., Marsh, K., and Doumbo, O.K. (2002). The pathogenic basis of malaria. Nature *415*, 673–679.

Miotto, O., Almagro-Garcia, J., Manske, M., Macinnis, B., Campino, S., Rockett, K.A., Amaratunga, C., Lim, P., Suon, S., Sreng, S., et al. (2013). Multiple populations of artemisinin-resistant Plasmodium falciparum in Cambodia. Nat. Genet. *45*, 648–655.

Mirny, L.A., and Shakhnovich, E.I. (1999). Universally conserved positions in protein folds: reading evolutionary signals about stability, folding kinetics and function. J. Mol. Biol. *291*, 177–196.

Moxon, C.A., Wassmer, S.C., Milner, D.A., Jr., Chisala, N.V., Taylor, T.E., Seydel, K.B., Molyneux, M.E., Faragher, B., Esmon, C.T., Downey, C., et al. (2013). Loss of endothelial protein C receptors links coagulation and inflammation to parasite sequestration in cerebral malaria in African children. Blood *122*, 842–851.

Naka, I., Patarapotikul, J., Hananantachai, H., Imai, H., and Ohashi, J. (2014). Association of the endothelial protein C receptor (PROCR) rs867186-G allele with protection from severe malaria. Malar. J. *13*, 105.

Nielsen, M.A., Staalsoe, T., Kurtzhals, J.A., Goka, B.Q., Dodoo, D., Alifrangis, M., Theander, T.G., Akanmori, B.D., and Hviid, L. (2002). *Plasmodium falciparum* variant surface antigen expression varies between isolates causing severe and nonsevere malaria and is modified by acquired immunity. J. Immunol. *168*, 3444–3450.

Oganesyan, V., Oganesyan, N., Terzyan, S., Qu, D., Dauter, Z., Esmon, N.L., and Esmon, C.T. (2002). The crystal structure of the endothelial protein C receptor and a bound phospholipid. J. Biol. Chem. *277*, 24851–24854.

Rask, T.S., Hansen, D.A., Theander, T.G., Gorm Pedersen, A., and Lavstsen, T. (2010). *Plasmodium falciparum* erythrocyte membrane protein 1 diversity in seven genomes—divide and conquer. PLoS Comput. Biol. *6*, e1000933.

Robinson, B.A., Welch, T.L., and Smith, J.D. (2003). Widespread functional specialization of *Plasmodium falciparum* erythrocyte membrane protein 1 family members to bind CD36 analysed across a parasite genome. Mol. Microbiol. *47*, 1265–1278.

Salanti, A., Dahlbäck, M., Turner, L., Nielsen, M.A., Barfod, L., Magistrado, P., Jensen, A.T., Lavstsen, T., Ofori, M.F., Marsh, K., et al. (2004). Evidence for the involvement of VAR2CSA in pregnancy-associated malaria. J. Exp. Med. *200*, 1197–1203.

Sander, A.F., Lavstsen, T., Rask, T.S., Lisby, M., Salanti, A., Fordyce, S.L., Jespersen, J.S., Carter, R., Deitsch, K.W., Theander, T.G., et al. (2014). DNA secondary structures are associated with recombination in major *Plasmodium falciparum* variable surface antigen gene families. Nucleic Acids Res. *42*, 2270–2281.

Schwede, A., and Carrington, M. (2010). Bloodstream form Trypanosome plasma membrane proteins: antigenic variation and invariant antigens. Parasitology *137*, 2029–2039.

Singh, S.K., Hora, R., Belrhali, H., Chitnis, C.E., and Sharma, A. (2006). Structural basis for Duffy recognition by the malaria parasite Duffy-binding-like domain. Nature *439*, 741–744.

Smith, J.D., Chitnis, C.E., Craig, A.G., Roberts, D.J., Hudson-Taylor, D.E., Peterson, D.S., Pinches, R., Newbold, C.I., and Miller, L.H. (1995). Switches in expression of *Plasmodium falciparum* var genes correlate with changes in antigenic and cytoadherent phenotypes of infected erythrocytes. Cell *82*, 101–110.

Smith, J.D., Subramanian, G., Gamain, B., Baruch, D.I., and Miller, L.H. (2000). Classification of adhesive domains in the *Plasmodium falciparum* erythrocyte membrane protein 1 family. Mol. Biochem. Parasitol. *110*, 293–310.

Smith, J.D., Rowe, J.A., Higgins, M.K., and Lavstsen, T. (2013). Malaria's deadly grip: cytoadhesion of *Plasmodium falciparum*-infected erythrocytes. Cell. Microbiol. *15*, 1976–1983.

Su, X.Z., Heatwole, V.M., Wertheimer, S.P., Guinet, F., Herrfeldt, J.A., Peterson, D.S., Ravetch, J.A., and Wellems, T.E. (1995). The large diverse gene family var encodes proteins involved in cytoadherence

and antigenic variation of *Plasmodium falciparum*-infected erythrocytes. Cell *82*, 89–100.

Tolia, N.H., Enemark, E.J., Sim, B.K., and Joshua-Tor, L. (2005). Structural basis for the EBA-175 erythrocyte invasion pathway of the malaria parasite *Plasmodium falciparum*. Cell *122*, 183–193.

Turner, L., Lavstsen, T., Berger, S.S., Wang, C.W., Petersen, J.E., Avril, M., Brazier, A.J., Freeth, J., Jespersen, J.S., Nielsen, M.A., et al. (2013). Severe malaria is associated with parasite binding to endothelial protein C receptor. Nature *498*, 502–505.

Vigan-Womas, I., Guillotte, M., Juillerat, A., Hessel, A., Raynal, B., England, P., Cohen, J.H., Bertrand, O., Peyrard, T., Bentley, G.A., et al. (2012). Structural basis for the ABO blood-group dependence of *Plasmodium falciparum* rosetting. PLoS Pathog. *8*, e1002781.

Wriggers, W. (2010). Using Situs for the integration of multi-resolution structures. Biophys Rev *2*, 21–27.

Zerbino, D.R. (2010). Using the Velvet de novo assembler for short-read sequencing technologies. Curr. Protoc. Bioinformatics *Chapter 11*, 5.

Cell Host & Microbe, Volume *17*

Supplemental Information

Structural Conservation Despite Huge Sequence Diversity Allows EPCR Binding by the PfEMP1 Family Implicated in Severe Childhood Malaria

Clinton K.Y. Lau, Louise Turner, Jakob S. Jespersen, Edward D. Lowe, Bent Petersen, Christian W. Wang, Jens E.V. Petersen, John Lusingu, Thor G. Theander, Thomas Lavstsen, and Matthew K. Higgins

Supplemental experimental procedures

Expression and purification of CIDRα1 domains

Domain sequences originate from *Plasmodium falciparum* reference genomes (Rask et al., 2010) or sequenced Tanzanian patient isolates (four digit names from Lavstsen et al., 2012 and unpublished; ERSxxxxxx names from Miotto et al., 2013). In addition to previously published CIDRα1 domains (Turner et al., 2013), new CIDRα1 DNA sequences were optimised for expression in *Trichoplusia ni* cells using the software GeneOptimizer. They were synthesised by Geneart (Regensburg, Germany) after modification to include C-terminal V5 and His tags and were subcloned into the baculovirus expression vector pAcGP67-A (BD Biosciences). FlashBac DNA (Oxford Expression Technologies, OE-100150) was co-transfected with the recombinant vectors into Sf9 insect cells for the generation of recombinant virus particles.

High-Five insect cells grown in 600 ml of serum-free media (10486; GIBCO) were infected with 18 ml of the second amplification of the recombinant virus particles. Two days later the cells were centrifuged (8000g, 4 °C, 10 min), and the supernatant was filtered using two 10-kDa NMWC PES membranes (0.45 μm) (56-4112-04; GE Healthcare) with a total surface area of 200 cm^2. The supernatant was then concentrated to 30 ml and diafiltrated using an ÄKTA cross-flow (GE Healthcare) into buffer A (20 mM Tris (pH 9.0) and 500 mM NaCl). Retentate was recovered from the system and filtered (0.2 μm), yielding a final volume of 40 ml. Before loading onto a 1 ml HisSelect column (H8286; Sigma-Aldrich), 150 μl of 1 M imidazole (pH 7.4; Sigma-Aldrich) was added to the sample, giving a final imidazole concentration of 15 μM. The bound protein was eluted with buffer A+200 mM imidazole.

For crystallisation, CIDRα1 domains from HB3var03 and IT4var07 were expressed in *E. coli*. Domain boundaries were selected using a structure-based alignment against pdb 2YK0 (Vigan-Womas et al., 2012) using FUGUE (Shi et al., 2001). Genes were amplified from HB3 or IT4 strain *P. falciparum* genomic DNA respectively and were cloned into the pEt15b vector. This allowed expression of the protein with an N-terminal hexa-histidine tag and a TEV cleavage site using *Escherichia coli* (BL-21 strain). Transformed *E. coli* were grown up to an optical density at 600 nm of 1.0 and expression was induced by the addition of IPTG to a final concentration of 1 mM. Cells were harvested after 3 h at 27°C. The CIDRα1 domains expressed in the form of inclusion bodies, which were unfolded by incubation at room temperature in 6 M guanidine-hydrochloride, 20 mM Tris pH 8, 300 mM NaCl, 15 mM imidazole for 15 h. Refolding was achieved by gradual buffer exchange into 20 mM Tris pH 8, 300 mM NaCl, 15 mM imidazole in the presence of a glutathione redox buffer (3 mM reduced glutathione, 0.3 mM oxidised glutathione) while the protein was bound to a Ni-NTA column. Refolded protein was eluted and further purified by size exclusion gel chromatography (HiLoad Superdex 75 16/60, GE Healthcare) into 20 mM HEPES pH 7.5, 150 mM NaCl.

Single site mutants were generated from the wild type HB3var03 CIDRα1 construct as described by the Quikchange mutagenesis method (Stratagene) and

plasmid sequences were verified. Proteins from these constructs were expressed and purified in *E. coli* as above.

Recombinant EPCR expression and purification

A codon-optimised synthetic gene was designed for EPCR (Syngene), using domain boundaries obtained from the crystal structure of pdb 1L8J (Oganesyan et al., 2002) and was produced by Geneart. This gene was cloned into the pExpreS^2 vector, in frame with a BIP leader sequence and an N-terminal extension containing a BAP tag, an N-terminal hexa-histidine tag, and a TEV cleavage site. The vector was then transfected into *Drosophila* S2 cells using ExpreS^2 Insect TRx5 liposome transfection reagent (Expres^{2}ion Biotechnologies). Stably transfected cells were selected using zeocin (Invitrogen, Thermo Fisher Scientific). Culture media containing EPCR was buffer exchanged into 20 mM Tris pH 8, 500 mM NaCl and protein was purified by Ni-NTA affinity chromatography and size exclusion gel chromatography (HiLoad Superdex 75 16/60, GE Healthcare) using 20 mM HEPES pH 7.5, 150 mM NaCl.

Protein for crystallography was deglycosylated by treatment with endoglycosidase H_f (Sigma) and endoglycosidase F3 at enzyme:protein ratios of 1:50 in 50 mM MES pH 6.5 for 15 h. N-terminal tags were cleaved using TEV protease at an enzyme:protein ratio of 1:50 in PBS (Melford) with 3 mM reduced glutathione, 0.3 mM oxidised glutathione for 15 h at 25°C.

CIDRα1-EPCR complex crystallisation

HB3var03 and IT4var07 CIDRα1s were mixed separately with EPCR at 1:1 molar ratios and complexes were obtained by size exclusion chromatography on a Superdex 75 HiLoad 16/60 column (GE Healthcare). Fractions containing complexes were concentrated up to 10.7 mg/ml (HB3var03) and 8.1 mg/ml (IT4var07). Crystals were grown using the sitting-drop vapour-diffusion method in 96 well plates. Initial crystals were obtained through a broad screen with droplets containing 100 nl of protein solution mixed with 100 nl of well solution. Optimisation screens were performed including seed stocks obtained from initial crystals, with each droplet contained 100 nl of protein solution, 50 nl of seed solution and 50 nl of well solution and 50 nl of additive solution (Silver Bullets, Hampton Research).

HB3var03 CIDRα1-EPCR crystals grew as needle clusters with a reservoir solution of 0.2 M $NaNO_3$, 0.1 M BTP pH 8.5, 20% PEG 3350. Needle clusters were transferred into a drop of well solution containing 25% ethylene glycol and single needles detached. These needles were then cryo-cooled in liquid nitrogen for storage and data collection.

IT4var07 CIDRα1-EPCR crystals grew as hexagonal prisms with a reservoir solution of 0.2 M $NaNO_3$, 0.1 M BTP pH 7.5, 20% PEG 3350. MPD was gradually

added to the crystals in a drop of mother liquor to a final concentration of 25%. Crystals were cryo-cooled in liquid nitrogen for storage and data collection.

Data collection and structure determination

Data from crystals containing the HB3var03 CIDRα1-EPCR complex were collected at beamline I04 (Diamond Light Source, UK) using radiation with a wavelength of 0.98Å and a Pilatus 6M-F detector. These data were indexed and refined using iMosflm (Leslie and Powell, 2007), then scaled using SCALA (CCPN4, 1994) to resolution of 2.65 Å. Molecular replacement using Phaser-MR (CCPN4, 1994) found two copies of EPCR (pdb code 1L8J) (Oganesyan et al., 2002) in the asymmetric unit. The models of the CIDRα1 domains were built into the remaining electron density using an iterative cycle of refinement using Refmac (CCPN4, 1994) and autobuster (Bricogne et al., 2011) and manual model building using Coot (Emsley et al., 2010).

Data from crystals containing the IT4var07 CIDRα1-EPCR complex were collected at beamline I02 (Diamond Light Source, UK) using a Pilatus 6M detector. These data were indexed and refined using iMosflm and scaled using SCALA to a resolution of 2.9 Å. Phaser-MR using the structure of the HB3var03 CIDRα1-EPCR complex found one copy in the asymmetric unit. The model of the IT4var07 CIDRα1:EPCR complex was built using iterative cycles of manual model building using Coot and refinement using autobuster.

Surface plasmon resonance

EPCR was coupled to an SPR chip using a biotin attached to the N-terminal BAP tag. This strategy was designed to allow EPCR to be immobilised with an orientation matching that found on the endothelial surface, and to generate a surface that could readily be regenerated. The S2 cell produced EPCR was not biotinylated during expression, so 1 mg EPCR (30 μM) in 20 mM HEPES pH 7.5, 150 mM NaCl was incubated with 20 μg BirA, 0.3 μM biotin, 5 mM ATP, for 15 h at 25°C.

SPR experiments were carried out on a Biacore T200 instrument (GE Healthcare). All experiments were performed in 20 mM HEPES pH 7.5, 150 mM NaCl, 0.005% Tween-20 at 25°C. Two-fold dilution series of each CIDRα1 were prepared for injection over an EPCR-coated chip. For each cycle, biotinylated recombinant EPCR was immobilised on a CAP chip using the Biotin Capture Kit (GE Healthcare) to a total loading of 150 RU. Binding partners were injected for 240 s with a dissociation time of 300 s. The chip was regenerated in between cycles using regeneration solution from the Biotin Capture Kit (GE Healthcare). The specific binding response of the CIDRα1s to EPCR was determined by subtracting the response given by CIDRα1 from a surface to which no EPCR had been coupled. The kinetic sensorgrams were globally fitted to a 1:1 interaction model to allow calculation of the association rate constant, k_a; the dissociation

rate constant, k_d; and the dissociation constant K_d using BIAevaluation software version 1.0 (GE Healthcare).

Isothermal titration calorimetry

ITC measurements were carried out on a MicroCal iTC200 System (GE Healthcare). Samples were dialysed for 15 h into 20 mM HEPES pH 7.5, 150 mM NaCl at 4°C. Experiments were performed at 25°C with 60 µl of EPCR at 50 µM titrated into a cell containing 300 µl of HB3var03 CIDRα1.4 at 5 µM. Concentrations of both components were checked using a BCA assay kit (Merck Millipore). Data were integrated and fit by nonlinear least-squares fitting using Origin ITC Software (GE Healthcare).

Small angle X-ray scattering

SAXS data were collected on beamline P12 at DESY (Hamburg, Germany) at a wavelength of 0.124 nm using a Pilatus 2M pixel X-ray detector. For each protein sample, a series of data sets were collected for a 2-fold dilution series from 4 mg/ml to 0.125 mg/ml, with buffer measurements taken in between each sample measurement. All samples were in 20 mM HEPES pH 7.5, 150 mM NaCl.

Concentration series for each sample, after normalisation and buffer subtraction, were extrapolated to zero to check for concentration dependent aggregation in Primus (Konarev et al., 2003). The radius of gyration, R_g, and zero angle scattering, I(0), were calculated using AutoRg. Data points from the lowest scattering angle (q), as well as at those at higher q with a low signal to noise ratio, were removed, depending on AutoRg results and Kratky plots. Inverse Fourier calculations of I(q) were used to yield P(r) functions, I(0), R_g and the maximum dimension (D_{max}) using GNOM (Svergun, 1992).

Initial bead models for these data were created *ab initio* using DAMMIF (Franke and Svergun, 2009). These were averaged using DAMAVER (Volkov and Svergun, 2003), and refined from the averaged model, based on the original P(r) function, using DAMMIN (Svergun, 1999). The resulting model was converted into an envelope using Situs (Wriggers, 2010), before docking pdb models into the envelope using Sculptor (Birmanns et al., 2011). Chain A from the HB3var03:EPCR structure was used for the CIDRα1 domain model and chain B from the HB3var03:EPCR structure as the EPCR model in the HB3var03:EPCR envelope; the NTSDBL domain from PDB 2YK0 (Vigan-Womas et al., 2012) and the DBL3x domain from PDB 3BQK (Higgins, 2008) were used in addition to the aforementioned domains as models in the DD2var32:EPCR envelope.

Size exclusion chromatography-multiangle laser light scattering

Samples were purified by size exclusion chromatography on a Superdex 75 gel filtration column (analytical grade, GE Healthcare), then analysed using laser light scattering detected at 662 nm wavelength at 8 scattering angles between 20.6° and 149.1° using a Heleos 8 instrument (Wyatt Technology). Molecular weights were calculated using the Zimm equation by ASTRA 6.1 (Wyatt Technology).

Equilibrium analytical ultracentrifugation

Analytical ultracentrifugation was carried out using a Beckman Optima XL-1 analytical ultracentrifuge. Experiments were run until equilibrium at 20°C, with HB3var03 at 8 μM. Absorbance data were taken at a wavelength of 280 nm at equilibrium at 10,000, 12,000, 14,000 and 16,000 rpm analysed the data using sedphat (Vistica et al., 2004), globally calculating the molecular weight modeling a single species of an interacting system.

Sequence analysis

To facilitate analysis of CIDRα1 sequence diversity we expanded our collection of CIDRα1 domains from the previously described 66 sequences, originating mainly from seven whole genome sequenced parasites (Lavstsen et al., 2012; Rask et al., 2010), with domains extracted from assemblies of Illumina whole genome sequencing data from 226 samples collected in both Africa and Asia (Miotto et al., 2013) (Study number ERP000190), available through the MalariaGEN community. All data available from each sample was collectively assembled with Velvet (Zerbino, 2010) using an optimized k-mer value. Assembled contigs were subjected to the Virtual Ribosome open reading frame (ORF) finder (Wernersson, 2006) searching all reading frames, resulting in ~3.4 million ORFs ranging from 21 bp to 30,940 bp, median 258 bp (starting with any codon) and ~850,000 ORFs longer than 750 bp. The 66 different previously classified CIDRα1 sequences (Lavstsen et al., 2012; Rask et al., 2010) were each used to blastp search (Altschul, 1997) the ORFs keeping all hits with an expectation value of $< 10^{-10}$ to extract even very divergent CIDRα1 variants. This resulted in ~1050 hits for each query and a total of 1,894 non-redundant ORF hits. The amino acid sequence of these ORFs were then aligned together with the 66 query sequences using MUSCLE (Edgar, 2004) and the CIDRα1 region excised. Incomplete domain sequences were discarded (domain borders defined in Turner et al, 2013), and the remaining sequences were blastp searched against a library of all known annotated PfEMP1 domains (Rask et al., 2010) (including 649 CIDR domains of all types). Query domains whose best hit was not a CIDRα1 domain were discarded, leaving 831 complete CIDRa1 sequences originating from 198 of the 226 assembled samples. Hand corrected MUSCLE alignments were used to generate sequence logos by WebLogo 3 (Crooks et al., 2004) and

sequence distance trees by MEGA (Tamura et al., 2013). As a measure of the residue-wise conservation/variation of EPCR binding CIDRα1 domains, the Shannon property entropy was calculated on the basis of the physiochemical property groupings defined by Mirny and Shakhnovich (aliphatic [AVLIMC], aromatic [FWYH], polar [STNQ], positive [KR], negative [DE] and special conformation [PG]) and using sequence weighting (Capra and Singh, 2007; Mirny and Shakhnovich, 1999). As shown in Figure 1 and Table S1 these domains grouped into the eight previously defined subtypes with the additional separation of CIDRα1.5, -6 and -8 variants into two, i.e. CIDRα1.5a/b, 1.6a/b and 1.8a/b.

Human IgG antibody purification

Plasma samples collected in 2005 during a cross sectional malaria survey in an area of high malaria transmission (Tanzania) were screened for the ability to inhibit the EPCR binding of HB3var03 CIDRα1.4 or IT4var20 CIDRα1.1 in ELISA. A set of 45 or 76 plasma samples were screened for each protein, respectively. 41 of 45 and 51 of 76 samples inhibited the EPCR binding to OD values <50% of the no plasma control. Inhibitory plasma samples were then screened by ELISA for reactivity to peptides covering the EPCR binding region of HB3var03 CIDRα1.4 or IT4var20 CIDRα1.1, respectively.

The HB3var03 peptide sequence:
LFTNKHDIPKKYYLNINDLFDSFFFQVIYKFNEGEAKWNELKENLKKQIASSKANNGT
KDSEA.
The IT4var20 peptide sequence:
VFGNNNRMSYIYYNNLSRVFDSFLFQVMFALDQDEKGKWDQFTEDLKKKFEPSKTNT
PTGKSQD

Fifteen of 41 (average donor age: 10.5 years; range 4.3-14.3 years) and 17 of 51 (average donor age: 9.4 years; range 4.7-15.3 years) samples were found to react with the respective peptide (OD>0.7). Plasma interacting with each peptide was collected to create two pools and IgG was purified yielding approximately 30 mg total IgG/pool. These IgG preparations were then purified by affinity to the HB3var03 or the IT4var20 peptides to generate a human anti-HB3var03 peptide IgG preparation (pool A) and a human anti-IT4var20 peptide IgG preparation (pool B). The IgG yield was 0.2 mg for both preparations.

IgG was prepared from each pool according to manufacturer's protocol (GammaBind Plus Sepharose, GE Healthcare). Briefly, the plasma was diluted two fold in PBS, filtered and passed seven times through 1 ml packed slurry. After washing, the bound IgG was eluted in a total of 10 ml using Tris–glycine (pH 2.4), and neutralised by dialysis against phosphate-buffered saline.

The affinity purification was carried out according to the manufacturer's protocol. Briefly, 1 mg of each peptide was coupled to NHS columns (NHS activated HP, GE Healthcare). The dialysed IgG from each pool was passed through the respective column twice. Both the eluted sample and the run-through IgG were dialysed against phosphate-buffered saline and the affinity

purified IgG sample was concentrated up using a spin column (Vivaspin VS2022) to a concentration of 0.2 mg/ml.

ELISA

Unless stated otherwise, ELISAs were carried out as described here. Recombinant human EPCR and CD36 were coated at 3 µg/ml and PfEMP1 proteins/peptides and APC were coated at 5 µg/ml in PBS, overnight at 4 °C. Plates were blocked using PBS+3% skimmed milk (150µl/well) and washed three times after all steps with PBS+0.05% Tween-20 (all 50µl/well). All test samples and secondary antibodies were diluted in PBS+1% skimmed milk. All tests were run in duplicate and incubations were carried out at room temperature, for 1 hour with gentle shaking. Plates were developed using 100 µl of a phosphate solution with 0.012% H_2O_2 substrate and o-phenylenediamine per well. The colorimetric reaction was stopped with 100 µl of 3 M H_2SO_4 after 10 minutes and the optical density (OD) was measured at 490 nm. The quality of the APC was tested by confirming binding to EPCR.

For specific cases:

PfEMP1:EPCR binding: Recombinant CIDRα1 domains were added to EPCR coated plates at a concentration of 5 ug/ml. Secondary antibody anti-V5-HRP was diluted at 1:3000 (Invitrogen R96125).

Plasma sample screens for inhibition of CIDRα1:EPCR binding: Recombinant HB3var03 CIDRα1.4 or IT4var20 CIDRα1.1 protein was pre-incubated at 0.5 ug/ml with 25% plasma, as well as a no plasma control. The protein-plasma sample was then transferred to EPCR coated plates and developed using anti-V5-HRP at 1:3000.

Plasma sample screens for recognition of peptides: Plates coated with peptides as described were incubated with a 1:50 dilution of plasma in PBS+1% skimmed milk and developed using rabbit anti-human IgG-HRP (Dako P214) at 1:3000.
Testing CIDRα1 reactivity of affinity purified IgG: IgG pools were added to recombinant CIDRα coated plates at 1:50 in PBS+1% milk. Secondary rabbit anti-human IgG-HRP was added at 1:3000 for detection.

Testing CIDRα1 reactivity of affinity purified IgG: IgG pools were added to recombinant CIDRα or APC coated plates at 1:50 in PBS+1% milk. Secondary rabbit anti-human IgG-HRP was added at 1:3000 for detection.

Testing CIDRα1:EPCR binding inhibition of affinity purified IgG: Recombinant CIDRα1 at 0.0012 mg/ml was pre-incubated with nothing or with human IgG preparations at concentrations shown in Figure 6 and Figure S6 for 1 hour before transferring to plates coated with EPCR or CD36. Secondary anti-V5-HRP was added at 1:3000.

Parasite assays

The FCR3 IT4VAR20 parasite line was cultured and PfEMP1 expression was maintained by selection for binding to recombinant EPCR as described (Turner et al., 2013). The parasite adhesion assay was carried out as previously described (Turner et al., 2013). In short, HBMECs kindly provided by M. Stins (Johns Hopkins University) were grown to a monolayer over two days. Ring-stage infected erythrocytes were radioactively labelled with tritiated hypoxanthine the day before the adhesion assay. Radioactively labelled late trophozoite and schizont stages were purified and adjusted to 1.25×10^7 cells ml^{-1} in 2% FCS (in RPMI 1640). For binding inhibition, antibodies or proteins diluted in PBS were added to HBMECs in triplicates to final concentrations as shown in figure 3. PBS alone was added as a control**.** Twenty microlitres of late-stage infected erythrocytes were added to HBMECs and co-incubated for 1 h at 37 °C. Unbound infected erythrocytes were removed with a washing robot (Biomek 2000, Beckman Coulter) and radioactivity was measured on a Topcount NXT (PerkinElmer). The total amount of radioactivity added per well (max-value) was measured and adhesion was calculated as the proportion (percentage) of bound radioactively labelled infected erythrocytes out of the total amount of radioactively labelled infected erythrocytes added per well. The binding was then normalised into units by assigning the value 100 to the percentage radioactivity bound and recovered under optimal conditions.

Sequences of recombinant CIDR domains used in this study

```
>CIDRa1.4 HB3var03_HB3
PDCGVECKNETCTPKTVIYPDCGKNEKYEPPGDAKNTEINVINSGDKEGYIFEKLSEFC
TNENNENGKNYEQWKCYYDNKKNNNKCKMEINIANSKLKNKITSFDEFFDFWVRKL
LIDTIKWETELTYCINNTDVTDCNKCNKNCVCFDKWVKQKEDEWTNIMKLFTNKHD
IPKKYYLNINDLFDSFFFQVIYKFNEGEAKWNELKENLKKQIASSKANNGTKDSEAAIK
VLFNHIKEIATICKDNNTNEGC
>CIDRa1.1 IT4var20_IT4
PDCVVQCKGGKCTEDKKNDKCRSKIIKKILQSEEPTEIHVLNSDDKQGDITKKLEVFCS
STTNYEGRNVQKWKCYNKNSDYNNCEMNISSYKDSTDANVMLSVECFHSWAKNLLI
DTIKWEHQLKNCINNTNVTYCESKCIKNCECYEKWIKRKEHEWEKVKNVFGNNNRM
SYIYYNNLSRVFDSFLFQVMFALDQDEKGKWDQFTEDLKKKFEPSKTNTPTGKSQDAI
EFLLDHLKDNALTCRDNNSNESCDVSKKVKTNPC
>CIDRa1.1 PFD0020c_3D7
PDCVVECDGKTCTQKTDDDKNCRSKIIQKILESETPIEIEVLYSDDKQGVITEKLKDFCR
GPNNYNDENLQKWKCYNKNSEYNKCEMISWLYQDPKEYNLMLSVECFHSWAKNLLI
DTIRWEHQLKNCINNTNVTDCTSKCIKNCECYEAWIERKKDEWEKLKEVLNKKDETS
HNYYNKLKDVFDRFLFQVMFALDQDEKGKWDQFTEDLKKKFGPSVESAGTANSQDA
IEFLLDHLKDNALTCRDNNSIKPCTYPPNPTPNPC
>CIDRa1.8a MAL6P1.316_3D7
PACVVECDGGKCEEKKNSDGTCIEAQIYTVVRDETPTPIKVLFSGDHQKDITKKLSSFC
KNPESENNRDYQTWQCYYKSSDYNNCEMKGSLYKVEGDPNIIVSHECFHLWVQSLLI
```

DTIKWETKLKKCINNTNVTNCYNKCNKNCECFENWVEQKKKEWENVNDVYKDQKQ
SLGIYYEKLENLFKSNFFQVMKALEGDEKGKWYQFKDDLKKKFEPSEKNTRTTDSQD
AIKLILDHLKDNATTCKDNNSLEEDENC
>CIDRa1.6a PFD1235w_3D7
PECGVQCSGTTCTPKKVIHPNCKDKETYEPGDAKTTDITVLYSGDEEGDIAQKLQDFC
NDKNKENDENYEKWQCYYKSSEINKCQMTPSSHKVPKHGYIMSFYAFFDLWVKNLLI
DSINWKNDLTNCINNTNVTDCKNDCNTNCKCFENWAKTKENEWKKVKTIYKNENG
NTNNYYKKLNNHFQGYFFHVMKELNKEEKWYKLMEDLKEKIDSSNLKNGTKDSEGA
IKVLFDHLKDIAERCIDNNSKDSC
>CIDRa1.6b IT4var18_IT4
PYCGLDCGGKTCTAKQEIYPDCVYNGAYEPPNGAETTEITVLYNDNEGDMSKKLSQFC
SNENKENIENYQKWKCYYKDRDDIECEMISSSQKDEKHRKVMIFYNFFDLWVKNLLR
DSIKWEIELKDCINNTNVTDCNNKCNKNCECFDEWVKQKEKEWGSIKDVLKKESNM
PEKYYININKNFEFYFFRVMFELKKEEKWNKLMENLRIKIKSSKSNKGTIDSDGAIKVL
FDHLKEIAEKCIDNNSKDSC
>CIDRa1.6a HB3var02_HB3
PECGVQCSGTTCTPKKVIHPNCKDKETYEPGDAKTTDITVLYSGDEEGDIAQKLQDFC
NDKNKENDENYEKWQCYYKSSEINKCQMTRSSKKVPKHRGVMSFYAFFDLWVKNLL
IDSINWKNDLTNCINNTNVTDCNKDCNKNCVCFDQWVIKKEEEWKNVNKVFENKNI
DLHDYYNKLNKHFEGYFFHVMKELNKEEKWYKLMEDLKEKIDFSKGKADGKNSEGA
IKVLFDHLKEIAEKCIHNNSNESCEASTNRTPNPC
>CIDRa1.1 IT4var06_IT4
PDCVVQCDGKRCAEDTKDNNCRSKIIEEILQSEETTDIYVLYSGKGPGAITKKLHDFCS
NTNKEDRKYYKKWKCYNKNKDYNNCEMNISSYKDATDPNVMLSVECFHSWAKNLLI
DTIRWEHQLKNCINNTNVTDCKSKCNSNCQCYEKWIKEKEKEWPQVKGVLKKKDET
SHNYYDKLKDVFDRFLFEVKGALDQDEKGKWDQFTKDLEKKFGPSVESAGTANSQD
AIELLLDHLNDNAITCKDNNSLKEDKNC>CIDRa1.2 HB3var1csa_HB3
PDCGVKCDGIKYTHKSDNDRERVNNEDYKPPWGVKPTNITVLYSGNEQGDITQKLEN
FCNSSTNYKDKNNQKWECYYKDENINRCKLEQNTEINNDNPKIISFHNFFELWVTYLL
RDTIKWNDKLKTCINNTTTHCIDECNRNCLCFDRWVKQKEEEWNSIKKLFTKKKNM
QQSYYSNINNLFEGYFFKVMDKLDKNEAKWKELMENIKKKKNEFSNLKNNRDYLEN
AIELLLDHLKETSTICKDNNTNEAC
>CIDRa1.3 PFE1640w_3D7
PHCEVDCENGNCEVKNKPDGNCGKNVKYKPPYGVKPTEITVLYSGNEKGDISKKLSEF
CSNKNNINVKNNETWKCYYKNSDNNKCKMESNSENNKGAEKITSFHEFFELWVKNL
LKDTMKWENEIKDCINNTNITDCNDECNKNCVCFDKWVKQKEEEWKNVKKVFENK
KYIQDKYYLDINKLFESFLFKVISELDQGEAKWNQLKEELKKKIESSKANEGIKDSESAI
ELLLDHLKESATTCKDNNANEAC
>CIDRa1.4 DD2var32_DD2
PDCGVECNKGTCKKKPNDSNCRNNEAYIPPRDVTPTKINVLYSGDGHGDITKKLKGFC
SNPTDYDGKNYENWQCYYKSSEDNKCQMTSLSQTLQKHHYVMSFYAFFDFWVRKFL
IDTIKWENELKNCINNTTTDCSDGCNKHCVCFDKWVKQKEKEWNSIKKLFTKKNNV
PQPYYTNINNLFDSFYFQVMYELNHDEAKWKELTQELRNKINSSKKNTDAADSKDAI
ELLLEYLKEKSTICKDNNTNEAC
>CIDRa1.4 PF11_0521_3D7
PDCGVDCSSGTCIEKKDDINCGKKINYEPPHGVKPIDIIVLYSGNEEGEITKRLSEFCTDS
SNNKGKNYEQWKCYYKNGDDNKCKMVKNSGNNITEEKIISFDEFFYVWVRKLLIDSIK
WENELNNCIDNTSTHCNKECNKNCECFDKWVKKKEDEWKNVKNVFENKNGTSHNY

YNKLNGLFKGFFFEVMDKLNKDETKWNKLIESLRTKIDSSKENIGTGNTQDTIKVLLD
HLKETATICKDNNTNEAC
>CIDRa1.5a DD2var43_DD2
PDCGVECKNGRCKKKMDTGNNCGEPYIYNIPKNVNPIGINILNSDDEHVDIVKRLSEFC
RDSKKENGKNTEIWHCYYIGDKHNQCKMEKAVAENKHQTKITTFDFFFDLWIKNLLR
DTINWKSELKNCINNKNTEKCNKECNENCKCFEKWVKQKEQEWKNVKKVFENKNG
TSQNYYNKLKSHFDNYFFLVINNVNQGEEKWKKFTDELRKKMDFSKANTGTNDAQD
SIKILLDHEKKNAGTCLENNPSEPC
>CIDRa1.7 DD2var49_DD2
PHCGVVCNNGTCRDKPNNGNCGNNVIYNPPQGVTPTNLNVLYSADQEGDISNKLSEF
CNEEIEKNSQKWQCYYVNSYINACKMEKKNGNNTSEEKITKFHNFFEMWVTYLLTET
ITWKDKLKTCMNNTKTADCIDECSTNCVCFDKWVKQKEQEWNSIKELLTEEQKNPK
QNYGNINIYFESFFFHVMKKLNKEAKWNKLMDELRNKIELSKGNEGTKDLQDAIELLL
DHLKEIATICKDNNTNEAC
>CIDRa1.1 2110-3_2110
CPDCVVECVGGECKQKTDDDKNCRSKIIEEILKGEEPTVIDVLYSGNGQGVITKKLHDF
CSSTNKEDDKYYKKWKCYNKNSDYNNCELISSLSTDPNDPNVMLSIKCFDSWARNLL
VDALKWEHQLKNCINNTNVTDCKSNCNNNCKCYEEWIKRKGREWKQVKGVIENND
EGSHYYYKNVKNLFYNFLYPVIYKLEKEEKNGKWDQFMEDLKKKIESSETNTHTTDS
QDAIELLLDHLKENAITCKDNNSLEEDKNC
>CIDRa1.1 IT4var19_IT4
PDCVVVCEGGNCKEKTEDDNCRSEIIKKILKYETPTPIDVLYSGKGQGLITKKLEDFCSS
TTNYEGTNVQKWKCYNKNKDYNNCEMNISSYKDATDPNVMLSVECFHSWAKNLLID
TIRWEHQLKNCINNTNVTDCTSKCIKNCECYEKWIKEKEKEWPQVKGVLKKKDETSH
NYYDKLKDVFDRFLFEVKGALDQDEKGKWDQFTKDLEKKFGPSVESAGTANSQDAIE
LLLDHLNDNATTCKDNNSLAVENC
>CIDRa1.4 IT4var07_IT4
PDCGVICENGKCVVKENGSNCRHYNIYEPAPDVKTTEINVIVSGDEQGIITKKLQDFCM
NPNNENGTNNQIWKCYYKDEKENKCKVETKSGNSTYKEKITSFDEFFDFWVRKLLID
TIKWETELTYCINNTTNADCNNECNKNCVCFDKWVKQKEKEWKNIMDLFTNKHDIP
KKYYLNINDLFNSFFFQVIYKFNEGEAKWNKLKENLKKKTESSKKNKGTKDSEAAIKV
LFDHLKETATICKDNNTNEAC
>CIDRa1.1 IGHvar19_IGH
PDCVVVCSNGTCSQKKDDDHCRSEIIKKILKYETPTPIEVLYSGDGQGLITEKLHEFCRG
PNKVDSKNYKTWNCYNKNNDYNKCEMISWLYEDPKKSNLMLSIQCFDSWVQNLLID
TIKWEYELKDCINNTYDADCNDECNKNCECYEKWIKQKEKEWQKVKNVFGNNNRM
SYIYYNNLSRIFDSFLFPVIYKLQKEEKDGKWDQFTKDLKKKFESSETNTPTGNSQDAI
EFLLDHLKDNATICKDNNTNEAC
>CIDRa1.1 PFCLINvar30_PFCLIN
PDCVVKCDGKTCEQKKDDENCRSKIIQKILQGEEPTVIDVLYSGKGQGLITKKLHDFCS
STNKEDDKYYKKWKCYNKNSDYNNCELISSLSTDPTDPKVMLSIKCFDSWARNLLVD
ALKWEHQLKNCINNTNVTDCKSNCNNNCKCYEEWIKQKEKEWQKVKGVLKKKDKN
SDNYYKNVKNLFYSFLFQVIYELEKEEKNGKWDQFMEDLKKKIEASQKNKGTENSQD
AIELLLDHLKDNATICKDNNTNEAC
>CIDRa1.1 RAJ116var08_RAJ116
PDCVVDCTGGNCKENKKHDNCRSKIIEEILRSEETTDIDVLYSGKGPGAITKKLHDFCS
NPNNYKRANVQKWKCYNKNKDYNNCEMNISSYKDATDPNVMLSVQCFYSWAQNLL
IDIIRWEHQLKDCINNTNVTDCDNECNKNCECFEKWIKQKQKEWQQVKEVLKKKDE

NSHNYYNKLRSVFDSFLYQVMFALNNEEKGKWDQFTEDLEKKFESSKKSAGTGNSQD
AIEFLLDHLNDNAITCKDNNSKESC
>CIDRa1.1 GA011_ERS009959
PDCVVYCEGGECKENENGDNCRSEIIKEIVRSETPTVIDVLYSGNGQDHITEKLDHFCSS
TNKEDRKYYKTLKCYNKNNDYNKCEMISWLYQDPKESNLMLSIQCFYSWAQNLLIDT
IRWEHQLKDCINNTNVTDCKSNCNKNCKCYKKWIEQKGSEWQQVKQVLKKKDKNSE
NYYDKLKDVFDTLYFEVMHELNKGKEKGKSEELTEDLKKKFGPSKKNTDAADSKDAI
EFLLDHLNDNAITCKDNNSLAVENCTRTKSNPC
>CIDRa1.5a 1965-2_1965
PDCGVECKNGRCKKKTDADGNCGKTQIYDIPKDVTPTDINVLYSGDEYGDIAKRLSEFC
RDSKKENGKNTEIWHCYYIGDKHNQCKMEKAVAENKHQTKITTFDFFFDLWIKNLLR
DTINWKSQLKNCINNTNKTNCNKTCNENCKCFENWVNQKEKEWNNMKELFKNKN
RTSQNYYNKLKSHFDNYFFLVINNVNKGEAKWKKFTDELRNKIDSSKAKNGTSDSQD
SIKMLLEHLKEDGTTCTANNPDSICNTPGTDARSLEPASKIEPQNKDTRTNPC
>CIDRa1.5a GA013_ERS010323
PYCGVDCSNGTCKENGNDDNCGEQQNYNIPKGVDPTNINVLYSVDGHGDIAKRLSTFC
NDKNNKNAKNNEIWQCYYIGHKNNQCKIEKSVSQKKHQTKIAPFDYFFDLWVTNLLR
DNIDWKNELKNCINNKNTEKCNKECNDNCKCFQSWLNKKEDEWNKITGLLKNKNGI
LQNYYNKLKSHFDYYFFQVINNINKGEEKWKKLKEDLKKEIDFSKLKTNTGDSQDSIK
LLLDHENKNAGTCLKNNPIDSCPKAEPQKSDEKVQPQDTPPNSC
>CIDRa1.5a GA014_ERS010022
PYCGVDCNGTTCTPKTVIYPDCGNNDVYEPPRGVTPTDINVLYSGDEYGDIAKRLSEFC
NDKNNKNGKKNETWKCYYENSEKNMCKMDKNSKNHTSEEKITSFDYFFDLWVTNL
LRDTINWKNDLMNCINNKKMKNCNKTCNDNCKCFQSWLNKKEDEWNKITGLLKNK
NRTSQNYYNKLNNHFQGYFFQVTNEVNKDEAKWNQFTEELRKKMDFSKANTGTND
SQDAIKVLLEHLKEDGKTCTANNPDSACNNPKAARIIIRATTRNPC
>CIDRa1.5b 1918-5_1918
PDCGVKCTNGKCEEKTDVDGNCGNKETYIPPRDISPTEISVLYSGYKRDDISEKLETFC
REPTNNKSKNNETWKCYYKHSYNNKINKCIRQNEENIKNKLIINLDTFFEFWVRSFLN
DTIDWKYDLNTCMNFTNTTKCNNNCNKNCKCFEQWVNKKETEWKNVAQYFFKHN
EISKKKYCEILKDIFENYYVEVIKKVFKGDNKWKEIMVDIKNKIDCSNLKNGTEHLEKII
NVLINQEQADATTCLKNNPIESCPKAEPQKSDENTQPQDTPPNSC
>CIDRa1.5b 1983-13_1983
PDCGVECNGKTCTKKEETDENCGKPPNYTIPTDVTPTDINVLYSGDEQGYITKRLSEFC
GPTKNYIGKKNELWKCYYDDKQKNNNNNNNICKLQPNAHIKNENIIINFDNFFEFWV
RRFLNDTIDWKYDLNTCMNFTNTTKCNNNCNKNCKCFEQWVNIKKTEWKNVTEYF
FKHDETSKKKYCEILKDIFENYYVEVIEKFYKGKHKWKERIEKLKNMDCSQVKIGTNA
SQDQIDIFLSNLKGDATQCTSKNPKSACNKPHAARIITRATTRNPC
>CIDRa1.5b GA017_ERS010601
PDCGVECTKGRCEKKAETDDNCGKPPIYTIPTDVTPTDINVLYSGDEQGYITKRLSKFC
GPRKNYIGKKNELWKCYYDDKHNNKDNICITDTNQKITNRYIMNFDNFFEFWVRRLL
IDTINWEYKLKTCIDNNNNDKCISGCNEDCKCFDKWVDQKEKEWKSVKKHIAKEKEI
GKNKYCEKLKKIFSDYYVQVIETIYKGKHKWKRRIESIKKIDCSQVKIGNKDSEYQIDTF
FRSVKEGATQCTSKNPKSACDKPKAARIITPATTRNPC
>CIDRa1.6b GA018_ERS010570
PYCGLDCVGTECTAKEEIYPDCVYNGEYDPPKDVRPTEINVIDSGNAVNISEKLNDFCT
NPTNPNDKIYQKWQCYYKSSKVNKCQMTSLTQTLQKHHYVMTFYIFFDLWVKNLLR
DSVKWEIELKDCINNTNVTDCNNKCNKNCVCFDKWVKQKKKEWDSIKKLFKTEKDE

MKKYYTNINKNFEFFFFRVMYELNNEEAKWNKLMENLRIKIKSSKRNKRTKDSEGVI
KVLFDHLKETATICKDNNTNEACVSSQNATTNPC
>CIDRa1.6b GA019_ERS010031
PYCGLDCVGKTCTAKQEIYPDCVYNGDYEPPNGAETTEITVLYNDNEGDMSKKLSQFC
SNENKENIENYQKWKCYYKDRDDIECEMISSSQKDEKHRKVMIFYNFFDLWVKNLLR
DSIKWEIELKDCINNTNVTDCKSVCNVNCECFDKWVKQKEKEWDSIKKLLKKKKNVS
KKYYTNINKNFEFFFFRVMYELNNEEAKWNKLMENLRTKINSYKKNKRTKDSEGAIK
VLFDHLKETATICKDNNTNEACVSSQNATTNPC
>CIDRa1.7 IT4var22_IT4
PYCGLDCGGKTCTAKQEIYPDCVYNGAYEPPNGAETTEITVLYSADQEGDISNKLSEFC
NDENNKNSQKWQCYYVSSENNGCKMEKKNANHTPEVKITKFHNFFEMWVTYLLTE
TITWKDKLKTCMNNTKTADCIHECNKNCVCFDKWVKQKEDEWNSIKKLFTKEKKM
PKQYYGNINIYFESFFFHVMKKLNKEAKWNKLMDELRNKIELSKGNEGTKDLQDAIEL
LLEYLKEKSTICKDNNTNEACDPTVDPTKNPC
>CIDRa1.7 1965-8_1965
PHCGVDCNGKKCTLKSDNDPQCVNKLKYEPPYGVKPTEITVLYSADQEGDISKKLSEF
CNDEKKINSKNIETWKCYYKSTYNNACKMDKNSKNHTPEVKITKFHNFFEMWIVYLL
TETITWNDKLKTCMNNIKTTDCIDECNTNCVCFDKWVKQKGKEWNSIKKLFIKEQKN
PKQNYGNINIYFESFFFHVMDKLNHDEAKWNELMNELKQIIDSSKANTDNKNLQDAI
KVLFDHLKEIATICKDNNTNEACEDSKKETQNPC
>CIDRa1.4 1918-3_1918
PDCGVECNGETCTPKKEKYPECLNKEIYTPNGAKTTEINVIVSGNEQGDITKKLEDFCN
NSTNYKGKNYQKWQCYYENSKKNMCKMDKNSKNHTSEEKITKFHNFFELWVIYLLT
ETIRWNDKIKNCINNTTIHCIDKCNKNCVCFDKWIKQKEQEWNSIKKLFIKKQKMPN
EYYLNIKNHFEGYFFHVMKKLNKEAKWNELMENLRTKINSSKKNKGTKDSEGAIKVL
LDHLKETATICKDNNTNEGCVSSKKSKTNPC
>CIDRa1.7 1914-14_1914
PDCGVQCNGEKCKEKEYDHECENDEDYGLPSDVTPIDITVLYSGNEAGDISEKLKDFC
NISTKYEGKNYENWQCYYKNKNKNKCKMEQNSKKDKDKPKITKFHNFFELWVIYLL
TETIRWNDKIKNCMNNTNITDCSDGCNKHCVCFDKWVKQKEEEWNSIKKLFIKEQE
MPNEYYLNIKNHFEGYFFHVMDKLNHDEAKWKELTQELKKKIDSSKEKPGTKDSESA
IELLLDHLKEIATICKDNNTNEACASSKKSKTNPC
>CIDRa1.4 GA024_ERS010438
PDCGVECTNGRCEKKAETDGNCGNKETYKPPHGVEPIDITVLYSGNEEREITKRLSEFC
TDSSNNKGKNYENWQCYYKNGDDNKCQMTSLTQTDEKHRYVMTFHKFFNFWVRNL
LIDTINWETDLRNCLNNTGITDCNDGCNNNCTCFDKWVKQKEGEWNSIKLLLAKEQ
KMPKKYYLNINDLFDSFFFEVMDKLNKDETKWNKLKENLKKKIKSSNENRGTKDSES
AIELLLDHLKEIATICKDNNANEACETSRNRKTNPC
>CIDRa1.8a GA026_ERS010178
PDCVVDCKGAKCEQKMKPDGTCEKPQIYTRPKDVTPKIIKVLFSGDNQEDITEKLSSFC
SNPKSKIDRNYQTWKCYYKSGHYNICEMKGSLYKYPEHPNDMLSVECFHLWVKNLLI
DTIKWETKLKKCINNTNVTDCDNECNKNCECFENWVEQKKKEWEEVRKVYKDQKE
SLGIYYKKLENLFDTYYFEVMGALENEEKHGKWNQLTAKLKQIIESHKKNRHSGNSQ
DAIELLLDHLKETATTCKDNNSNESCDVSKDSKTNPC
>CIDRa1.8b 2053-3_2053
PICGVKCENKSCTDKENDDDCKNKKKYDPPKGVTPIDIPILYSGDKQGDITKKLEDFCY
NRTKENEKTYQNWKCYYKDSEFNKCKMESKSGKSTTQEKIISFDEFFYLWVNNLLIDS
IMWENDIKHCINNTNVTNCKNKCNENCKCFKNWVKKKEEEWTKVKQILGNRSENLN

NYYNKLNSLFKGFFFEVVYKFNNKEEKWNKLTEKLEQKIGSSKGKEGVENPKDAIELL
LDHLKENAITCKDNNSLEEDKNCPKIKINPC
>CIDRa1.8b 1702-3_1702
PDCIVVCDYKGCKENKNDENCKSKRTYSLPPDVNSTEIEVLFSGDNQEDIVEKLSSFCK
NTNNENGENVEKWECYYESEHNNKCKMTSPKHKDEKRPTVMIFDEFFDFWVTHLIK
DTIKWESDINDCINNTNVTDCDSACNENCKCFDEWVEKKEVEWGNVKKVLGNRSEN
LNIYYNKLNGIFSGFFFGVMHELKKKEAKQGVKAEEAQEAEETQEAQEEEAKWNKLT
AKLQEIIDSSKGKADTANSQDAIKPLLEYIKETATTCIDNNSLAVENCPKTKINPC
>CIDRa1.8b GA029_ERS010532
PDCVVVCERGNCTEKKGDDKCRKKYTYEIPAGMESTKIDILFSGENQEDITEKLRSFCK
NTNNENGENIEKWECYYKNEYDNKCKMTSLKREDQKHHDLMSYYEFFDFWVTHLIK
DTIKWESDINDCINNTNVTNCNNGCNENCICFEKWVGQKEKEWENVKKVLKNPSEN
LNNYYNKLNGIFSGFFFGVMHELKKKEAKQGVKVEKAEKSEEAQEAQEEEAKWNKLT
AKLEEIIKSHKENTGTGNTQDAIEPLLKYLDENAITCKDNNSLKEDKNCPKTKKNPC
>CIDRa1.6a PF08_0140_3D7
PDCGVEYKNGRYTAKDQKYPDCRNEKYDPNNAETTDITVLYSGDVGDFSEKLQDFCN
DINNGKVKNYQIWQCYYENSEINKCQMTPSSHKVPKHGYIMSFFAFFDLWVKNLLIDT
INWKNELTNCINNTNVTDCKNDCNTNCKCFENWAKTKEKEWENVKTIYKNENRNT
NNYYKKLNDLFKGYFFHVMYELNNEEAKWNKLMKNLRTKIDSSRKNAGNEDSEGAI
KVLFDHLKDIAERCIDNNSIKPC
>CIDRa1.4 GA045_PREICH
PECGVKCEHGSCRDKPKDDHCINKNEYDPPRDVTPTNITVIYSGNEEGEITKRLSAFCT
DSSKDEGKNYQKWECYYVNSDSNRCKMDKTSGKNMTEDKITSFDEFFYSWVQNFLID
TIKWENELNNCINNTILTDCNDGCNSNCVCFDKWVKKKENEWKKVKNVFENKSGIS
DNHYKKLNGLFDSFFFPVMHALNEQEKRKWKELTKKLEEISGFSKGKTGTVNSQDTI
KVLLDHLKENATICKDNNTNEAC
>CIDRa3.1 DD2var01_DD2
PYCGVKKVNNGGSSNEWEEKNNGKCKSGKLYEPKPDKEGTTITILKSGKGHDDIEEKL
NKFCDEKNGDTINSGGSGTGGSGGGNSGRQELYEEWKCYKGEDVVKVGHDEDDEEDY
ENVKNAGGLCILKNQKKNKEEGGNTSEKEPDEIQKTFNPFFYYWVAHMLKDSIHWK
KKLQRCLQNGNRIKCGNNKCNNDCECFKRWITQKKDEWGKIVQHFKTQNIKGRGGS
DNTAELIPFDHDYVLQYNLQEEFLKGDSEDASEEKSENSLDAEEAEELKHLREIIESED
NNQEASVGGGVTEQKNIMDKLLNYEKDEADLCLEIHEDEEEEKEKGDGNECIEEGENF
RYNPC
>CIDRa3.5 IT4var15_IT4
PGCGVELIGNEWKEKNKGECKGGKRYNIPKGTKHNVIPVLSFGDEHKEIIEKIEQFCAE
SNSDSSKLTEQWKCYYGDKEYEVCTLENRNKSEEDPEEIQKTFHNFFYFWIRHLLNDS
IEWRDKINNCIEKAKEGKCKNECKTDCGCFQRWIGKKKEEWGEIKKHFKTQDGFSIFG
NNYDFVLENVLNIDELFQDITEAYGNSQKIQGIKDTLAKKKTQAADDATEQKNTIDLL
FEYDSEEAEKCKKIQEECQPKKPTKVRNPC

Supplemental figure legends:

Supplemental Figure 1 – Binding of CIDRα1 variants to EPCR – related to Figure 1

A. ELISA was used to study binding of 40 recombinant CIDRα1 and two CD36-binding CIDRα3 variants binding to EPCR and CD36. Domains were classified as EPCR binders if OD > 0.1. **B.** Surface plasmon resonance was used to quantify the binding of CIDRα1 domains to an EPCR coupled surface. EPCR was coupled by capture using a biotin attached to a BAP tag at the N-terminus and the chip was regenerated and coupled with fresh EPCR after each cycle. CIDRα1 domains were injected at concentrations described in Table S2.

Supplemental Figure 2 – Structural and biophysical characterisation of the CIDRα1:EPCR interaction – related to Figure 2

A. An isothermal titration calorimetry experiment was carried out using a MicroCal iTC200 machine in which HB3var03 CIDRα1.4 domain was placed into the cell and subjected to injections of 36 μM EPCR at 25°C. **B.** Multi-angle laser light scattering (MALLS) measurements of HB3var03 CIDRα1.4 (red, MW=26.3 kDa), EPCR (orange, MW=22.1 kDa) or the HB3var03 CIDRα1.4:EPCR complex (purple) show the formation of a 1:1 complex with a mass of 47.8 kDa . **C.** Equilibrium analytical ultracentrifugation of the HB3var03:EPCR complex revealed a mass of 47.1 kDa, demonstrating formation of a 1:1 complex. **D.** The theoretical scattering calculated from *ab initio* reconstructions (yellow is for HB3var03 CIDRα1, red for EPCR and blue for the CIDRα1:EPCR complex), superimposed into experimental scattering data. Guinier plots are superimposed. **E.** Distance distribution functions of HB3var03 CIDRα1 (yellow), EPCR (red) and the CIDRα1:EPCR complex (blue), derived from small angle x-ray scattering. **F.** The structures of HB3var03 CIDRα1, EPCR and their complex, docked into *ab initio* molecular envelopes calculated from scattering data. **G.** The theoretical scattering calculated from *ab initio* reconstructions (blue is DD2var32 DBLα1.7-CIDRα1.4-DBLβ1, red for EPCR and green for the DD2var32:EPCR complex), superimposed into experimental scattering data. Guinier plots are superimposed. **H.** Distance distribution functions of DD2var32 DBLα1.7-CIDRα1.4-DBLβ1 (blue), EPCR (red) and the DD2var32:EPCR complex (green), derived from small angle x-ray scattering. **I.** A representation of the HB3var03:EPCR crystal structure (molecules A and B), with the thickness of the wire representing the B factors associated with the residue showing the interface between HB3var03 CIDRα1.4 and EPCR to be ordered while loops from the CIDRα1.4 domain are flexible. **J.** The electron density, contoured at 1.0 σ for the residues from HB3var03 CIDRα1.4 that form the helix and kinked helix at the heart of the EPCR binding site.

Supplemental Figure 3 - A sequence logo describing conservation across the EPCR-binding CIDRα1 domains – related to Figure 2

All sequences of CIDRα1 subclasses 1.1 and 1.4-8 were aligned and a sequence logo generated (of positions corresponding to residues of the HB3var03 CIDRα1 domain). Residue positions are numbered according to the position in HB3var03. Below the logo is shown the secondary structure in HB3var03. Size polymorphisms are indicated >< for deletions and <> for insertions (see table for details). Residues that make direct contact with EPCR are annotated with * above the logo. Cysteines are labelled, with C5-C12, C6-C7, C8-C10, C11-C13 forming disulphide bonds in HB3var03.

Supplemental Figure 4 – Binding of HB3var03 CIDRα1 mutants to EPCR – related to Figure 3

Surface plasmon resonance was used to quantify the binding of mutants of the HB3var03 CIDRα1.4 domain to an EPCR coupled surface. EPCR was coupled using a biotin attached to a BAP tag at the N-terminus and the chip was regenerated and coupled with fresh EPCR after each cycle. CIDRα1 domains were injected at concentrations described in Table S6.

Supplemental Figure 5 – Sequence diversity of the nine EPCR interacting residues of CIDRα1 domains

A maximum likelihood tree built on the nine residues that directly interact with EPCR forms subgroups similar to trees built on the whole domains. Branches are coloured according to the sub-classification of the whole domain given in Figure 1. Sequence logos for each CIDRα1 subclass show the diversity of the residues making direct contacts with EPCR.

Supplemental Figure 6 – **Human IgG pool reactivity to recombinant CIDRα1 domains – related to Figure 6**

A. Two human IgG pools were made from human plasma samples selected after screening for reactivity to either a UPSA CIDRα1 (HB3var03 CIDRα1.4) or a UPSB CIDRα1 (IT4var20 CIDRα1.1) as described in materials and methods. From the pools, IgG was affinity purified on 63-amino acid peptides corresponding to the EPCR binding region of either HB3var03 or IT4var20 CIDRα1 domains. The graph shows CIDRα reactivity of the IgG pools prior to purification, the purified IgG pools and the run-through IgG (that did not stick to the peptide) pools. **B.** The reactivity of IgG purified on the HB3var03 CIDRα1 domain was tested against activated protein C, showing negligible cross reactivity.

Supplemental references:

Altschul, S. (1997). Gapped BLAST and PSI-BLAST: a new generation of protein database search programs. Nucleic Acids Res. 25, 3389–3402.

Crooks, G.E., Hon, G., Chandonia, J.-M., and Brenner, S.E. (2004). WebLogo: a sequence logo generator. Genome Res. 14, 1188–1190.

Edgar, R.C. (2004). MUSCLE: multiple sequence alignment with high accuracy and high throughput. Nucleic Acids Res. 32, 1792–1797.

Franke, D., and Svergun, D.I. (2009). DAMMIF , a program for rapid ab-initio shape determination in small-angle scattering. J. Appl. Crystallogr. 42, 342–346.

Higgins, M.K. (2008) The structure of a chondroitin sulfate-binding domain important in placental malaria. J Biol Chem. 283, 21842-6.

Konarev, P. V., Volkov, V. V., Sokolova, A. V., Koch, M.H.J., and Svergun, D.I. (2003). PRIMUS : a Windows PC-based system for small-angle scattering data analysis. J. Appl. Crystallogr. 36, 1277–1282.

Miotto, O., Almagro-Garcia, J., Manske, M., Macinnis, B., Campino, S., Rockett, K.A., Amaratunga, C., Lim, P., Suon, S., Sreng, S., et al. (2013). Multiple populations of artemisinin-resistant Plasmodium falciparum in Cambodia. Nat. Genet. 45, 648–655.

Shi, J., Blundell, T.L., and Mizuguchi, K. (2001). FUGUE: sequence-structure homology recognition using environment-specific substitution tables and structure-dependent gap penalties. J. Mol. Biol. 310, 243–257.

Svergun, D.I. (1992). Determination of the regularization parameter in indirect-transform methods using perceptual criteria. J. Appl. Crystallogr. 25, 495–503.

Svergun, D.I. (1999). Restoring low resolution structure of biological macromolecules from solution scattering using simulated annealing. Biophys. J. 76, 2879–2886.

Tamura, K., Stecher, G., Peterson, D., Filipski, A., and Kumar, S. (2013). MEGA6: Molecular Evolutionary Genetics Analysis version 6.0. Mol. Biol. Evol. 30, 2725–2729.

Vistica, J., Dam, J., Balbo, A., Yikilmaz, E., Mariuzza, R.A., Rouault, T.A., and Schuck, P. (2004). Sedimentation equilibrium analysis of protein interactions with global implicit mass conservation constraints and systematic noise decomposition. Anal. Biochem. 326, 234–256.

Volkov, V. V., and Svergun, D.I. (2003). Uniqueness of ab initio shape determination in small-angle scattering. J. Appl. Crystallogr. 36, 860–864.

Wernersson, R. (2006). Virtual Ribosome--a comprehensive DNA translation tool with support for integration of sequence feature annotation. Nucleic Acids Res. 34, W385–8.

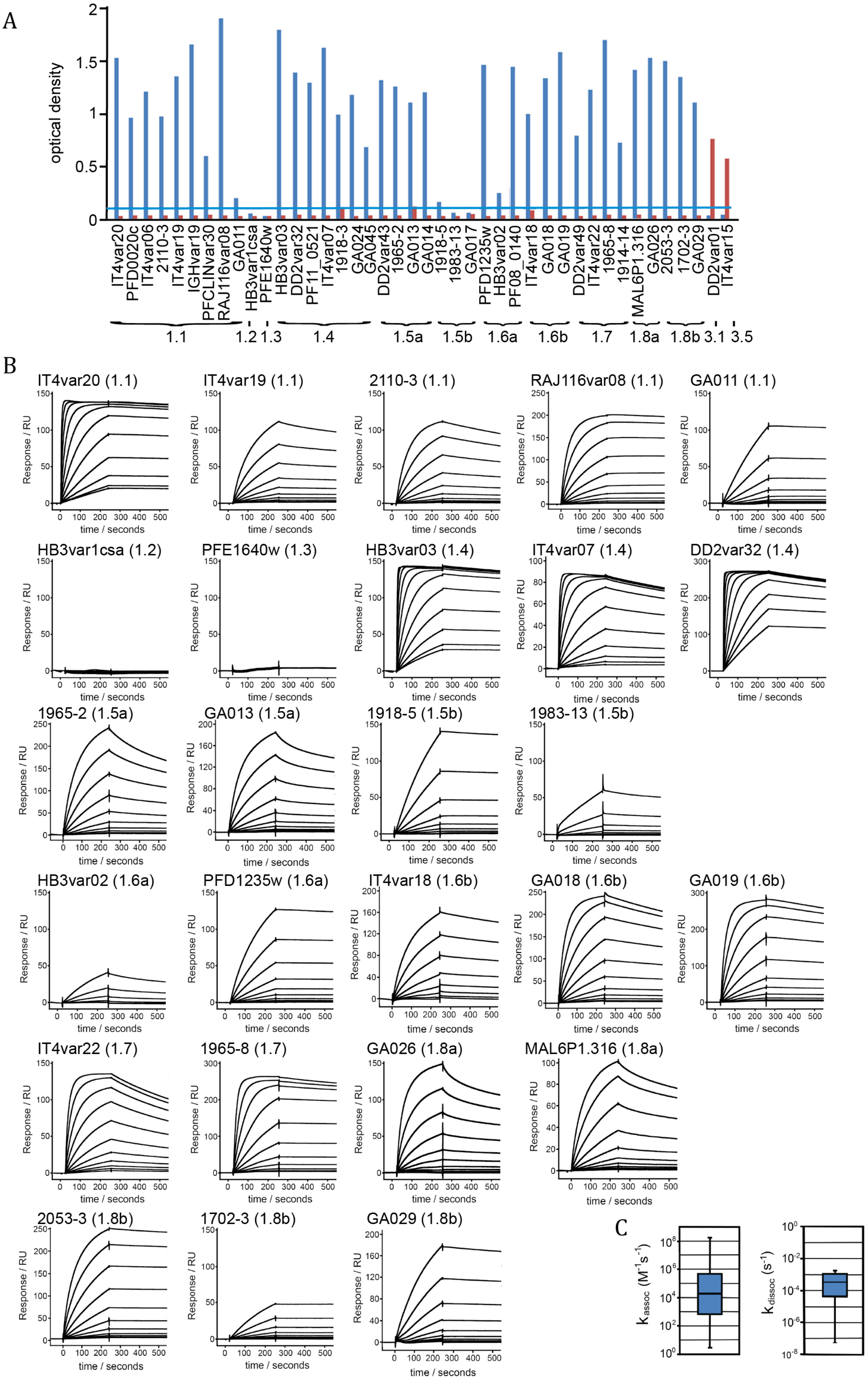
A
B
C
optical density
IT4var20 (1.1)
IT4var19 (1.1)
2110-3 (1.1)
RAJ116var08 (1.1)
GA011 (1.1)
HB3var1csa (1.2)
PFE1640w (1.3)
HB3var03 (1.4)
IT4var07 (1.4)
DD2var32 (1.4)
1965-2 (1.5a)
GA013 (1.5a)
1918-5 (1.5b)
1983-13 (1.5b)
HB3var02 (1.6a)
PFD1235w (1.6a)
IT4var18 (1.6b)
GA018 (1.6b)
GA019 (1.6b)
IT4var22 (1.7)
1965-8 (1.7)
GA026 (1.8a)
MAL6P1.316 (1.8a)
2053-3 (1.8b)
1702-3 (1.8b)
GA029 (1.8b)
Response / RU
time / seconds

Figure S1

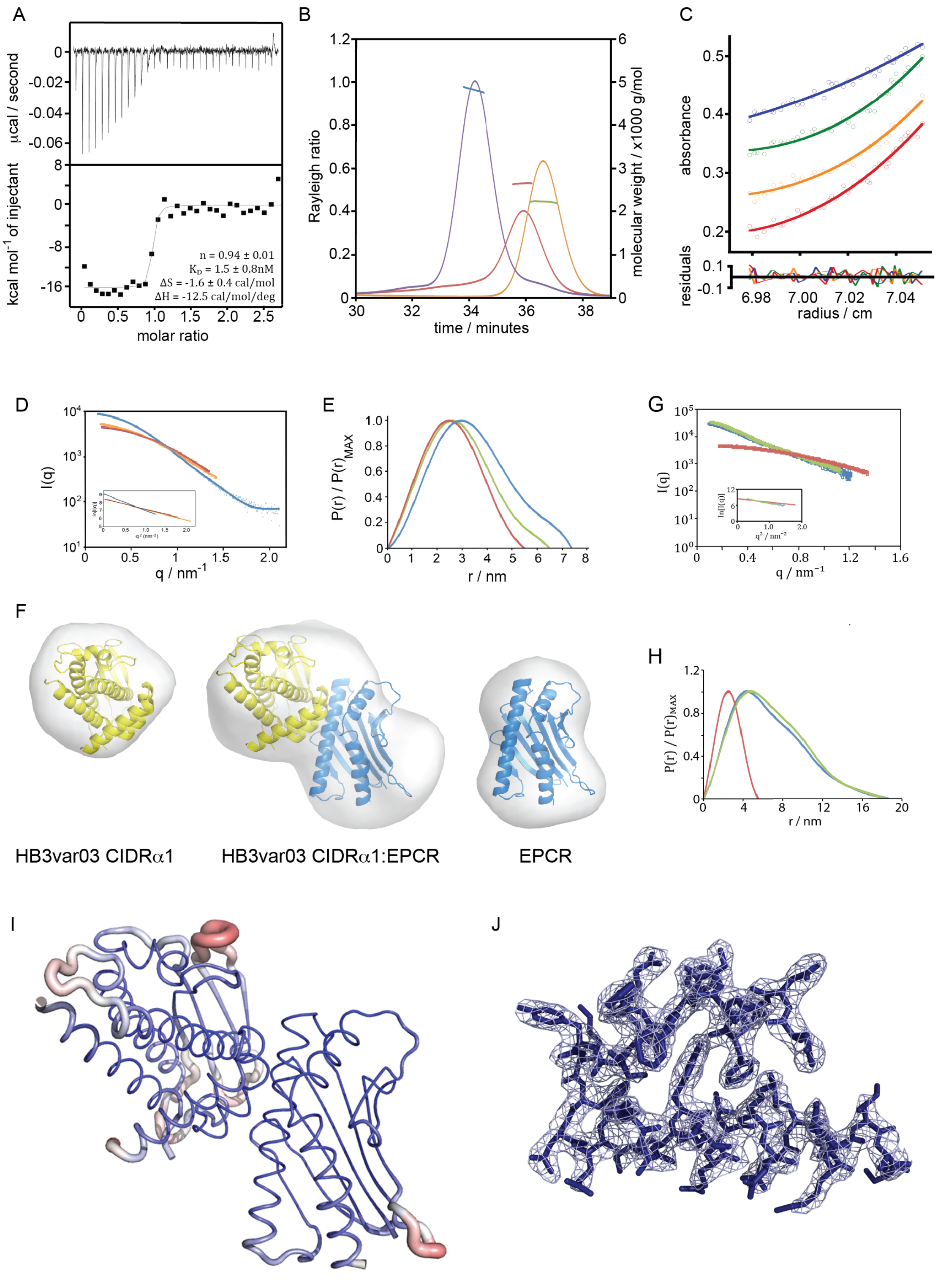

A
µcal / second
kcal mol-1 of injectant
molar ratio
n = 0.94 ± 0.01
KD = 1.5 ± 0.8nM
ΔS = -1.6 ± 0.4 cal/mol
ΔH = -12.5 cal/mol/deg
B
Rayleigh ratio
molecular weight / x1000 g/mol
time / minutes
C
absorbance
residuals
radius / cm
D
I(q)
q / nm-1
E
P(r) / P(r)MAX
r / nm
G
I(q)
q / nm-1
F
HB3var03 CIDRα1
HB3var03 CIDRα1:EPCR
EPCR
H
P(r) / P(r)MAX
r / nm
I
J

Figure S2

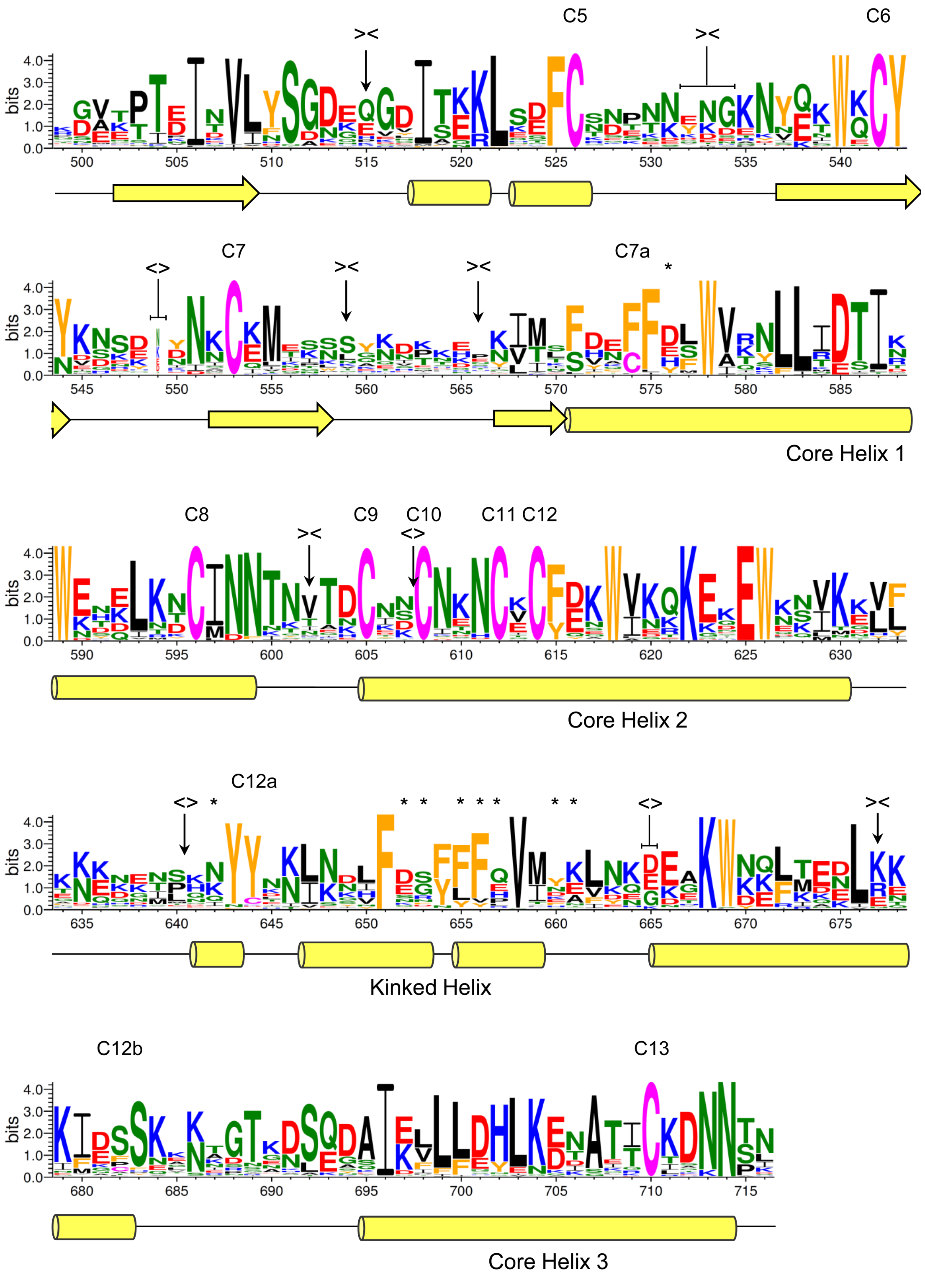
C5
C6
C7
C7a
Core Helix 1
C8
C9
C10
C11 C12
Core Helix 2
C12a
Kinked Helix
C12b
C13
Core Helix 3
bits

Figure S3

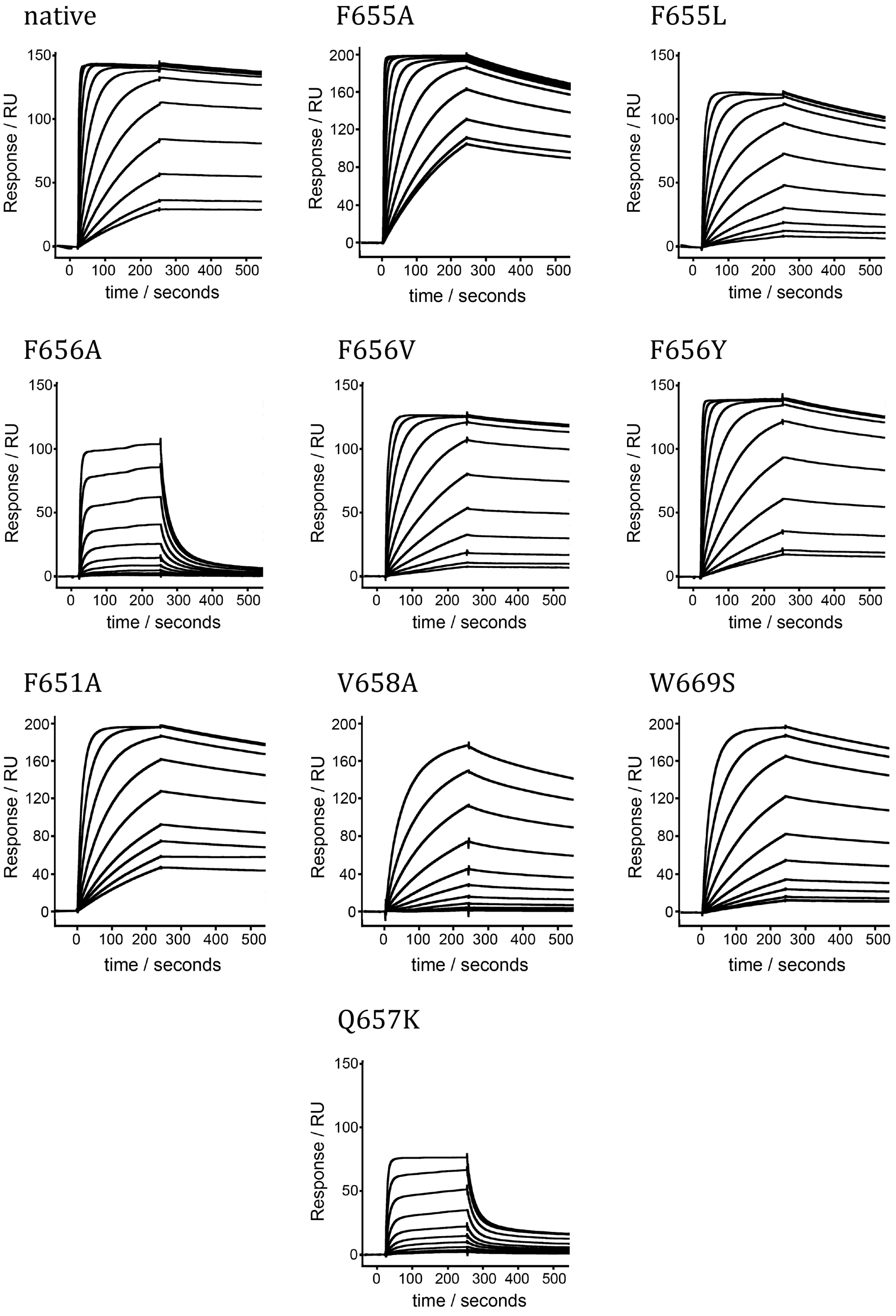

native
F655A
F655L
F656A
F656V
F656Y
F651A
V658A
W669S
Q657K
Response / RU
time / seconds
0
50
100
150
40
80
120
160
200
0 100 200 300 400 500

Figure S4

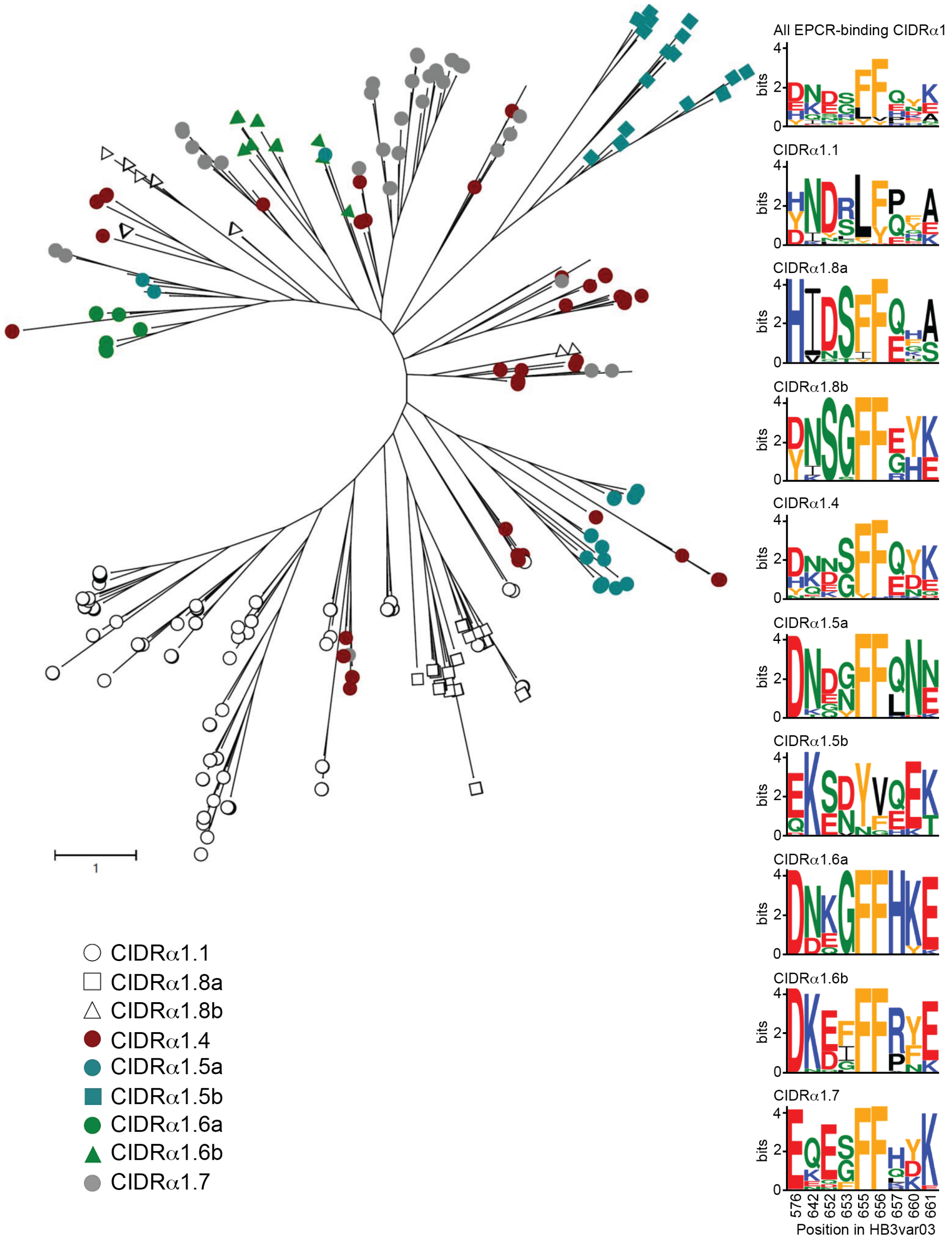
All EPCR-binding CIDRα1
CIDRα1.1
CIDRα1.8a
CIDRα1.8b
CIDRα1.4
CIDRα1.5a
CIDRα1.5b
CIDRα1.6a
CIDRα1.6b
CIDRα1.7
bits
576 642 652 653 655 656 657 660 661
Position in HB3var03
1
CIDRα1.1
CIDRα1.8a
CIDRα1.8b
CIDRα1.4
CIDRα1.5a
CIDRα1.5b
CIDRα1.6a
CIDRα1.6b
CIDRα1.7

Figure S5

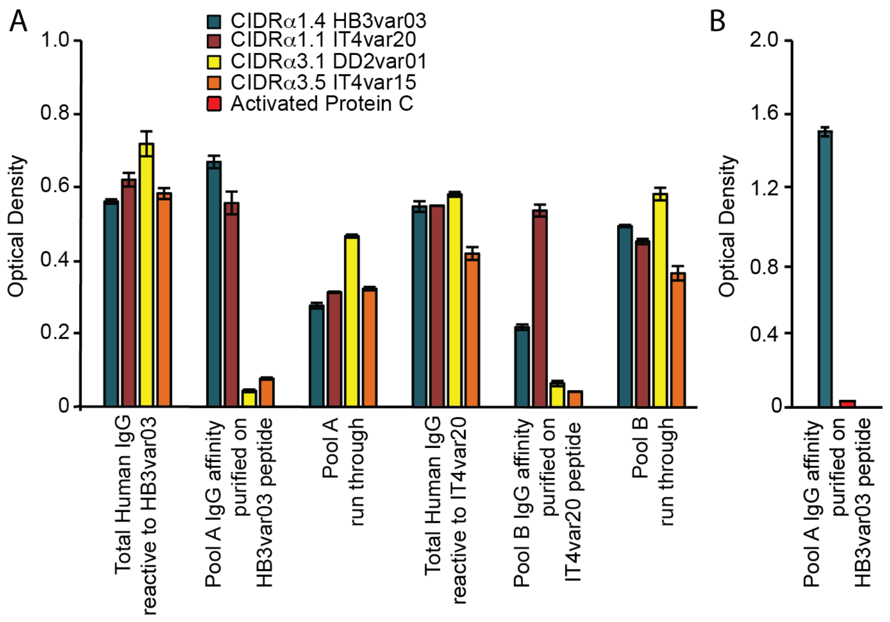

A
B
CIDRα1.4 HB3var03
CIDRα1.1 IT4var20
CIDRα3.1 DD2var01
CIDRα3.5 IT4var15
Activated Protein C
Optical Density
1.0
0.8
0.6
0.4
0.2
0
2.0
1.6
1.2
0.8
0.4
Total Human IgG reactive to HB3var03
Pool A IgG affinity purified on HB3var03 peptide
Pool A run through
Total Human IgG reactive to IT4var20
Pool B IgG affinity purified on IT4var20 peptide
Pool B run through

Figure S6

Table S1: Distribution and pairwise sequence similarity of the analysed CIDRα1 subclasses – related to Figure 1

Based on the maximum likelihood tree in Figure 1A we annotated the 885 CIDRα1 domains utilized for sequence variation analysis. §Redundancy was evaluated from the amino acid sequence. *Three small clusters of sequences of intermediate subtypes with highest resemblance to 1.4, 1.7 and 1.6a subtypes, respectively.

Subtype	Total obs.	Non-redundant sequences§	Sequence pair wise identity (%)		
			Minimum	Average	Median
CIDRα1.1	168 (19%)	124 (74%)	50	68	67
CIDRα1.2	86 (10%)	25 (29%)	57	94	95
CIDRα1.3	62 (7%)	12 (19%)	56	86	100
CIDRα1.4	130 (15%)	109 (84%)	40	59	57
CIDRα1.5a	74 (8%)	54 (73%)	45	66	64
CIDRα1.5b	69 (8%)	50 (72%)	40	63	65
CIDRα1.6a	33 (4%)	25 (76%)	58	81	83
CIDRα1.6b	41 (5%)	28 (68%)	54	70	67
CIDRα1.7	104 (12%)	75 (72%)	51	68	66
CIDRα1.8a	48 (5%)	41 (85%)	59	74	71
CIDRα1.8b	32 (4%)	24 (75%)	49	69	69
Unclassified*	38 (4%)	35 (92%)	-	-	-
All	885	602 (68%)	27	49	46

Table S2: Affinities of CIDRα1.1 domains for ECPR as measured by surface plasmon resonance – related to Figure 1

CIDR	Subclass	Range	K_D (nM)	k_{on} ($M^{-1}s^{-1}$)	k_{off} (s^{-1})
IT4var20	1.1	1uM-0.9nM	0.37	3.32×10^{5}	1.23×10^{-4}
IT4var19	1.1	0.5uM-0.5nM	16	2.27×10^{4}	3.64×10^{-4}
2110-3	1.1	1uM-0.9nM	32	1.57×10^{4}	5.11×10^{-4}
RAJ116var08	1.1	1uM-0.9nM	1.4	2.79×10^{4}	3.85×10^{-5}
ERS009959	1.1	8uM-7.8nM	182	3.89×10^{2}	7.24×10^{-5}
HB3var1csa	1.2	1uM-0.9nM	-	-	-
PFE1640w	1.3	1uM-0.9nM	-	-	-
HB3var03	1.4	1uM-0.9nM	0.37	5.32×10^{5}	1.97×10^{-4}
IT4var07	1.4	1uM-0.9nM	1.3	3.70×10^{5}	4.68×10^{-4}
DD2var32	1.4	1uM-0.9nM	0.3	1.06×10^{6}	3.43×10^{-4}
1965-2	1.5a	1uM-0.9nM	54	1.89×10^{4}	1.03×10^{-3}
ERS010323	1.5a	1uM-0.9nM	57	1.47×10^{4}	8.32×10^{-4}
1918-5	1.5b	8uM-7.8nM	229	4.71×10^{2}	1.08×10^{-4}
1983-13	1.5b	8uM-7.8nM	168700	2.8	4.70×10^{-4}
HB3var02	1.6a	8uM-7.8nM	1400	9.75×10^{2}	1.39×10^{-3}
PFD1235w	1.6a	1uM-0.9nM	8.8	7.55×10^{3}	6.62×10^{-5}
IT4var18	1.6b	2uM-1.8nM	56	5.62×10^{3}	5.63×10^{-8}
ERS010570	1.6b	1uM-0.9nM	15	3.51×10^{4}	5.31×10^{-4}
ERS010031	1.6b	1uM-0.9nM	8.8	3.70×10^{4}	3.28×10^{-4}
IT4var22	1.7	1uM-0.9nM	17	6.13×10^{4}	1.05×10^{-3}
1965-8	1.7	1uM-0.9nM	3.5	5.92×10^{4}	2.10×10^{-4}
ERS010178	1.8a	1uM-0.9nM	48	1.77×10^{4}	8.42×10^{-4}
MAL6P1.316	1.8a	1uM-0.9nM	50	1.67×10^{4}	8.28×10^{-4}
2054-3	1.8b	1uM-0.9nM	4	2.02×10^{4}	7.65×10^{-5}
1702-3	1.8b	1uM-0.9nM	6	3.64×10^{3}	2.28×10^{-5}
ERS010532	1.8b	1uM-0.9nM	24.18	6.55×10^{3}	1.58×10^{-4}

Table S3: Analysis of insertions and deletions in the CIDRα1.1 domains – related to Figure 1

Size polymorphisms are indicated with >< for deletions and <> for insertions in Figure 1B. Here the details of the type, prevalence and size of these polymorphisms.

Size variation (residues)	Average	Median
199-252	218.7	218

Position	Indel	Size	Prevalence	Associated subtype
515	Deletion	1	8%	1.6
532-534	Deletion	3	6.5%	1.7
549	Insertion	1-6	15.5%	1.4/1.5b
559	Deletion	1	10%	1.5b
566	Deletion	1	6%	1.5b
602	Deletion	1	8%	1.4/1.7
607-608	Insertion	1	95%	
640-641	Insertion	1	9%	1.5b
665	Insertion	1-3	82%	
		18-35	2.3%	1.8b
677	Deletion	1	7%	1.5b

Table S4: Summary of SAXS parameters of PfEMP1 domains, alone and in complex with EPCR – Related to Figure 2

The radius of gyration (R_g) was determined from the Guinier plot, and the maximum particle dimension (Dmax) and the Porod volume were calculated using GNOM. I_0 is an estimate of the scattering intensity at zero angle. An estimate of the molecular weight (Mw_{app}) was obtained by dividing the Porod volume by 1.7 and can be compared with the predicted molecular weight from sequence (Mw_{calc}), assuming a 1:1 complex.

	R_g (nm)	I_0	Volume (nm^3)	Mw_{app} (kDa)	Mw_{calc} (kDa)	D_{max} (nm)
HB3var03 CIDRα1.4	2.34	5643	42.6	25.0	26.1	6.5
EPCR	1.99	4727	38.9	22.9	20.0	5.5
HB3var03 CIDRα1.4:EPCR complex	2.63	9323	76.5	45.0	46.1	7.4
DD2var32 head (DBLα1.7-CIDRα1.4-DBLβ1	5.30	31700	263.9	155.2	140	18.7
DD2var32 head (DBLα1.7-CIDRα1.4-DBLβ1):EPCR complex	5.38	37550	309.0	181.8	160	18

Table S5: Crystallography data collection statistics – Related to Figure 2

	HB3var03 CIDRα1:EPCR			IT4var07 CIDRα1:EPCR		
Beamline	Diamond I-04			Diamond I-02		
Wavelength (Å)	0.98			0.9787		
Space Group	$C222_1$			$P3_221$		
Cell Parameters (Å)	a = 66.14, b = 94.72, c = 290.84; α = β = γ = 90			a = b = 56.02, c = 250.50; α = β = 90, γ = 120		
	Overall	Inner Shell	Outer Shell	Overall	Inner Shell	Outer Shell
Resolution (Å)	53.29-2.65	53.29-8.38	2.79-2.65	83.90-2.90	83.90-9.17	3.06-2.90
R_{mrg}	0.093	0.033	0.58	0.061	0.034	0.882
I/σ(I)	9.2	24.2	2.3	16.4	40	2.3
Completeness (%)	95.3	90.8	95.4	99.9	99.9	99.1
Multiplicity	3.6	3.5	3.6	8.4	7.1	6.9
Anomalous Completeness (%)	81.8	85.4	81.1	99.8	100	99
Anomalous Multiplicity	2.1	2	2	4.4	4.7	3.5

X-ray refinement statistics

	HB3var03 CIDRα1:EPCR	IT4var07 CIDRα1:EPCR
Resolution (Å)	2.65Å	2.90Å
Reflections used for refinement		
R_{work} (%)	22.2	24.7
R_{free} (%)	25.5	27.6
No. of protein residues in model	733	359
rmsd bond lengths (Å)	0.009	0.011
rmsd bond angles (°)	1.25	1.28
Ramachandran plot		
Allowed region	92.8	92.0
Additional allowed region	6.9	7.7
Generously allowed region	0.3	0.3
Disallowed region	0	0

Table S6: Affinities of mutants of the HB3var03 CIDRα1.4 domains for ECPR as measured by surface plasmon resonance – Related to Figure 3

Mutation	Range	K_D (nM)	k_{on} ($M^{-1}s^{-1}$)	k_{off} (s^{-1})
F651A	1uM-0.9nM	2.5	1.60×10^5	3.94×10^{-4}
F655L	1uM-0.9nM	4.3	1.45×10^5	6.30×10^{-4}
F655Y	1uM-0.9nM	0.5	1.10×10^6	5.72×10^{-4}
F656A	1uM-0.9nM	12.9	1.16×10^5	1.50×10^{-2}
F656V	1uM-0.9nM	1.5	1.45×10^5	2.23×10^{-4}
F656Y	1uM-0.9nM	1.9	3.29×10^5	3.79×10^{-4}
V658A	1uM-0.9nM	37	1.94×10^4	7.19×10^{-4}
W669S	1uM-0.9nM	11	4.01×10^4	4.49×10^{-4}
Q657K	1uM-0.9nM	72	6.89×10^4	4.99×10^{-3}
